# Protein engineering: status report

**DOI:** 10.1093/protein/gzag020

**Published:** 2026-07-17

**Authors:** Hui-wang Ai, Frances H Arnold, Doug Barrick, Hagan Bayley, Alfie-Louise R Brownless, Robert E Campbell, Roger Castells-Graells, Pimchai Chaiyen, Roberto A Chica, Lauren O Chisholm, Daniel Christ, Guo-Chao Chu, Michael Chungyoun, Vincent G H Eijsink, Michael B Elowitz, Douglas J Fansher, Rudi Fasan, Gabriel Foley, Michelle S Frei, Andreas Gagsteiger, Ruiyan Gao, Elizabeth M J Gillam, Jeffrey J Gray, Luis F Guerra, Birte Höcker, Zhiyang Jin, Shina Caroline Lynn Kamerlin, Nobuyasu Koga, Roland E Kontermann, Brian Kuhlman, Olivia A Langlois, Dongyang Li, Roberto D Lins, Chang C Liu, Lei Liu, Diego López Barreiro, Peilong Lu, Sohum Mehta, Ikumi Miyazaki, Manuel M Müller, Serge Muyldermans, Naoto Nemoto, Jeff Nivala, James K Nuñez, Izaiah J Ornelas, Joelle N Pelletier, Andreas Plückthun, Owen T Porth, Kridsadakorn Prakinee, Yujia Qing, Julia C Reisenbauer, Sandy Schmidt, Oliver Seifert, Mikhail G Shapiro, Rachna Sharma, Nilmani Singh, Benjamin J Smith, Jamie B Spangler, Rie Tatsumi-Koga, Takuya Terai, Henrik Terholsen, Mattias Tolhurst, Katherine W Tripp, Vanessa Tanja Trossmann, Chandra L Tucker, Onur Turak, James A Van Deventer, Anikó Várnai, Isabelle F T Viana, Amy M Weeks, K Dane Wittrup, Kingsley L-J Wong, Chenggang Xi, Todd O Yeates, Andy Hsien-Wei Yeh, Jin Zhang, Tiger S Zhang, Huimin Zhao, Fang Zheng, Jingyi Zhu, Sacha Zinn

**Affiliations:** Center for Membrane and Cell Physiology, Department of Molecular Physiology and Biological Physics, University of Virginia School of Medicine, Charlottesville, VA 22908, United States; Division of Chemistry and Chemical Engineering, California Institute of Technology, Pasadena, CA 91125, United States; T.C. Jenkins Department of Biophysics, Johns Hopkins University, Baltimore, MD 21218, United States; Department of Chemistry, University of Oxford, Oxford OX1 3TA, United Kingdom; School of Chemistry and Biochemistry, Georgia Institute of Technology, Atlanta, GA 30332, United States; Department of Chemistry, Graduate School of Science, The University of Tokyo, Hongo, Bunkyo-ku, Tokyo 113-0033, Japan; CERVO Brain Research Center and Department of Biochemistry, Microbiology, and Bioinformatics, Université Laval, Québec, QC G1J 2G3, Canada; Structural Biology Programme, Spanish National Cancer Research Centre (CNIO), Madrid 28029, Spain; School of Biomolecular Science and Engineering, Vidyasirimedhi Institute of Science and Technology (VISTEC), Wangchan Valley, Rayong 21210, Thailand; Department of Chemistry and Biomolecular Sciences, University of Ottawa, Ottawa, ON K1N 6N5, Canada; Department of Biomedical Engineering, Johns Hopkins University School of Medicine, Baltimore, MD 21218, United States; Translational Tissue Engineering Center, Johns Hopkins University School of Medicine, Baltimore, MD 21231, United States; Garvan Institute of Medical Research, Sydney, NSW 2010, Australia; St Vincent's Clinical School, Faculty of Medicine, The University of New South Wales, Sydney, NSW 2010, Australia; New Cornerstone Science Laboratory, Tsinghua-Peking Joint Center for Life Sciences, Ministry of Education Key Laboratory of Bioorganic Phosphorus Chemistry and Chemical Biology, Center for Synthetic and Systems Biology, Department of Chemistry, Tsinghua University, Beijing 100084, China; School of Food and Biological Engineering, Hefei University of Technology, Hefei 230009, China; Department of Chemical and Biomolecular Engineering, Johns Hopkins University, Baltimore, MD 21218, United States; Faculty of Chemistry, Biotechnology and Food Science, Norwegian University of Life Sciences (NMBU), 1432 Ås, Norway; Division of Biology and Biological Engineering, California Institute of Technology, Pasadena, CA 91125, United States; Howard Hughes Medical Institute, California Institute of Technology, Pasadena, CA 91125, United States; Department of Chemistry, Université de Montréal, Montréal, QC H2V 0B3, Canada; PROTEO, The Quebec Network for Research on Protein Function, Engineering, and Applications, Montréal, QC G1V 0A6, Canada; Center in Green Chemistry and Catalysis, Montréal, QC H2V 0B3, Canada; Department of Chemistry and Biochemistry, The University of Texas at Dallas, Richardson, TX 75080, United States; Samsara Eco, Sydney, NSW 2065, Australia; Department of Chemistry and Applied Biosciences, ETH Zürich, Zürich 8093, Switzerland; Department of Biochemistry, University of Bayreuth, Bayreuth 95447, Germany; Manufacturing Futures Lab, Department of Chemical Engineering, University College London, London WC1E 7JE, United Kingdom; Centre for Nature-Inspired Engineering, Department of Chemical Engineering, University College London, London WC1E 7JE, United Kingdom; School of Chemistry and Molecular Biosciences, The University of Queensland, St. Lucia, Brisbane 4072, Australia; Department of Chemical and Biomolecular Engineering, Johns Hopkins University, Baltimore, MD 21218, United States; Program in Molecular Biophysics, Institute for Nanobiotechnology, and Center for Computational Biology, Johns Hopkins University, Baltimore, MD 21218, United States; Department of Chemistry, King’s College London, London SE1 1DB, United Kingdom; Department of Biochemistry, University of Bayreuth, Bayreuth 95447, Germany; Andrew and Peggy Cherng Department of Medical Engineering, California Institute of Technology, Pasadena, CA 91125, United States; School of Chemistry and Biochemistry, Georgia Institute of Technology, Atlanta, GA 30332, United States; School of Chemical and Biomolecular Engineering, Georgia Institute of Technology, Atlanta, GA 30332, United States; Department of Chemistry, Lund University, Lund 221 00, Sweden; Laboratory for Protein Design, Institute for Protein Research, The University of Osaka, Suita, Osaka 565-0871, Japan; Protein Design Group, Exploratory Research Center on Life and Living Systems, National Institutes of Natural Sciences, Okazaki, Aichi 444-8585, Japan; Institute of Cell Biology and Immunology, University of Stuttgart, Stuttgart 70569, Germany; Department of Biochemistry and Biophysics, University of North Carolina at Chapel Hill, Chapel Hill, NC 27599, United States; T.C. Jenkins Department of Biophysics, Johns Hopkins University, Baltimore, MD 21218, United States; Biological and Biomedical Sciences Program, Harvard University, Boston, MA 02115, United States; Division of Biology and Biological Engineering, California Institute of Technology, Pasadena, CA 91125, United States; Aggeu Magalhaes Institute, Oswaldo Cruz Foundation, Recife 50740-465, Brazil; Department of Biomedical Engineering, University of California Irvine, Irvine, CA 92697, United States; New Cornerstone Science Laboratory, Tsinghua-Peking Joint Center for Life Sciences, Ministry of Education Key Laboratory of Bioorganic Phosphorus Chemistry and Chemical Biology, Center for Synthetic and Systems Biology, Department of Chemistry, Tsinghua University, Beijing 100084, China; Manufacturing Futures Lab, Department of Chemical Engineering, University College London, London WC1E 7JE, United Kingdom; Centre for Nature-Inspired Engineering, Department of Chemical Engineering, University College London, London WC1E 7JE, United Kingdom; New Cornerstone Science Laboratory, State Key Laboratory of Gene Expression, Research Center for Industries of the Future, School of Life Sciences, Westlake University, Hangzhou, Zhejiang 310024, China; Department of Pharmacology, University of California San Diego, La Jolla, CA 92093, United States; Department of Chemistry, Graduate School of Science, The University of Tokyo, Hongo, Bunkyo-ku, Tokyo 113-0033, Japan; Department of Chemistry, King’s College London, London SE1 1DB, United Kingdom; Laboratory of Cellular and Molecular Immunology, Vrije Universiteit Brussel, Brussels 1050, Belgium; Graduate School of Science and Engineering, Saitama University, Sakura-ku, Saitama 338-8570, Japan; Department of Research and Development, Epsilon Molecular Engineering Inc., Sakura-ku, Saitama 338-8570, Japan; Paul G. Allen School of Computer Science, Molecular Engineering and Sciences Institute, University of Washington, Seattle, WA 98195, United States; Department of Molecular and Cell Biology, University of California, Berkeley, Berkeley, CA 94720, United States; Biohub, San Francisco, San Francisco, CA 94158, United States; Department of Molecular and Cell Biology, University of California, Berkeley, Berkeley, CA 94720, United States; Biohub, San Francisco, San Francisco, CA 94158, United States; Department of Chemistry, Université de Montréal, Montréal, QC H2V 0B3, Canada; PROTEO, The Quebec Network for Research on Protein Function, Engineering, and Applications, Montréal, QC G1V 0A6, Canada; Center in Green Chemistry and Catalysis, Montréal, QC H2V 0B3, Canada; Department of Biochemistry and Molecular Medicine, Université de Montréal, Montréal, QC H3T 1J4, Canada; Department of Biochemistry, University of Zürich, Zürich 8057, Switzerland; Koch Institute for Integrative Cancer Research, Massachusetts Institute of Technology, Cambridge, MA 02139, United States; Department of Biological Engineering, Massachusetts Institute of Technology, Cambridge, MA 02139, United States; School of Biomolecular Science and Engineering, Vidyasirimedhi Institute of Science and Technology (VISTEC), Wangchan Valley, Rayong 21210, Thailand; Helmholtz Institute for Pharmaceutical Research Saarland, Saarbrücken 66123, Germany; Department of Chemistry, University of Oxford, Oxford OX1 3TA, United Kingdom; Division of Chemistry and Chemical Engineering, California Institute of Technology, Pasadena, CA 91125, United States; Institute of Science and Technology Austria (ISTA), Am Campus 1, 3400 Klosterneuburg, Austria; Department of Chemical and Pharmaceutical Biology, Groningen Research Institute of Pharmacy, University of Groningen, Groningen 9713, AV, The Netherlands; Institute of Cell Biology and Immunology, University of Stuttgart, Stuttgart 70569, Germany; Division of Chemistry and Chemical Engineering, California Institute of Technology, Pasadena, CA 91125, United States; Andrew and Peggy Cherng Department of Medical Engineering, California Institute of Technology, Pasadena, CA 91125, United States; Howard Hughes Medical Institute, California Institute of Technology, Pasadena, CA 91125, United States; Center for Membrane and Cell Physiology, Department of Molecular Physiology and Biological Physics, University of Virginia School of Medicine, Charlottesville, VA 22908, United States; Carl R. Woese Institute for Genomic Biology, University of Illinois Urbana-Champaign, Urbana-Champaign, IL 61801, United States; NSF iBioFoundry, University of Illinois Urbana-Champaign, Urbana-Champaign, IL 61801, United States; DOE Center for Advanced Bioenergy and Bioproducts Innovation, University of Illinois Urbana-Champaign, Urbana-Champaign, IL 61801, United States; NSF Global Center for Biofoundry Applications, University of Illinois Urbana-Champaign, Urbana-Champaign, IL 61801, United States; Department of Chemistry and Biomolecular Sciences, University of Ottawa, Ottawa, ON K1N 6N5, Canada; Department of Biomedical Engineering, Johns Hopkins University School of Medicine, Baltimore, MD 21218, United States; Translational Tissue Engineering Center, Johns Hopkins University School of Medicine, Baltimore, MD 21231, United States; Department of Chemical and Biomolecular Engineering, Johns Hopkins University, Baltimore, MD 21218, United States; Bloomberg-Kimmel Institute for Cancer Immunotherapy, Johns Hopkins University, Baltimore, MD 21287, United States; Sidney Kimmel Comprehensive Cancer Center, Johns Hopkins University, Baltimore, MD 21287, United States; Department of Oncology, Johns Hopkins University School of Medicine, Baltimore, MD 21205, United States; Department of Ophthalmology, Johns Hopkins University School of Medicine, Baltimore, MD 21287, United States; Department of Molecular Microbiology & Immunology, Johns Hopkins University Bloomberg School of Public Health, Baltimore, MD 21205, United States; Laboratory for Protein Design, Institute for Protein Research, The University of Osaka, Suita, Osaka 565-0871, Japan; Department of Chemistry, Graduate School of Science, The University of Tokyo, Hongo, Bunkyo-ku, Tokyo 113-0033, Japan; Department of Chemical and Pharmaceutical Biology, Groningen Research Institute of Pharmacy, University of Groningen, Groningen 9713, AV, The Netherlands; Paul G. Allen School of Computer Science, Molecular Engineering and Sciences Institute, University of Washington, Seattle, WA 98195, United States; T.C. Jenkins Department of Biophysics, Johns Hopkins University, Baltimore, MD 21218, United States; Department of Biochemistry, University of Bayreuth, Bayreuth 95447, Germany; Department of Pharmacology, University of Colorado School of Medicine, Aurora, CO 80045, United States; Department of Biochemistry, University of Bayreuth, Bayreuth 95447, Germany; Chemical and Biological Engineering Department, Tufts University, Medford, MA 02155, United States; Biomedical Engineering Department, Tufts University, Medford, MA 02155, United States; Faculty of Chemistry, Biotechnology and Food Science, Norwegian University of Life Sciences (NMBU), 1432 Ås, Norway; Department of Fundamental Chemistry and Materials Science Graduate Program, Federal University of Pernambuco, Recife 50670-901, Brazil; Departments of Biochemistry and Chemistry, University of Wisconsin—Madison, Madison, WI 53705, United States; Koch Institute for Integrative Cancer Research, Massachusetts Institute of Technology, Cambridge, MA 02139, United States; Department of Biological Engineering, Massachusetts Institute of Technology, Cambridge, MA 02139, United States; Department of Chemical Engineering, Massachusetts Institute of Technology, Cambridge, MA 02139, United States; Paul G. Allen School of Computer Science, Molecular Engineering and Sciences Institute, University of Washington, Seattle, WA 98195, United States; Department of Biomolecular Engineering, University of California Santa Cruz, Santa Cruz, CA 95064, United States; Department of Chemistry and Biochemistry, University of California Los Angeles, Los Angeles, CA 90095, United States; Department of Biomolecular Engineering, University of California Santa Cruz, Santa Cruz, CA 95064, United States; Department of Pharmacology, University of California San Diego, La Jolla, CA 92093, United States; Shu Chien-Gene Lay Department of Bioengineering, University of California San Diego, La Jolla, CA 92093, United States; Department of Biochemistry and Molecular Biophysics, University of California San Diego, La Jolla, CA 92093, United States; Moores Cancer Center, University of California San Diego, La Jolla, CA 92093, United States; Department of Biochemistry and Biophysics, University of North Carolina at Chapel Hill, Chapel Hill, NC 27599, United States; Carl R. Woese Institute for Genomic Biology, University of Illinois Urbana-Champaign, Urbana-Champaign, IL 61801, United States; NSF iBioFoundry, University of Illinois Urbana-Champaign, Urbana-Champaign, IL 61801, United States; DOE Center for Advanced Bioenergy and Bioproducts Innovation, University of Illinois Urbana-Champaign, Urbana-Champaign, IL 61801, United States; NSF Global Center for Biofoundry Applications, University of Illinois Urbana-Champaign, Urbana-Champaign, IL 61801, United States; Department of Chemical and Biomolecular Engineering, University of Illinois Urbana-Champaign, Urbana-Champaign, IL 61801, United States; Department of Biochemistry and Molecular Biology, Health Science Center, Xi’an Jiaotong University, Xi’an 710061, China; New Cornerstone Science Laboratory, State Key Laboratory of Gene Expression, Research Center for Industries of the Future, School of Life Sciences, Westlake University, Hangzhou, Zhejiang 310024, China; Garvan Institute of Medical Research, Sydney, NSW 2010, Australia; St Vincent's Clinical School, Faculty of Medicine, The University of New South Wales, Sydney, NSW 2010, Australia

**Keywords:** experimental techniques, computational methods, application areas

## Abstract

With this status report, we aim to provide a timely snapshot of the protein engineering field as a broad and rapidly advancing discipline that integrates computational, molecular biology, structure-guided, evolutionary, and synthetic approaches to create new and improved proteins with tailored structures and useful functions. The report is organized into eight thematic areas spanning core methodologies and major application domains, including enzymes, therapeutics, detection, synthetic biology, and materials. Contributions from experts across these areas highlight both the historical foundations and recent advances in their respective fields, with particular emphasis on the growing influence of machine learning and artificial intelligence-based methods. Emerging from this broad overview is a central message: protein engineering appears to be entering a golden age, defined by a rapidly accelerating pace of progress, even as significant challenges in design, screening, and real-world application remain. Looking ahead, the continued integration of computational and experimental strategies is poised to further accelerate the impact of protein engineering across an expanding range of economically and societally important sectors, from therapeutics and molecular imaging to diagnostics, plastic recycling, and industrial chemistry.

## Introduction

The field of protein engineering emerged in the early 1980s, enabled by the revolutionary advances in protein structure determination, molecular biology, chemical DNA synthesis, and computer modeling, that had taken place during the preceding decade (Ulmer 1983; Nagai 1985). A concrete and proactive milestone marking the establishment of protein engineering as a distinct field was the launch of the journal *Protein Engineering* in 1986 under the founding editorship of Gregory A. Petsko and Anthony R. Rees. The journal was renamed *Protein Engineering, Design, and Selection* in 2004, coincident with a transition to a new senior editorial team of Sir Alan R. Fersht, Valerie Daggett, and Sir Gregory P. Winter.

At the time of the inaugural issue of *Protein Engineering*, the phrase “protein engineering” had appeared fewer than 30 times in the scientific literature, based on a PubMed search conducted in December 2025. This count undoubtedly underestimates the intellectual roots of the field, as many early studies would now be classified as protein engineering even if they did not use the term explicitly (Leatherbarrow and Fersht 1986; Wetzel 1986). In their inaugural editorial (Rees and Petsko 1986), Rees and Petsko argued persuasively that protein engineering represented more than a transient fad and should instead be regarded as a distinct scientific discipline. They also expressed optimism regarding the eventual emergence of practical applications from protein engineering approaches, a view that stood in stark contrast to contemporary characterizations of protein engineering as a “pipe-dream” (Dover 1977) or even as “protein terrorism” (Rees and Petsko 1986).

It did not take long for Rees and Petsko’s optimism to prove prescient (Knowles 1987). Indeed, before the end of the 1980s, teams led by Brian W. Matthews and Sir Alan R. Fersht had successfully applied protein engineering to unravel principles of protein stability (Matthews *et al*. 1987; Kellis *et al.* 1988; Serrano and Fersht 1989; Matthews 1993) and reports of enzyme variants with drastically improved stability were appearing in the literature (Matsumura *et al*. 1989; Van den Burg *et al.* 1998). Also, companies such as Genentech and Novozymes had demonstrated the commercial relevance of protein engineering, with engineered proteases featuring improved thermal and oxidative stability entering the washing powder market in the late 1980s (Estell *et al.* 1985; Wells and Estell 1988).

Also appearing in the inaugural issue was a commentary by Ronald Wetzel entitled “What is protein engineering?” (Wetzel 1986). In an attempt to answer this question, Wetzel surveyed the contemporary literature and proposed three broad classes of experiments that together constituted a working definition of protein engineering, along with a fourth class that the field might aspire to achieve. These first three classes included: (1) applications guided by primary sequence information; (2) applications relying on screening or selection of randomly generated variants; and (3) applications grounded in elucidated relationships between tertiary structure and function. The aspirational fourth class was the complete design and synthesis of proteins with improved or novel function. Using terminology that is more common today, these four classes might be restated as: (1) bioinformatics-guided engineering; (2) directed evolution and selection using *in vitro* or cell-based display; (3) rational structure-guided engineering; and (4) *de novo* protein design. Strikingly, Wetzel’s framework continues to hold up well, as these four classes collectively encompass essentially all of the major methodologies that define modern protein engineering, as will be described later in this article.

After more than four decades as an established discipline, protein engineering has expanded into a broad and increasingly branched field, with distinct areas that now resemble disciplines in their own right. Each of the four methodological classes articulated by Wetzel (Wetzel 1986) have undergone tremendous development, with several advances recognized by Nobel Prizes. Powerful molecular biology and directed evolution techniques are now routine, computational protein design has become broadly accessible, and sophisticated instrumentation and robotics enable high-throughput screening and selection at scales that would have been inconceivable in the early years of the field. Against this backdrop, protein engineering appears to be entering a golden age, with rapid methodological advances transforming what is possible, even as major challenges in functional design, library screening, and clinical translation, among others, continue to guide efforts in the field.

In light of the long-term growth of the field, its recent acceleration driven by powerful algorithms for *de novo* protein design, and its continued expansion into new application domains, the present moment seemed an ideal time to take a snapshot of protein engineering as a whole. The goal of this status report is to provide such a snapshot. Given the breadth of the field, its overlap with other disciplines, and the hazy boundaries between subdisciplines, we have necessarily taken a somewhat rough and arbitrary approach by dividing this report into eight thematic areas. Three of these are primarily method-focused: experimental techniques (excluding computational methods), computational methods, and synthetically augmented proteins. The remaining five are application-focused: enzymes; antibodies and therapeutics; detection, imaging, and diagnostics; synthetic biology and control systems; and materials, nanostructures, and machines. Each thematic area is further subdivided into sections authored by experts in the corresponding subfields. While this organization is neither fully systematic nor exhaustive, we believe it represents an effective approach to capturing the breadth, diversity, and current momentum of the field.

Despite our efforts to provide a thorough and inclusive overview, we acknowledge that this status report is incomplete and there are other relevant topics that could have been included in this report. These include, but are not limited to, catalytic antibodies (Lerner *et al*. 1991); repurposing of the ribosome for the synthesis of alternative polymers (Liu *et al*. 2017); DREADDs and related chemogenetic tools (Kang *et al.* 2023); engineering viral capsids for therapeutic applications (Kotterman and Schaffer 2014); protein condensates and liquid–liquid phase separation (Qian *et al*. 2022a); protein manufacturing and scale-up (Molowa and Mazanet 2003; Szkodny and Lee 2022); and ethical and societal considerations relevant to protein engineering and synthetic biology (Esvelt 2018; Adamala *et al.* 2024). We direct interested readers to the literature cited above for further details of these areas.

## Experimental techniques

### Directed evolution for engineering new-to-nature enzyme reactions (Julia C. Reisenbauer and Frances H. Arnold^*^)

**Figure 1:**
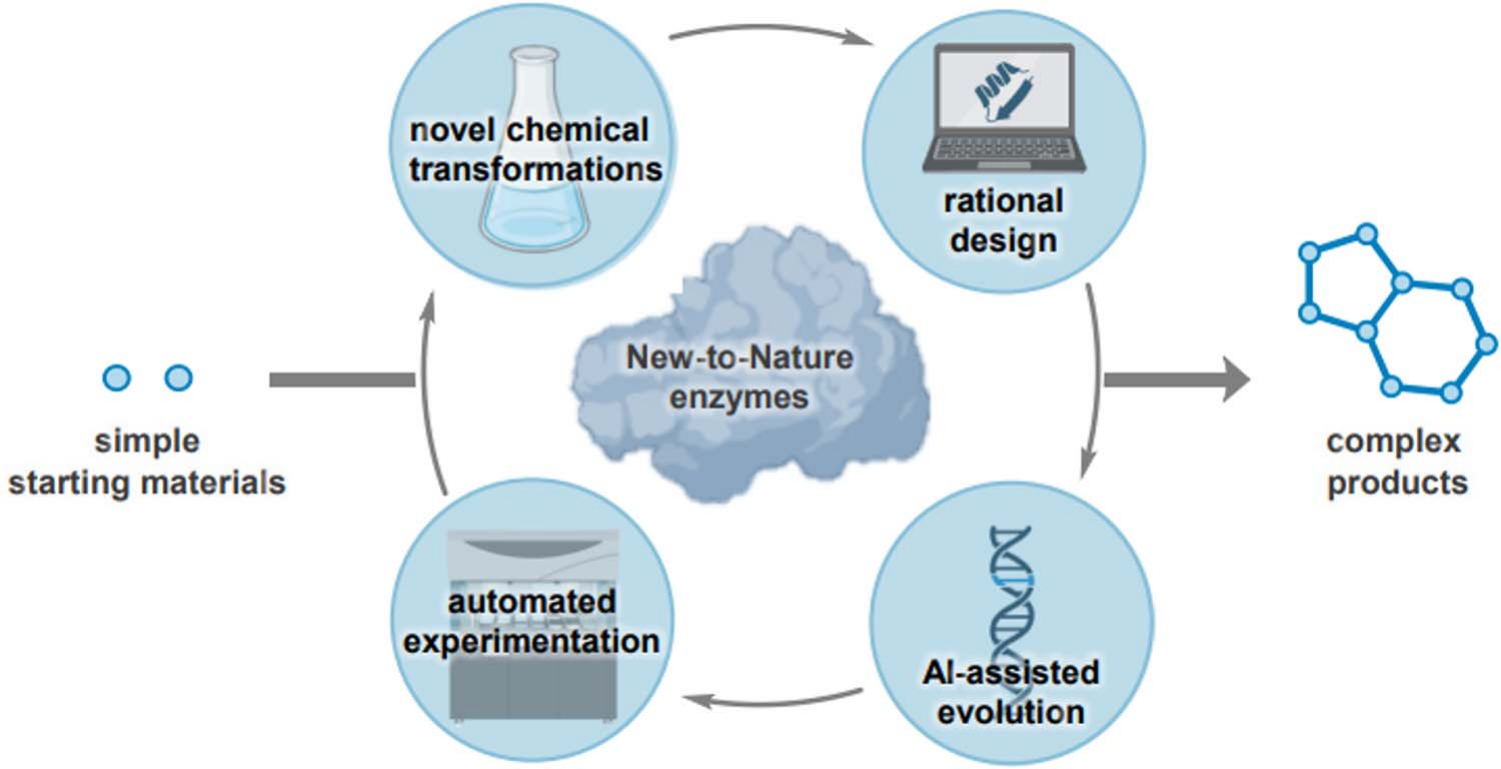
Design and evolution of new-to-nature enzymes. New-to-nature enzymes enable the conversion of simple and naturally abundant starting materials into complex, high-value products.

Nature’s highly efficient catalysts, the enzymes, orchestrate the construction (and deconstruction) of complex biological molecules and materials (Fig. 1). Thanks to improvements in methods to manipulate DNA sequences and express their products in recombinant organisms (Goodwin *et al.* 2016; Hoose *et al.* 2023), researchers can now envision engineering these remarkable molecular machines for industrial and synthetic applications that require functions well beyond natural enzymes’ capabilities (Reetz 2013; Arnold 2018). Directed evolution is a powerful strategy for engineering enzymes using rounds of mutagenesis and artificial selection to confer novel and useful traits (Wang *et al.* 2021a). The ability to engineer more robust and versatile biocatalysts has fundamentally shifted the perception of enzymes from being delicate catalysts to industrially relevant tools (Buller *et al.* 2023). In recent years, directed evolution has generated enzymes capable of catalyzing reactions far beyond their native reactivities and specificities, thereby opening new avenues for innovative biotechnological applications.

Enzymes improved by directed evolution have enabled groundbreaking applications across sustainable chemical synthesis, bioremediation, and medicine. Examples include producing biofuels, pharmaceuticals like islatravir (Huffman *et al.* 2019), and fine chemicals (Wu *et al.* 2021) with enzymes engineered to enhance activity, selectivity, and stability under non-natural conditions important for manufacturing (Woodley 2022). Enzymes are now widely adopted in industrial processes, offering greener and more efficient alternatives to traditional chemical methods. An emerging focus is engineering enzymes to exhibit activities not known in nature: the growing repertoire of these new-to-nature enzymes is further expanding their applications in biosynthesis and biocatalysis (Emmanuel *et al.* 2016; Jeschek *et al.* 2016; Biegasiewicz *et al.* 2019; Huang *et al.* 2020; Rui *et al.* 2022; Trimble *et al.* 2022; Tseliou *et al.* 2024; Wang *et al.* 2024a; Xing *et al.* 2025). Some of these new enzymes can even catalyze reactions that have not been reported previously, in chemistry or biology (Gao *et al.* 2023).

Directed evolution is highly effective on smooth sequence-fitness landscapes, but faces obstacles when navigating rugged ones, rendering optimization resource- and time-intensive and often limited to local optima (Romero and Arnold 2009). To overcome these challenges and unlock the full potential of evolutionary enzyme engineering, multiple groups are developing higher-throughput screens and even continuous evolution methods (Molina *et al.* 2022; Gantz *et al.* 2023; Chen *et al.* 2024b). Currently, however, the broader adoption of these methodologies remains constrained by the stringent requirements for developing robust and reliable enzyme screening assays. In parallel, machine learning (ML)-assisted directed evolution can leverage higher-throughput experimental data to guide variant optimization (Yang, Li and Arnold 2024). While these approaches offer considerable potential, they remain costly to scale and are still constrained by experimental bottlenecks, including limitations in analytical throughput. Beyond optimization, artificial intelligence (AI) is now also being used to generate promising enzymatic starting points, e.g. through protein language models fine-tuned on related enzyme families (Madani *et al.* 2023; Ruffolo *et al.* 2025) or diffusion models conditioned on the structure of a desired active site (Albanese *et al.* 2025; Lauko *et al.* 2025; Listov *et al.* 2025; Ahern *et al.* 2026). These approaches exploit statistical regularities in vast amounts of sequence or structure data (and some sequence-function data) for enzyme design and optimization, moving beyond nature’s relatively conservative, fully empirical evolutionary processes (Lovelock *et al.* 2022). Together, high-quality data generation, automated experimentation (see Section Automation and robotics (Nilmani Singh and Huimin Zhao^*^)), and powerful generative algorithms promise to accelerate the discovery of novel biocatalysts, potentially enabling the genetic encoding of almost any desired chemical transformation and providing visionary new solutions to sustainable manufacturing and novel chemical reactions.

### Continuous protein evolution (Chang C. Liu^*^)

**Figure 2:**
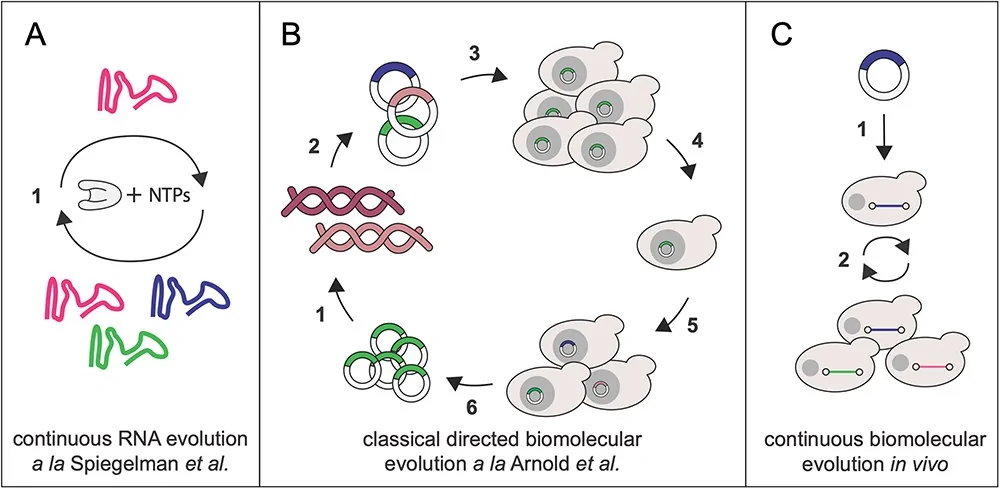
Modes of biomolecular evolution, from continuous to classical to continuous. (A) RNA evolution for replication (or reactions coupled to RNA replication) can be carried out in a test tube continuously just by passaging RNA in the presence of the replicase and nutrients (1). (B) Broader range biomolecular evolution can be carried out in a cell-reliant manner through iterations of the classical directed evolution cycle involving diversification of genes in a test tube (1), cloning the resulting library into a vector (2), transforming the library into cells where biomolecules can be expressed (3), selection for new or improved function by screening or selection (4), amplification of improved variants as cells replicate (5), and isolation of “winning” variants (6). (C) Broader range continuous evolution can be carried out *in vivo* by cloning genes into a continuously hypermutating genetic system (1) such that evolution of new or improved function can occur simply as cells grow (2).

In the late 1960s, Sol Spiegelman serially passaged the RNA genome of bacteriophage Qβ in test tubes containing Qβ’s replicase (i.e. an RNA-dependent RNA polymerase) and nucleotide triphosphates (NTPs) (Fig. 2A) (Mills *et al*. 1967). Fueled by the high mutation rate of the replicase, Spiegelman’s RNA quickly evolved into a shortened molecule that propagated 15-fold faster than its ancestor. This historic experiment demonstrated that an isolated biomolecule could rapidly evolve a well-defined molecular property—efficient binding to and copying by the replicase—in the laboratory. Combined with additional experiments that successfully challenged the RNA to evolve molecular properties such as resistance to ethidium bromide (Saffhill *et al.* 1970), Spiegelman’s work presaged the application of evolution as a biomolecular engineering tool.

The distance between evolving a (low-information) RNA molecule that recruits an externally supplied (high-information) replicase and the maturity of evolutionary biomolecular engineering today is long. This distance was primarily closed by the directed evolution “pipeline,” developed by pioneers such as Arnold (Chen and Arnold 1993) and Stemmer (Stemmer 1994), where biomolecule-encoding genes are subjected to cycles of *in vitro* diversification (e.g. by error-prone PCR) followed by transformation into cells that express the diversified gene libraries and selection (Fig. 2B). By leveraging cells, biomolecules requiring complex expression machinery (e.g. proteins that necessitate translation) became admissible for evolution as did a wealth of target molecular functions that could be linked to cellular systems and fitness. With this now-classical directed evolution pipeline, biomolecular evolution grew beyond evolving RNAs for replication into a general biomolecular engineering strategy.

Yet the directed evolution pipeline came with a loss. Like natural evolution, Spiegelman’s experiment reflected a continuous process wherein the diversification and selective amplification steps defining Darwinian evolution occurred autonomously. In contrast, the classical directed evolution pipeline manually stages diversification and selective amplification as discrete steps. The difference is not just aesthetic. Inherent in the manual staging are limitations on diversity (e.g. through transformation efficiency bottlenecks), depth in sequence search (e.g. each labor-intensive cycle of diversification, transformation, and selection is only one step in an evolutionary trajectory), and experimental scale (e.g. only a few independent evolution experiments can be practically maintained). These limitations are largely avoided in continuous modes of evolution.

The missing continuousness in the classical directed evolution pipeline spurred the establishment of continuous evolution systems (Rix and Liu 2021) that carry out rapid diversification of chosen genes inside cells, effecting the conversion of the directed evolution pipeline into an autonomous one where user-defined biomolecules evolve just as cells are passed under selection (much as passaging RNA extracellularly within the setups of Spiegelman and others (Wright and Joyce 1997) resulted in the continuous evolution of new RNA molecular functions) (Fig. 2C). Since the mutation rates of large cellular genomes are necessarily low and untargeted to any particular gene, this conversion required the technical development of hypermutation systems that selectively and durably drive autonomous diversification of target genes *in vivo*. Durable targeted hypermutation was (indirectly) realized in Phage-Assisted Continuous Evolution (PACE) (Esvelt *et al*. 2011) by linking the activity of user-defined genes to phage propagation through a dispensable broadly mutagenic host and more directly achieved with genetic systems such as orthogonal DNA replication (OrthoRep) (Ravikumar *et al*. 2014; Ravikumar *et al.* 2018; Rix *et al.* 2024), its relatives such as EcORep (Tian *et al.* 2024), and additional systems like MutaT7 (Moore *et al.* 2018) and more (Molina *et al.* 2022; He *et al.* 2025) (of varying degrees of durability). The application of these continuous evolution systems has led to scores of biomolecular engineering successes including the evolution of novel gene editors, enzymes, biosensors, and antibodies (Morrison *et al.* 2020; Molina *et al.* 2022). Several recent experiments have also exploited continuous evolution systems for protein evolutionary data generation (Ravikumar *et al.* 2018; Rix *et al.* 2024; Alcantar *et al.* 2025), a forward-looking direction that should combine synergistically with statistical learning-based protein design by providing large volumes (scale) of out-of-distribution (depth) evolutionary outcomes to retrain generative design models for greater domain-specific and general accuracy. Moreover, the natural pairing between continuous evolution and automation (Zhong *et al.* 2020; DeBenedictis *et al.* 2022), for which serial passaging is straightforward, may act to further multiply both the application power and the data-generation capabilities of continuous evolution in our increasingly high-tech age.

The field of biomolecular evolution has come a long way since the early test tube RNA evolution experiments of Spiegelman and the first protein directed evolution experiments of Arnold, Stemmer, Smith, Winter, Lerner, and Schultz, among others. The development of *in vivo* continuous evolution adds to this history by unifying evolution à la Spiegelman—continuous but limited to RNA replication—with the directed evolution pipeline—applicable to proteins and a flexibility of functions but discontinuous and manually staged. This unification, and the depth and scale of biomolecular sequence search it affords, has already proven valuable in applications. It promises even greater impact ahead as the surrounding ecosystem of AI-based protein engineering begins to exploit the unique data volume and distributions continuous evolution can generate.

### *In vitro* display (Takuya Terai and Naoto Nemoto^*^)

*In vitro* display technologies such as phage display have now become indispensable for antibody drug development. However, the origins of *in vitro* evolution, the foundation of *in vitro* display technologies, are linked to research on the origin of life and the molecular evolution of proteins. Historically, *in vitro* molecular evolution was first performed using bacteriophage Qβ RNA replicase in 1967 by Spiegelman’s group (Mills *et al*. 1967). Then, some pioneering theoretical and experimental works by Eigen (Eigen and Schuster 1977) and Husimi (Husimi *et al.* 1982; Nemoto and Husimi 1995) discussed molecular evolution prior to the advent of cells on Earth. These studies highlighted the importance of “genotype-phenotype linkage.” In RNA, “genotype” and “phenotype” intrinsically reside in the same molecule. However, for proteins to evolve, RNA or DNA (the genotype) must be somehow linked to the phenotype of the encoded protein. Simple viruses represent a well-designed form of this linkage, i.e. physical coupling. On the other hand, cellular organisms rely on compartmentalization to achieve this linkage. Indeed, Husimi *et al.* conducted the first viral evolution experiment in 1982 using a continuous reactor with the filamentous phage fd (a virus that infects bacteria *Escherichia coli*) (Husimi *et al.* 1982). Based on this background, in 1985, Smith reported the first *in vitro* display technology, phage display, which became useful for bioengineering (Smith 1985). Here we define “*in vitro* display” as a method where the target protein to be engineered is displayed on the surface of a non-cellular entity.

**Figure 3:**
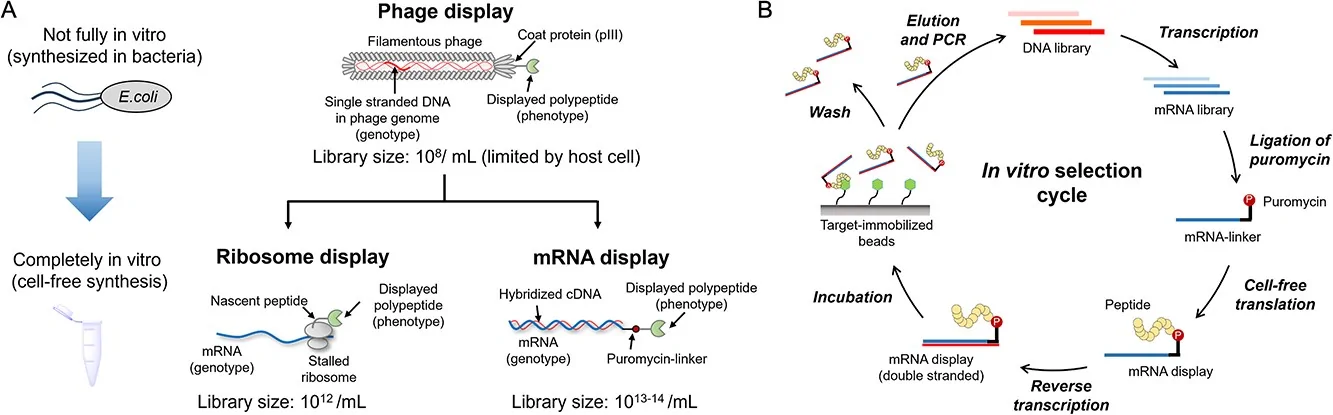
Representative *in vitro* display technologies. (A) From phage display to ribosome display and mRNA display. Because bacteriophages need to be constructed in *E. coli*, the diversity of the phage display library is limited by the size of *E. coli*. On the other hand, ribosome display and mRNA display do not have this limitation, so they can achieve much higher diversity. (B) Schematic diagram of *in vitro* selection, using mRNA display as an example. Due to the enormous library size, the selection cycle is typically repeated multiple times until highly active variants become dominant.

In Smith’s phage display, an exogenous peptide is fused to the pIII protein of the filamentous bacteriophage. This places the peptide on the virus’s surface, with the DNA encoding it located inside (Fig. 3A, top). The scope of this technique was later expanded by Winter *et al.* to present a part of antibody (single-chain variable fragment, i.e. scFv), which greatly contributed to the development of antibody therapeutics (McCafferty *et al.* 1990a). Smith and Winter were awarded the 2019 Nobel Prize in Chemistry for these achievements. In 1990s, two other representative *in vitro* display technologies, ribosome display (Mattheakis *et al.* 1994; Hanes and Plückthun 1997) and mRNA display (Nemoto *et al.* 1997; Roberts and Szostak 1997) (Fig. 3A, bottom), were developed for completely *in vitro* processes while keeping the essence of phage display, i.e. physically coupling nucleic acids and peptides. For this reason, in the first paper (Nemoto *et al.* 1997), “mRNA display” was named “*in vitro* virus.” These methods are groundbreaking in using cell-free translation systems. Specifically, mRNA display achieves a covalent coupling between the polypeptide translated in the cell-free system and its coding mRNA as a single molecule. This is realized by positioning the antibiotic, puromycin, which covalently binds to the C-terminus of the nascent peptide within the ribosome, near the 3′ end of mRNA *via* some DNA residues that stalls the ribosome and mRNA lacking a stop codon. Among established display technologies, mRNA display can handle the largest library sizes (10^13^–10^14^/ml) and has undergone various technical improvements. Ribosome display achieves the same by trapping the mRNA–ribosome–protein complexes directly (Plückthun 2012). Derivatives of mRNA display include cDNA display (Yamaguchi *et al.* 2009; Terai *et al.* 2019) that features enhanced chemical and biological stability, and the RAPID system (Goto and Suga 2021) that enables incorporation of diverse unnatural amino acids (UAAs). Other *in vitro* display technologies include CIS display (Odegrip *et al.* 2004) and DNA display (Yonezawa *et al.* 2003) that utilizes *in vitro* compartmentalization (Tawfik and Griffiths 1998). For more details, please refer to another review (Contreras-Llano and Tan 2018).

The most representative application of *in vitro* display is the selection of antibodies and peptides that bind to target proteins from random libraries (Fig. 3B). For example, medium-sized pharmaceuticals made from cyclic peptides containing UAAs, which are developed by the RAPID system and its modifications, have been attracting attention as next-generation pharmaceuticals (Tanada *et al.* 2023). Other reported applications include antibody epitope mapping (Scott and Smith 1990), generation of chemo-optogenetic tool proteins (Miyazaki *et al.* 2026), evaluation of protein folding stability (Tsuboyama *et al.* 2023), novel enzyme discovery (Seelig and Szostak 2007), rapid enzyme optimization (Munaweera *et al.* 2026), and enzyme substrate discovery (Damnjanovic *et al.* 2022; Chang *et al.* 2023). Compared to the technologies using cells, *in vitro* display can cover substantially greater diversity, and it allows selections even under non-physiological conditions where cells cannot survive. On the other hand, when one wants to modify the signaling networks consisting of multiple proteins or optimize the parameters other than binding affinity to the targets, such as enzyme activities in general, combinations with cell-based approaches may be required. *In vitro* display methods, which can handle and evaluate the characteristics of large-scale protein libraries made by cell-free translation as polypeptides themselves (not as one of the many proteins in a cell), well fits data science such as AI (Chang *et al.* 2023; Tsuboyama *et al.* 2023), and future developments in this direction are anticipated.

### Cell-based display (Owen T. Porth and K. Dane Wittrup^*^)

Cell-based protein display refers to a set of techniques that anchor a protein to the surface of a cell that encapsulates said protein’s encoding DNA. This coupling of a single genotype to phenotype facilitates high-throughput screening of billions of protein variants in diverse directed evolution experiments. George P. Smith originated the concept in 1985 when he invented the technique of phage display (see Section *In vitro* display (Takuya Terai and Naoto Nemoto^*^)), for which he shared the 2018 Nobel Prize (Smith 1985). Bacterial display and yeast surface display followed in the 1990s (Boder and Wittrup 1997; Georgiou *et al.* 1997). When coupled with magnetic selection and fluorescence activated cell sorting (FACS), these latter technologies allow quantifiable selection of improved protein variants from billion-member libraries. Eukaryotic yeast more reliably handles the post-translational modifications (PTMs) in relevant mammalian proteins like antibody fragments; however, mammalian display was invented in 2008 to address the non-human glycosylation patterns and occasional misfolding of complex mammalian proteins that can occur with yeast surface display (Beerli *et al.* 2008).

**Figure 4:**
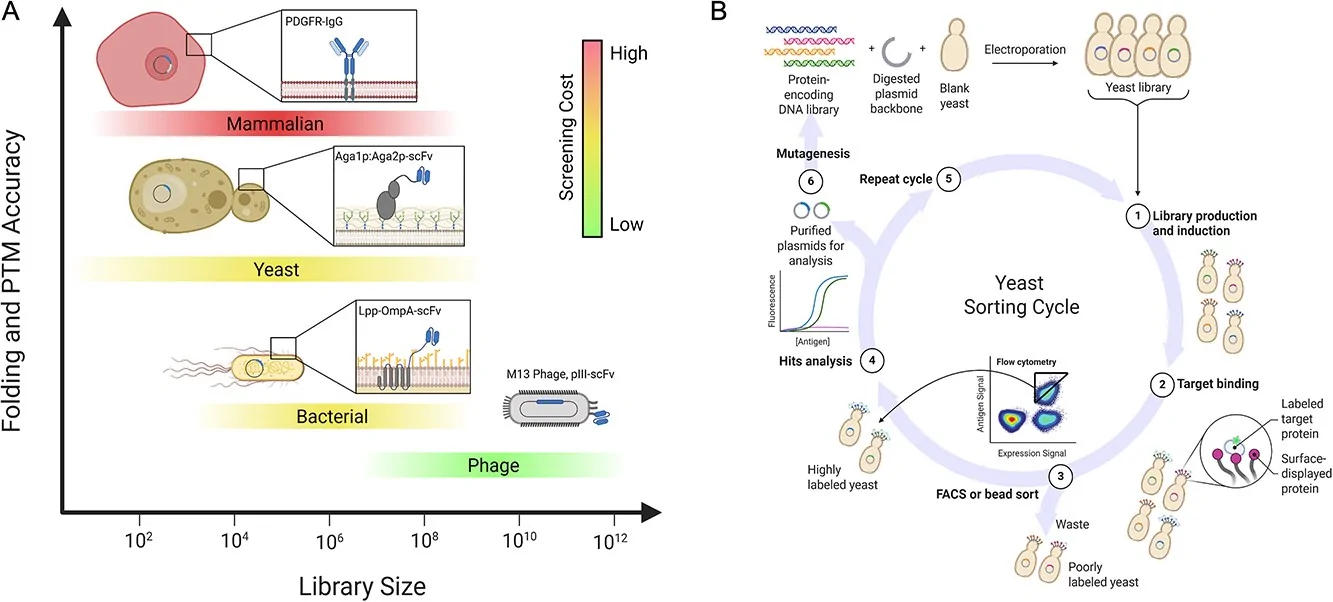
An overview of cell-based display types and a representative selection cycle. (A) Shows examples of the four main cell-based display types plotted on axes defined by library *versus* folding and accuracy of PTM incorporation. Approximate useful library size ranges are illustrated by the colored bars containing the name of the cell (or phage) type. The faded edges of the bars represent the flexible minimum and maximum library sizes possible for each display type. While one could screen lower diversities with phage or bacterial display, most would choose a display technology with better eukaryotic folding and PTM accuracy for these smaller libraries. The colors of the bars correspond to the relative cost for screening the cell-based display technologies, taking into account differences in culture conditions and screening methods. Examples of surface anchoring fusions are illustrated in the zoomed-in boxes for each display type, with an antibody fragment (scFv) or a full-length IgG shown as the protein of interest. (B) this flowchart shows the yeast surface display selection process. DNA inserts encoding mutagenized variants of the protein of interest are mixed with digested plasmid backbone and blank yeast. the mixture is electroporated to transform the DNA into yeast cells, which use homologous recombination to assemble the plasmids. Yeast libraries are grown out and induced to display protein variants anchored on their surface (step 1). Yeast are incubated with labeled target protein (often biotinylated or directly fluorescently labeled) (step 2) and sorted using FACS or magnetic-activated bead sorts (step 3). For the FACS example shown here, unbound or poorly labeled yeast are disposed of, while highly labeled yeast are isolated with a sort gate that selects for high expression-normalized antigen binding (step 3). Highly enriched plasmids can then be extracted for individual phenotype analysis (step 4). The sorted binders are re-induced (step 5) and enriched over several cycles of sorting. Upon library convergence, highly enriched plasmids can be extracted, and mutagenized to begin the cycle again (step 6). The same general process can be followed for the other cell-based display types. Created in BioRender. Porth (2026) https://BioRender.com/rq4aauf.

Each of these protein display platforms has its own niche, informed by maximum library size, available selection techniques, and the fidelity of folding and PTMs for the protein of interest (Fig. 4). Phage display’s large library size (up to 10^12^ members) coupled with inexpensive panning techniques has enabled the isolation of single chain variable fragments (scFvs) that led to at least fourteen FDA-approved monoclonal antibodies and clinical trials for dozens of others (Alfaleh *et al.* 2020). Yeast surface display screening *via* FACS is a popular platform for scFv affinity maturation, as it enables quantitatively informed isolation of improved binders from up to 10^9^-member libraries and it is more likely to fold complex proteins that may have lower stability due to mutations. In addition to antibody development, yeast surface display has been used to evolve complex eukaryotic proteins such as enzymes (Lipovsek *et al.* 2007), receptors (Cochran *et al.* 2004), and disulfide-rich peptides (Silverman *et al.* 2009). Mammalian display libraries are similar in size to yeast; however, they require costly reagents and longer growth periods. Therefore, mammalian display is more frequently employed to do function-first screening and developability testing of high-value proteins, like antibodies (Slavny *et al.* 2024).

The past decade has seen massive improvements in *de novo* protein design accuracy and low-cost DNA production. Researchers now utilize cell-based display to rapidly isolate hits from thousands of computationally designed proteins that bind predetermined epitopes (Liu *et al.* 2025a). Novel cell-based display techniques enable the titration of avidity, screening against complex antigen mixtures, expansion of chemical diversity, continuous evolution, and function-first screening (Lopez-Morales *et al.* 2023; Slavny *et al.* 2024). Together, these innovations promise to accelerate drug development timelines and produce increasingly efficient reagents and diagnostics.

### Consensus protein design (Olivia A. Langlois, Katherine W. Tripp, and Doug Barrick^*^)

**Figure 5:**
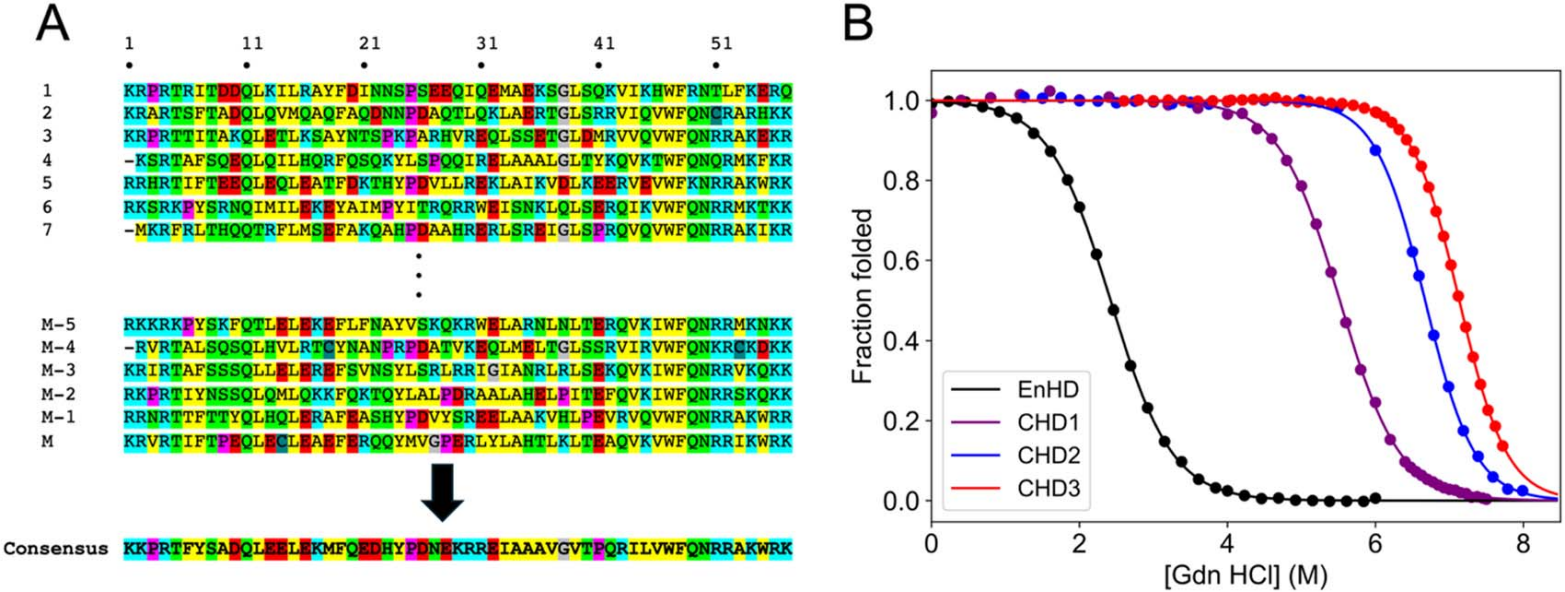
Consensus sequence design. (A) Consensus design begins with curation of a MSA. The frequencies of residues at each position are calculated, and used to make a “consensus sequence” composed of the most common residue at each position. (B) Consensus proteins are often more stable than extant proteins. Here, equilibrium guanidine unfolding curves, monitored by CD spectroscopy, are shown for an extant homeodomain (black), and consensus constructs from a 182 sequence MSA (CHD1, purple), 4552 sequences (CHD2, blue), and 19 221 sequences (CHD3, red). In this example, stability increases with the number of sequences in the MSA.

One simple approach to design stable protein variants from known protein families is “consensus design.” The only input required for consensus design is a multiple sequence alignment (MSA, Fig. 5A). From the MSA, frequencies for the 20 residues at each position are calculated, and substitutions are introduced that replace infrequent residues with the most frequent “consensus” residue. Consensus substitutions can be introduced as single point substitutions, or simultaneously at every position, generating a “consensus sequence.” An early example of single-site consensus engineering involved substitutions to an immunoglobulin domain, where about half of the substitutions resulted in statistically significant stabilization (Steipe *et al.* 1994). An early example of wholesale consensus engineering was the generation of a consensus zinc finger peptide that folds and binds zinc with high affinity (Desjarlais and Berg 1993; Shi *et al*. 1993). The consensus zinc finger study illustrates a potential strength of consensus design—since functional residues are strongly conserved, consensus proteins are likely to retain biological function—indeed, the consensus zinc finger peptides bound cognate DNA sites with high affinity.

Since these early studies, there have been many reports of wholesale consensus designs leading to folded stabilized proteins that retain biological function, with a comprehensive review being published in 2020 (Sternke *et al*. 2020). Nearly all of these reports target monomeric proteins, including globular single-domain proteins and tandem repeat proteins. One motivation for creating consensus tandem repeats proteins is to simplify studies of protein folding. Since all repeats in a tandem consensus array have identical sequences, simple nearest-neighbor models such as a 1D Ising model can be used to quantify cooperativity in folding (Petersen and Barrick 2021). Another motivation for creating consensus repeat proteins is to provide a template for construction of high-affinity binding proteins, with the goal of creating novel diagnostics and therapeutics (Plückthun 2015).

One fairly common outcome of wholesale consensus design is high thermodynamic stability: consensus proteins are often more resistant to thermal or chemical denaturation than extant proteins from the same family (Fig. 5B). Since stability is under selective pressure, mutations that lead to diversity represented in an MSA will favor high stability. To the extent that residues contribute to stability independent of their sequence context, the most frequent substitutions should be stabilizing. However, since other selective pressures contribute to residue frequency, substitution to high-frequency residues at some positions may lower stability but optimize other selected properties such as binding affinity and enzyme activity (Shoichet *et al.* 1995). Indeed, one study of consensus homeodomains has revealed increased affinity toward cognate DNA sequences (Tripp *et al.* 2017), although consensus designed enzymes have been shown to have lower *k*_cat_ values than extant proteins (Sternke *et al*. 2019), suggesting that the assumption of site independence implicit in consensus design may be better suited to stability and binding than catalysis.

Though the consensus approach has generated stable proteins, several issues remain. One relatively unexplored but potentially important feature is MSA depth. Figure 5B shows that for one family of proteins, including more sequences leads to higher stability. Another important feature is diversity. In one recent study, a consensus RNaseH sequence derived from a diverse alignment displayed low stability and cooperativity (Nixon *et al.* 2024); in this case, high stability and cooperativity were obtained by decreasing the diversity of the MSA. This observation is reminiscent of an earlier study of consensus triose phosphate isomerase, where careful curation of the MSA was essential for maintaining structural integrity and activity (Sullivan *et al*. 2011). Improving protocols to optimize MSA depth and diversity would clearly benefit future consensus design efforts.

The continued expansion of sequence databases may provide an opportunity to further increase stability. Another key issue related to consensus design relates to the importance of covariance between pairs of residues. Consensus design makes no attempt to capture pairwise (or higher-order) covariance, although covariance information is believed to be important for protein structure prediction (Morcos *et al.* 2011; Jumper *et al.* 2021). A recent experimental study suggests that including positively covariant residue pairs in protein design does not contribute to stability, though it does seem to be important for enzyme catalysis (Sternke *et al.* 2026). By identifying key residue pairs that contribute to catalysis and combining with consensus information, sequences may be identified that maximize both stability and activity. By comparing consensus- and covariance-based design strategies to ML-based design methods (see Section Computational methods), it may be possible to better understand why both methods succeed (and sometimes fail).

### Ancestral sequence reconstruction (Gabriel Foley and Elizabeth M.J. Gillam^*^)

**Figure 6:**
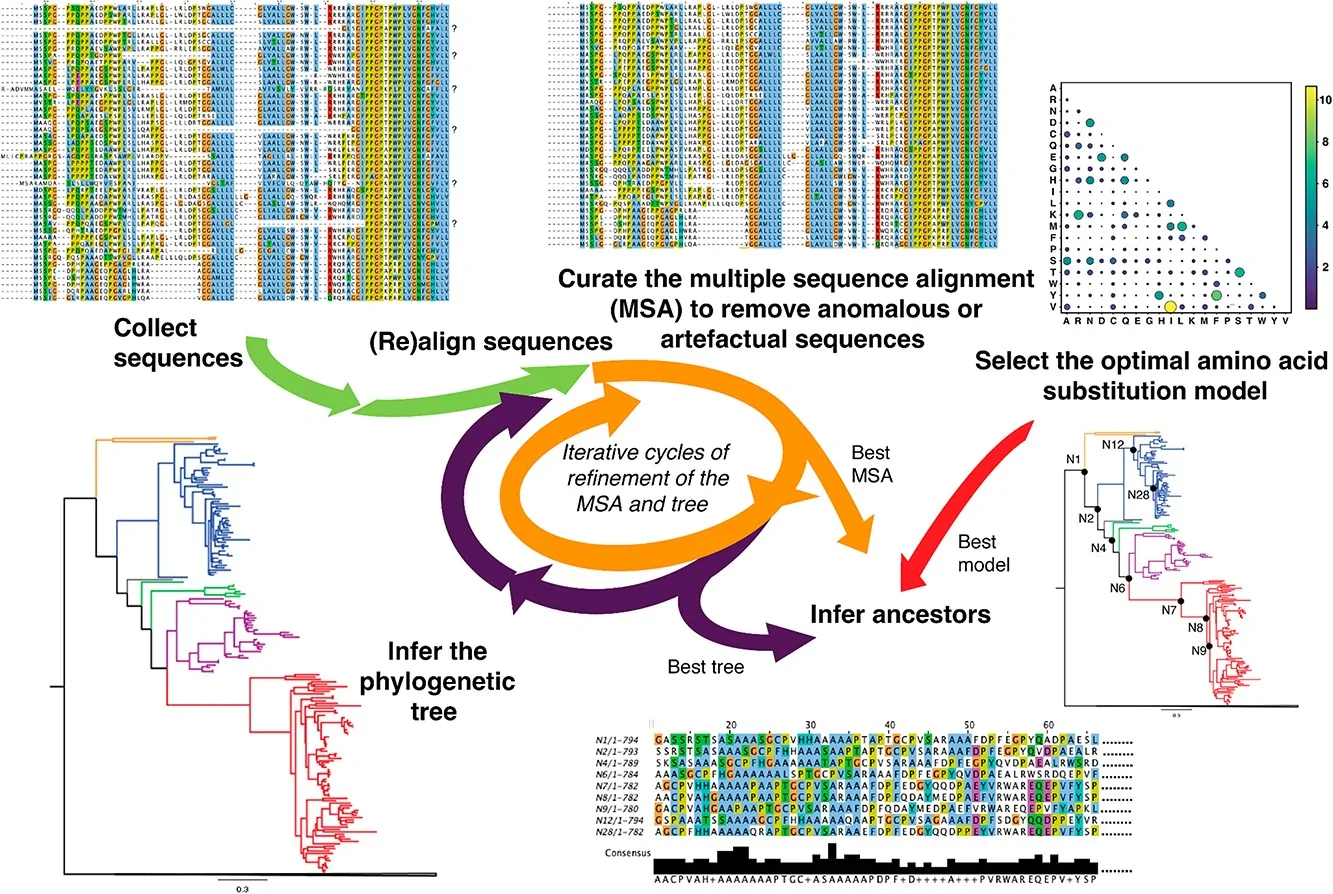
Workflow for ASR. Extant sequences are collected from as diverse a range of sources as possible to reflect the full sequence diversity of a protein family, then aligned to generate a MSA. Sequences containing probable artefacts that would obviate expression of a functional protein (e.g. pseudogenes, frameshifts, insertions and deletions representing introns and missed exons) are removed from the alignment using iterative cycles of inspection and realignment, then the curated alignment is used to infer a phylogenetic tree. The tree is similarly refined in an iterative fashion until it reflects a plausible evolutionary history of the protein family, consistent with the expected species tree. The model of amino acid substitution that best describes the evolutionary process within the protein family is identified, then the final MSA, phylogenetic tree, and evolutionary model are used to infer the most likely ancestral sequences at internal nodes of the tree.

Ancestral sequence reconstruction (ASR) leverages natural evolution to focus engineering efforts within functionally and structurally viable regions of sequence space. Sequences from the extant representatives of a protein family are aligned, curated, and used to infer an evolutionary tree (Fig. 6). Then the tree, sequence alignment and an evolutionary model are combined to infer the ancestral state at each branchpoint of the tree. The tree and ancestors are both inferred using probabilistic methods, typically maximum likelihood or Bayesian approaches. Since by definition, evolutionary intermediates have already been subject to natural selection, an ASR that faithfully reflects the natural evolution of a protein family should provide a set of related, structurally intact proteins that are functional in at least some physiological context.

ASR was first used to explore the evolution of protein families (Bridgham *et al*. 2006; Boucher *et al.* 2014). However, it was soon discovered that ancestors were often (but not always! (Skovgaard *et al.* 2006; Jones *et al.* 2024)) more thermostable than the corresponding extant forms from which they were inferred, sometimes by as much as 20–30°C when comparing T_50_ values (Trudeau *et al*. 2016; Gumulya *et al.* 2018). This was not unexpected for proteins from Precambrian times, which may have originated in thermophilic organisms (Gaucher *et al.* 2003; Akanuma *et al.* 2015), but the biological importance of the observed thermostability is debated for proteins inferred from mesophiles (Trudeau *et al*. 2016; Gumulya *et al.* 2018). An explanation for the generally higher stability of inferred ancestors compared to extant forms is that maximum likelihood methods, by choosing the most probable residue at each site independently, tend to avoid residues that are destabilizing since they are less frequent (Williams *et al.* 2006). The degree of stabilization varies across studies, with stability landscapes often showing apparently stochastic gains and losses, likely reflecting variation in the relative contributions of artifactual bias and genuine biological signal (Hart *et al.* 2014; Wheeler *et al.* 2016; Arenas *et al.* 2017; Chisholm *et al.* 2024; Thomson *et al.* 2026). Alternatively, since thermostability confers greater tolerance to mutations, it may be that the large protein families that are typically the focus of ASR experiments are biased toward those that have been able to diverge most extensively, i.e. from a stable ancestor (Thomson *et al.* 2026). Ultimately, questions about the evolutionary relevance of increased thermostability are less important when the purpose of the ASR is simply protein engineering than when ASR is used to study protein evolution.

Since ASR can lead to larger increases in thermostability that traditional random or combinatorial mutagenesis approaches, it has become a method of choice for stabilizing proteins, either by integrating ancestral residues into directed evolution strategies (Cox and Gaucher 2014; Gumulya *et al.* 2019) or using ancestors as robust templates for further engineering. The principal aim of most engineering campaigns using ASR is to obtain a more structurally stable representative of a given protein family, that retains at least some of the functional properties of the extant forms. Stability facilitates structural characterization and elucidation of structure–function relationships (Chiang *et al.* 2023). However, ASR can also generate useful functional diversity for protein engineering; small libraries of evolutionary intermediates often show diverse characteristics (Babkova *et al.* 2017; Gumulya *et al.* 2018; Hernández-Meléndez *et al.* 2025). It has been assumed that ancestors should be more promiscuous than the extant forms, but while this may be true for certain protein families, it is not borne out by the evidence from ASR experiments on other proteins (Wheeler and Harms 2021; Thomson *et al.* 2026), including common targets of engineering for industrial application (Gumulya *et al.* 2018; Livada *et al.* 2023). Beyond stability and functional diversity, ASR identifies sequences that occupy regions of sequence space where epistatic interactions have already been resolved by natural selection, making them tractable starting points for additional mutational exploration (Harms and Thornton 2010). ASR has also been used to understand how interfaces between proteins become fixed over evolutionary time in ways that directly inform the further engineering of protein complexes (Hochberg *et al.* 2020), and sets of ancestral sequences make strong candidates for engineering libraries, having been filtered through natural selection to remove unfit variants (Cole and Gaucher 2011).

Efforts have been made to automate the ASR process with mixed success. A key issue is sequence curation (Thomson *et al*. 2022): sequence databases are full of erroneous sequences (e.g. due to miscalled start and stop sites, intron/exon boundaries) which, if included, will compromise the inference of a functional ancestral protein. High quality alignments that provide strong phylogenetic signals from dense sampling of the available extant sequences are critical for maximizing accuracy in ASR (Muñiz-Trejo *et al.* 2025). Unfortunately, it is often challenging to discern whether an “atypical” sequence represents true biological variation or an artefact of the conversion of genome sequence data to open reading frames and amino acid sequences. Thus, manual oversight is essential in curating sequences (Thomson *et al.* 2022), and a knowledge of the structure–function relationships of the protein family is important in deciding, e.g. what sequence motifs are essential, and which indels could be tolerated without compromising protein function. A second issue is the reliance on two-dimensional MSAs (Vialle *et al*. 2018), where arbitrary decisions are made about which residues in variable regions of one protein match those in another protein, whereas the regions concerned may have quite different evolutionary origins, and show no meaningful homology.

As in many other areas of protein engineering, AI tools may help with both sequence curation and alignment challenges in the future. Developments are also likely in the modeling of evolutionary processes and the integration of structural information, whether experimental or predicted (e.g. by AlphaFold), since structure tends to be conserved more than sequence.

### Protein ligation (Luis F. Guerra and Manuel M. Müller^*^)

**Figure 7:**
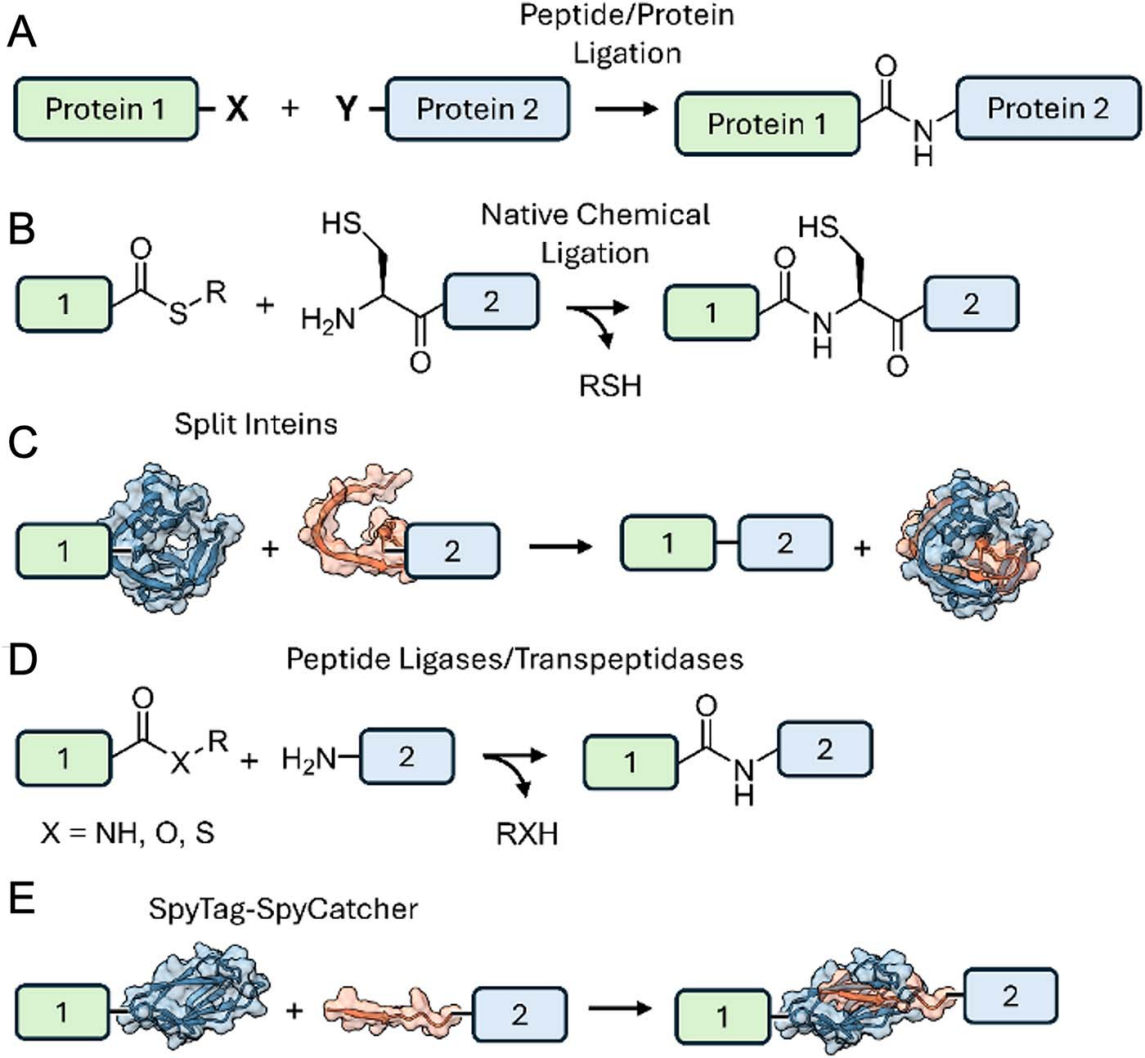
Schematic overview of protein ligation technologies. Protein ligation (A) can be accomplished *via* native chemical ligation (B), split inteins (C), peptide ligases and transpeptidases (e.g. sortases, subtiligases and butelases as well as their engineered variants) (D), and the SpyTag-SpyCatcher system (E).

Protein and peptide ligations provide avenues for protein engineering beyond traditional genetic constraints. These reactions entail the covalent coupling of two or more peptides or protein fragments to yield contiguous proteins with fully customizable segments (Fig. 7A). A versatile toolkit has been developed for post-translationally “cutting and pasting” protein sequences, spanning purely chemical methods (see Section Chemically synthesized proteins (Guo-Chao Chu and Lei Liu^*^)), protein-mediated ligation reactions, and combinations thereof. In this way, proteins can be furnished with non-natural sequences, new topologies, and artificial prostheses which range in size from single amino acids to synthetic polymers. Example functional augmentations of proteins include spectroscopic and mechanistic probes or controllers such as natural PTMs and artificial photoswitches. Accordingly, these ligation technologies have diverse applications in fundamental research, synthetic biology, and materials science, explored in detail in other sections of this status report.

Native chemical ligation (Dawson *et al.* 1994), the condensation between polypeptides containing a C-terminal α-thioester and an N-terminal cysteine (Fig. 7B), embodies a foundational technology in this field. Since its inception, many variations with distinct chemoselectivities (Pattabiraman and Bode 2011; Agouridas *et al.* 2019) have enabled the synthesis of bespoke proteins carrying non-standard and entirely new-to-nature building blocks. A particularly useful feature of native chemical ligation is that fragments with the necessary reactive termini can be produced by either chemical synthesis (Section Chemically synthesized proteins (Guo-Chao Chu and Lei Liu^*^)) or recombinant expression, allowing researchers to mix-and-match for the semi-synthesis of proteins featuring both synthetic and recombinant segments. Recombinant proteins with N-terminal cysteines can be obtained through selective proteolysis, while C-terminal thioesters can be introduced using inteins (Muir *et al*. 1998)—*int*ervening prot*ein*s that use a thioester mechanism to splice themselves out of a protein precursor with concomitant ligation of the flanking sequences (Perler 2002; Shah and Muir 2014).

Inteins that are naturally or artificially split and spontaneously self-assemble can also be harnessed directly for protein ligation (Fig. 7C) (Mills *et al.* 1998; Southworth 1998). Early applications of this trans-splicing reaction included the segmental isotopic labeling of proteins (Yamazaki *et al.* 1998). Other protein-based ligation technologies (Pihl *et al*. 2023) include natural and engineered peptide ligases and transpeptidases, e.g. sortases (Mazmanian *et al.* 1999; Mao *et al.* 2004), subtiligases (Jackson *et al.* 1994) and butelases (Nguyen *et al.* 2015) (Fig. 7D), as well as systems that form covalent isopeptide bonds *via* amino acid side chains, exemplified by SpyTag-SpyCatcher (Zakeri *et al.* 2012) (Fig. 7E). These proteins themselves have been subjected to extensive engineering campaigns to enhance their activity and tune their sequence specificity, yielding a toolkit for reliable biological “click” reactions (Howarth 2025).

Linking of intact proteins or domains and furnishing proteins with customized termini is nowadays routine, although new applications often require optimization, including the consideration of ligation “scars” (defined as remnants from amino acids/peptide sequences which are required for efficient ligation but are not native to the protein product). Building on these terminal-modification strategies, recent engineering efforts with coordinated peptide “cut-and-paste” reactions facilitate editing of internal regions of proteins (Beyer *et al.* 2025; Hua *et al.* 2025). Remarkably, these processes could be implemented in living cells, enabling the installation of artificial units into diverse cellular proteins. Protein ligation technologies can also operate on cellular surfaces (Kofoed *et al.* 2025) and in living organisms (Wu *et al*. 2017a) with applications in gene therapy (Villiger *et al.* 2018; Tornabene *et al.* 2019; Padula *et al.* 2022; Tasfaout *et al.* 2024; Ferla *et al.* 2025).

Nevertheless, protein ligation in living systems represents a frontier, where production or delivery of protein fragments, control of chemical kinetics and equilibria as well as analysis of products and the fate of side products are considerably more challenging than in *in vitro* systems.

### Automation and robotics (Nilmani Singh and Huimin Zhao^*^)

Automation and robotics in protein engineering emerged from the broader field of laboratory automation and the rise of high-throughput screening platforms in the 1990s, when robotic liquid handlers enabled testing of large compound libraries for drug discovery (Comley 2002; Chapman 2003; Pereira and Williams 2007). These technologies were soon adapted toward the design-build-test-learn (DBTL) cycle of protein engineering. Automation in protein engineering aims to accelerate the iterative process through robotics-driven miniaturized, parallelized assays that enable high-throughput measurement of variant fitness (Chao *et al.* 2017; Yu *et al.* 2023) with minimal human involvement. Incorporating robotics enables researchers to standardize experiments, increase reproducibility, reduce labor, and costs, and accelerate discovery timelines. We envision an autonomous protein engineering platform that can design and execute end-to-end DBTL cycles, dramatically expanding the ability to explore protein landscapes and development of protein-based products (Carbonell *et al*. 2019; Yang *et al*. 2024).

**Figure 8:**
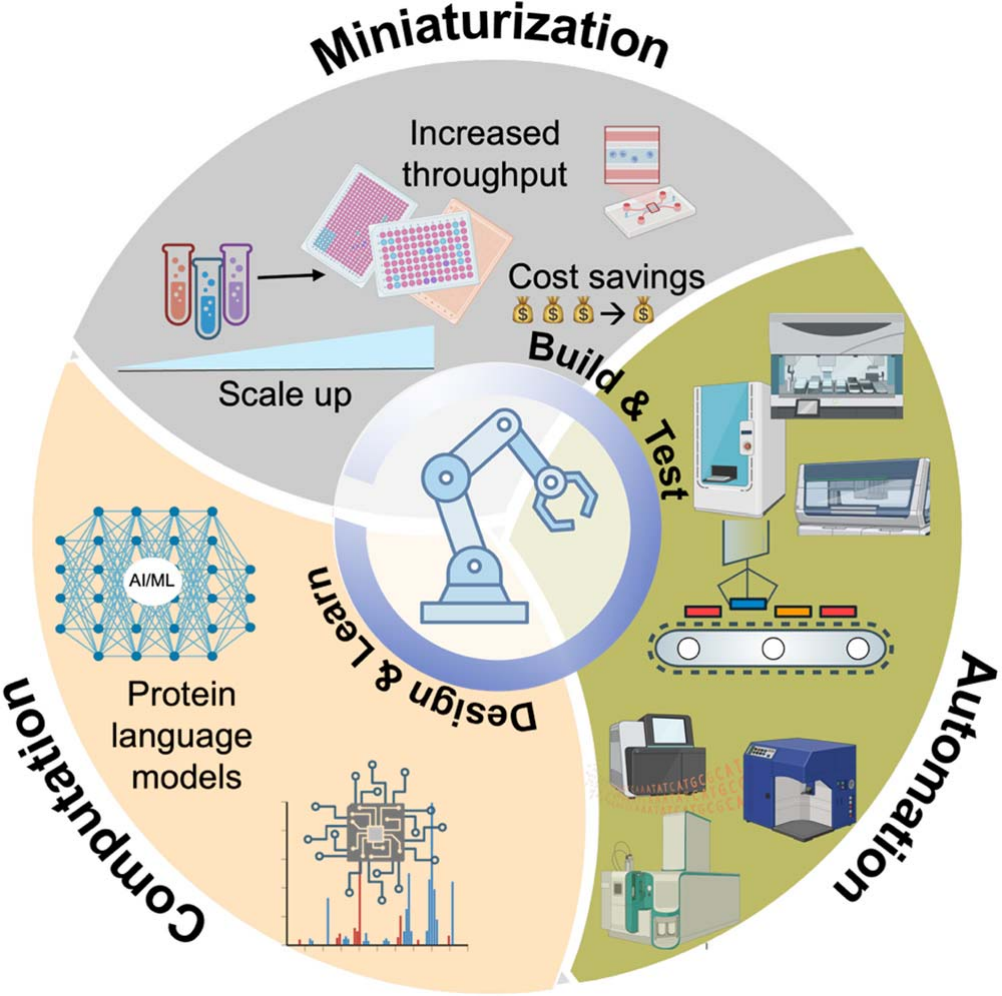
Automation and robotics for protein engineering. The major driving forces behind the progressive adoption of robotics in protein engineering. Miniaturization enables screening of protein variants in small volumes, dramatically increasing throughput while reducing reagent costs. Robotics enables automated workflows for iterative DBTL cycles with minimal human intervention, providing continuous operation, standardized experiments, reduced errors, and high-throughput capabilities. AI/ML algorithms and computational models guide protein design, predict fitness landscapes, analyze high-dimensional datasets, and optimize experimental parameters for iterative improvement. Together, these driving factors create synergistic capabilities that revolutionize protein engineering, enabling accelerated discovery of novel protein designs. Created in BioRender. Zhao (2025) https://BioRender.com/88yomuf.

Modern robotic protein engineering platforms have achieved remarkable sophistication, with some systems capable of analyzing tens of thousands of samples per day. Reliable high-throughput liquid handlers and advanced analytical equipment have been central to this progress. Many commercial vendors offer laboratory automation platforms that integrate liquid handling robots, peripherals, and analytical instruments. These platforms have greatly accelerated both build and test phases of protein engineering. Automated DNA assembly systems (Ma *et al.* 2024) such as PlasmidMaker (Enghiad *et al.* 2022) speed up the build phase, while large liquid handlers streamline sample preparation for fitness measurement with exceptional speed and efficiency. High-throughput analytical experiments including next-generation sequencing, mass spectrometry (Simon *et al.* 2021), and plate-reader based assays are now routine across laboratories. More recently, integration of AI with biofoundry platforms, integrated robotic infrastructure for automated experimentation, has demonstrated autonomous protein engineering capabilities requiring minimal human intervention (Rapp *et al*. 2024; Singh *et al.* 2025b; Zhang *et al.* 2025c). Emerging technologies such as cell-free systems (Landwehr *et al.* 2025) and microfluidic devices for ultrahigh-throughput screening (Gantz *et al.* 2023) promise to further compress DBTL timelines and expand the accessible protein landscape. Robotics-enabled laboratories are transforming protein engineering from a manual, low-throughput endeavor into a scalable, data-driven discipline capable of systematically exploring vast protein sequence spaces (Fig. 8).

The integration of robotics and AI in protein engineering is pushing the field toward autonomous experimentation capable of executing DBTL cycles with minimal human oversight, where ML algorithms drive experimental design, data analysis, and iterative optimization (Chen *et al.* 2025a; Singh *et al.* 2025a). Advances in protein language models (Bjerregaard *et al.* 2025) now enable efficient exploration of vast sequence landscapes, and their incorporation into robotic workflows (Singh *et al.* 2025b; Zhang *et al.* 2025c) has further enabled accelerated iterative cycles. While robotic experimentation for protein engineering offers tremendous potential, establishing these facilities requires substantial investment and creates high entry barriers due to complex technical, infrastructure, and expertise requirements. Industry and central funding agencies are increasingly investing in cloud-based robotic laboratories that combine advanced experimental instruments with state-of-the-art AI tools to streamline design and execution. These cloud laboratories have the potential to democratize access to advanced protein engineering infrastructure, lowering barriers for researchers’ worldwide and accelerating discovery of novel protein designs with broad societal impact.

## Computational methods

### *De novo* design of new protein folds (Rie Tatsumi-Koga and Nobuyasu Koga^*^)

**Figure 9:**
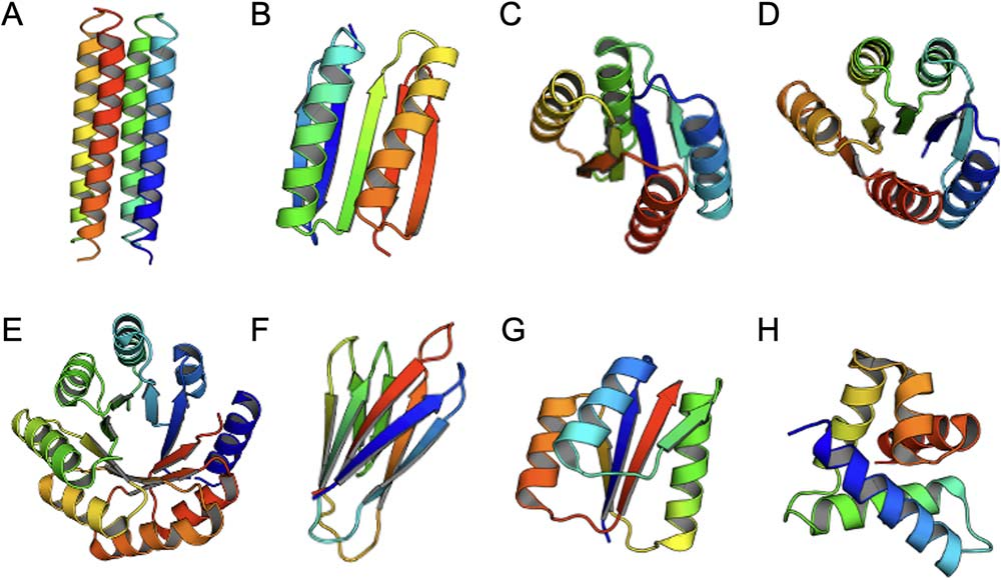
Representative *de novo* designed proteins. (A) All-α protein with a right-handed coiled coil structure (Harbury *et al.* 1998). (B) αβ-protein: Top7 (Kuhlman *et al.* 2003). (C) αβ-protein: Rossmann 2x2-fold protein (Koga *et al.* 2012). (D) αβ-protein: Rossmann 2x3-fold protein (Koga *et al.* 2021). (E) TIM-barrel structure (Huang *et al.* 2016b). (F) All-β protein (Marcos *et al.* 2018). (G) αβ-protein: knot-forming novel protein (Minami *et al.* 2023). (H) Complex all-α protein (Sakuma *et al.* 2024).

*De novo* protein design addresses a daunting search problem: from the 20*^N^* possibilities for a length-*N* sequence (when *N* = 100, 20*^N^* = ~10^130^), identify those that fold into a single, functional structure. Life has explored only a tiny fraction of the 20*N* sequence space over billions of years of evolution (Baker 2019), and the proteins we observe today are merely those that have been selected under Earth’s environmental conditions. However, from the perspective of human applications—industrial, medical, or involving synthetic molecules—vast numbers of functional proteins likely remain undiscovered in the unexplored regions of sequence space. In the late 1980s, pioneering efforts explored four-helix bundle protein structures using sequence features such as hydrophobic/hydrophilic patterning and geometric rules (DeGrado *et al*. 1987; Hecht *et al.* 1990). However, the designs from this era typically populated molten-globule ensembles rather than achieving a unique native state. This situation began to change as the accumulation of experimental structures expanded our knowledge and computational methods developed. In 1997, Mayo and colleagues employed rotamers—discrete representations of side-chain conformational states—to computationally search for side-chain conformations that minimize the energy of protein structures, successfully achieving complete redesign of the side-chain structure of a zinc finger motif (Dahiyat and Mayo 1997). Furthermore, Kim and colleagues successfully achieved computational design of four-helix bundle structures from the backbone by leveraging Crick’s parametric equations for coiled coils to construct the helix bundles and employing rotamers to design the side-chain structures (Harbury *et al.* 1998) (Fig. 9A). In 2003, Baker and colleagues achieved landmark success with their developed Rosetta software. By assembling fragments from naturally occurring protein structures and combining this with rotamer-based design, they created Top7 with a novel fold containing both α-helices and β-strands, going beyond simple helical bundle designs (Kuhlman *et al.* 2003) (Fig. 9B).

Building on these successes, Koga et al. developed physicochemically grounded design principles, with backbone geometry rules that ensure consistency between local and non-local interactions, which made possible the encoding of designed proteins to have a funnel-shaped energy landscape toward a unique folded state (Koga *et al.* 2012, 2021) (Fig. 9C and D). These principles have since been extended to design diverse protein structures including novel folds (Huang *et al.* 2016b; Marcos *et al.* 2018; Minami *et al.* 2023; Sakuma *et al.* 2024) (Fig. 9E–H). Concurrently, the search expanded beyond well-folded structures to encompass functional design. Repurposing naturally occurring scaffolds *via* rotamer-based approaches, researchers created an influenza hemagglutinin binder (Fleishman *et al.* 2011), an enzyme for an abiological Diels–Alder reaction (Siegel *et al.* 2010), and self-assembling nanomaterials such as two-component icosahedral cages (Bale *et al.* 2016). Notably, functions were installed directly on *de novo* designed backbones—e.g. compact miniproteins that neutralize SARS-CoV-2 at picomolar potency (Cao *et al.* 2020), and fluorogenic compound binding proteins (Dou *et al.* 2018).

The advent of deep learning in recent years has fundamentally changed *de novo* protein design. The deep learning-based protein design approach enables rapid generation of diverse proteins that were computationally intractable only a few years ago. RFdiffusion generates various protein backbones with functional constraints (Watson *et al.* 2023). For sequence generation, ProteinMPNN provides robust sequence design given backbone structures (Dauparas *et al.* 2022). A common workflow that couples these tools—e.g. RFdiffusion for backbone generation, ProteinMPNN for sequence design, AlphaFold for structure validation (Jumper *et al.* 2021)—accelerates design of functional proteins (Lauko *et al.* 2025; Kim *et al.* 2026). Looking forward, the field aspires to design proteins that operate within cellular networks, as well as highly sophisticated proteins such as molecular motors. Guided by physical principles and empowered by AI, we have transformed the once-daunting search through 20*^N^* possibilities into an increasingly tractable challenge, enabling the creation of proteins that nature never made.

### Computational design of protein binders (Tiger S. Zhang and Brian Kuhlman^*^)

**Figure 10:**
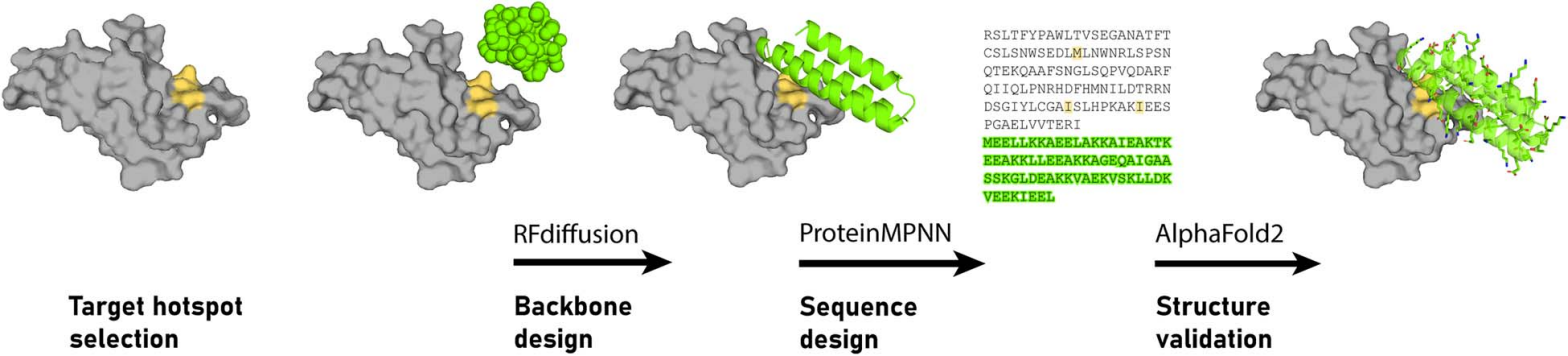
Overview of the strategy for computational design of protein binders. Computational binder design generally follows the steps of target hotspot selection, backbone design, sequence design, and structure validation. First, a structural model of the target is obtained, and hotspot residues for the binder interface are selected. In backbone design, methods such as RFdiffusion are used to generate backbones which interact with the hotspot residues. Next, in sequence design, sequences that are likely to fold into the designed backbones are generated *via* methods such as ProteinMPNN. Finally, the structure is validated by refolding the designed binder and target sequences through AlphaFold2 or other structure prediction models. High-quality binder sequences can be selected by filtering on AlphaFold2 confidence metrics.

The computational design of binders that interact with high affinity and specificity to a target protein has been an important goal in protein engineering. Early methods, such as the Rosetta software suite, modeled interactions between a potential design and its target with physics-based functions and scored designs based on their interface energies, among other metrics (Alford *et al.* 2017; Cao *et al.* 2022). More recently, deep learning models have revolutionized protein binder design (Ingraham *et al.* 2019; Gao *et al.* 2020; Jumper *et al.* 2021; Dauparas *et al.* 2022). Structure prediction algorithms, such as AlphaFold2 (Jumper *et al.* 2021), can predict interactions between protein chains, while sequence design algorithms, such as ProteinMPNN (Ingraham *et al.* 2019; Dauparas *et al.* 2022), can propose sequences that are likely to fold into a given protein backbone (Fig. 10).

A primary challenge in binder design is finding realistic backbones with high shape-complementary to a target protein (Cao *et al.* 2022). Enumerative docking and interaction fingerprint methods, such as RifDock and MaSIF, have been successful in designing binders to a variety of targets (Cao *et al.* 2022; Gainza *et al.* 2023; Huang *et al.* 2024; Lv *et al.* 2024; Ragotte *et al.* 2025; Yang *et al.* 2025a). Alternatively, denoising diffusion models, such as RFdiffusion (Watson *et al.* 2023), Chroma (Ingraham *et al.* 2023), and ProteinGenerator (Lisanza *et al.* 2025) can generate backbones that mold to a target structure. RFdiffusion can also be conditioned on target hotspot residues or user-defined geometries, making it especially powerful (Watson *et al.* 2023). Sequences for the backbones can be designed *via* ProteinMPNN and evaluated through AlphaFold2 (Bennett *et al.* 2023; Watson *et al.* 2023). Notably, the backbones generated by RFdiffusion have had much higher experimental success rates than those designed by older methods (Bennett *et al.* 2023). RFdiffusion has been used to generate binders to a variety of targets including microbial proteins (Watson *et al.* 2023; Fox *et al.* 2025a), cell surface receptors (Watson *et al.* 2023; Glögl *et al.* 2024; Householder *et al.* 2025; Johansen *et al.* 2025; Liu *et al.* 2025a), snake venoms (Vázquez Torres *et al.* 2025), and peptides/intrinsically disordered regions (Vázquez Torres *et al.* 2024; Liu *et al.* 2025d; Wu *et al.* 2025). Initial hit binders can be optimized in RFdiffusion through partial diffusion, generating structural diversity around a particular fold which can also improve the binding affinity (Watson *et al.* 2023; Glögl *et al.* 2024; Vázquez Torres *et al.* 2024; Liu *et al.* 2025d; Wu *et al.* 2025). A limitation of RFdiffusion is that it mainly generates helical binders. However, variants of RFdiffusion are capable of generating designs which interact with their targets *via* beta strand-pairing or antibody-like loops (Bennett *et al.* 2026; Sappington *et al.* 2026).

Other design methods iterate between binder sequence and structure to design high-affinity binders without experimental optimization (Goudy *et al.* 2023; Pacesa *et al.* 2025). Similarly to directed evolution methods and other genetic algorithms (Jendrusch *et al.* 2025), EvoPro evolves binders *in silico* through rounds of sequence diversification, AlphaFold2 structure prediction, and scoring with AlphaFold2 confidence metrics (Goudy *et al.* 2023). BindCraft is another binder design pipeline which leverages the information learned by deep learning algorithms (Anishchenko *et al.* 2021; Goverde *et al.* 2023; Pacesa *et al.* 2025). In a BindCraft trajectory, the binder sequence is backpropagated through AlphaFold2, and the resulting gradients are used to iteratively improve the design (Pacesa *et al.* 2025). In contrast to fixed-backbone methods, EvoPro and BindCraft re-predict the design-target structure at each iteration, enabling backbone flexibility that may improve binder generation (Goudy *et al.* 2023; Pacesa *et al.* 2025). However, deep learning-based confidence metrics are imperfect measures of experimental success and do not correlate with binding affinity (Bennett *et al.* 2023; Fox *et al.* 2025b). In addition, new methods are needed for the design of protein binders against challenging targets, such as polar interfaces and flexible protein regions (Fox *et al.* 2025b).

### Prediction of protein complex structures (Michael Chungyoun and Jeffrey J. Gray^*^)

The prediction of protein complexes is the task of assembling two separate protein components into their biologically relevant complex structure (Gray *et al.* 2003). Protein–protein interactions mediate cellular phenomena that allow living systems to function. Although not yet solved, advancements in this domain pave the way for understanding the energetics of macromolecular function and ultimately the complex essence of life (Gray *et al.* 2003; Kaczor 2024). The first computational approach for predicting protein–protein interactions replaced each residue with a single interaction center (Wodak and Janin 1978), but advancements in deep learning have since enabled docking prediction tools (Christoffer *et al.* 2021; Jones *et al.* 2022; Liu *et al*. 2025b) that dominate today’s Critical Assessment of PRedicted Interactions (CAPRI) challenges (www.capri-docking.org/assessment/). The breadth of models available has increased yet all are developed according to the guiding concepts introduced by Wodak and Janin (Wodak and Janin 1978): a robust protein–protein predictive model requires a sampling strategy to create viable decoys and a scoring function to identify the single native pose.

The main categories of current sampling algorithms include homology modeling (Bitencourt-Ferreira and de Azevedo 2019), template-based modeling (Singh *et al.* 2023), exhaustive search with fast Fourier transform (Xu *et al.* 2017), local shape feature matching (Christoffer *et al.* 2021), randomized search (Gray *et al.* 2003), and data-driven docking(Jumper *et al.* 2021). Computationally efficient sampling algorithms assume a rigid (Nguyen *et al.* 2020) protein conformation, while others account for the flexibility (Krupa *et al.* 2021) present in real interacting protein systems. Several scoring function approaches are utilized including force-fields (Moal and Bates 2010), statistical (Dhawanjewar *et al*. 2020) or empirical (Gray *et al.* 2003; Su *et al.* 2014) approximations, and ML (Jung *et al.* 2023). AlphaFold2 integrated both steps into a single co-folding (Morehead *et al.* 2025) pipeline. The release of AlphaFold2 enabled a five-fold rise in high-quality CAPRI models compared to 2 years prior (Lensink *et al.* 2023), with prediction speeds that allow the investigation of all protein–protein interactions across the entire human proteome of 200 million protein pairs (Zhang *et al.* 2025a)—a previously intractable challenge.

**Figure 11:**
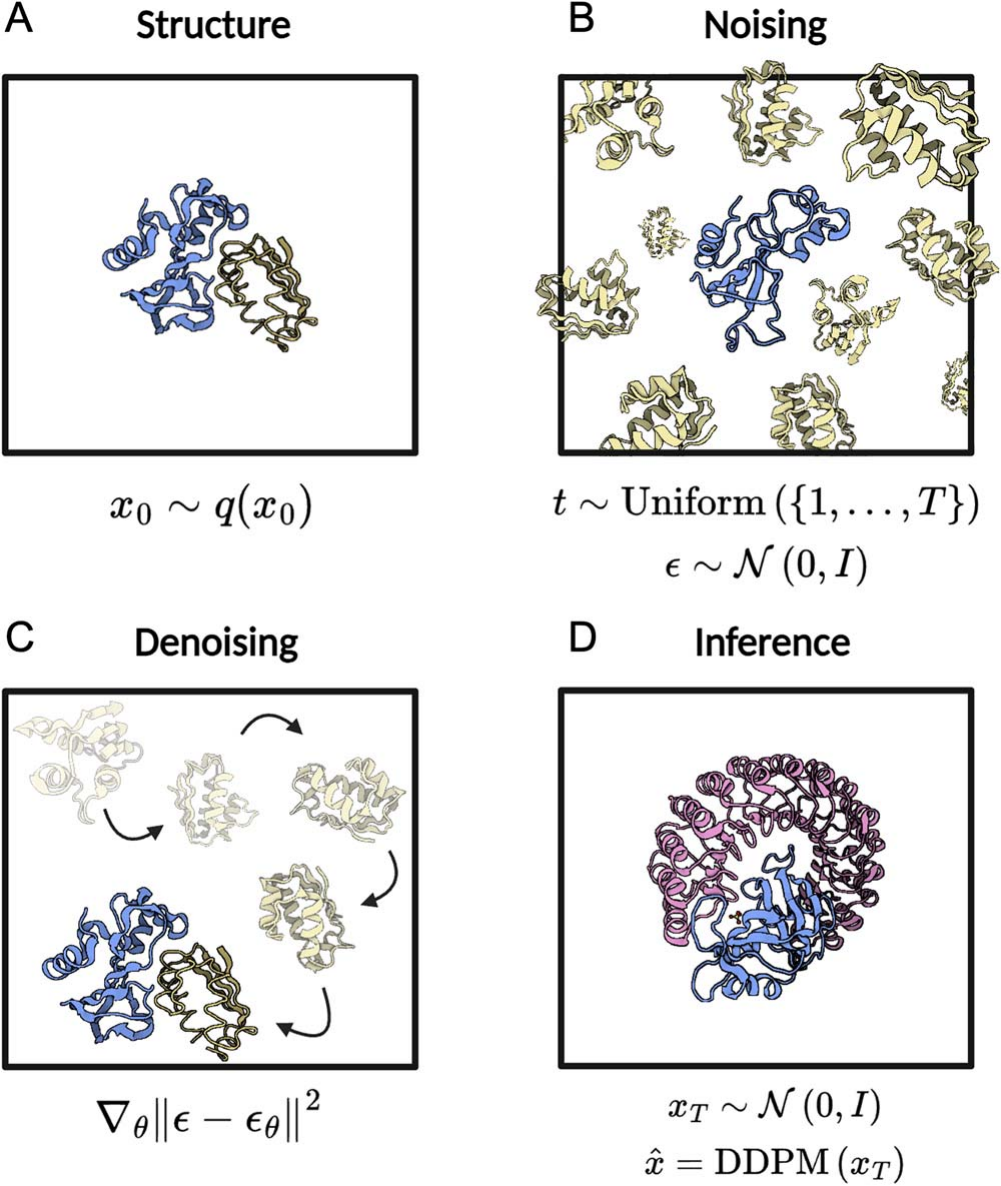
Generative denoising diffusion probabilistic models for docking prediction. Diffusion models are the latest class of deep neural networks that have significantly improved the capabilities of protein docking and scoring strategies. DiffDock (Corso *et al.* 2022) first incorporated a diffusion model, with components later utilized in AlphaFold3 (Abramson *et al.* 2024) and DFMDock (Chu *et al.* 2024). Training a docking diffusion model involves first (A) sampling a structure, 𝑥_0_, from the distribution of known protein–protein structures, *q*. In this case, 𝑥_0_ is barnase bound to barstar (PDB ID 1BRS) (Buckle *et al*. 1994). Then, (B) the ligand barstar (right-hand side protein structure) is “noised” by iteratively translating and rotating it away from the receptor barnase (left-hand side protein structure) at different times, *t*, with noise, *ϵ*, sampled from a Gaussian distribution. The final step of training involves (C) the model learning to dock the structures together from various noised coordinates, adjusting the model weights to match the model-predicted noise, *ϵ*_θ_, with the true noise, *ϵ*. once trained, (D) the model can be used to predict unknown docked structures, such as this RNase Sa complex with its inhibitor (PDB ID 1DFJ) (Kobe and Deisenhofer 1995).

Today’s best predictors at CAPRI57, including AlphaFold3 (Abramson *et al.* 2024), find good models for 75% of targets (www.capri-docking.org/assessment/)—an incredible feat—yet several challenges remain. Today’s best docking prediction models only have a 50% accuracy for antibody–antigen complexes (Hitawala and Gray 2025), likely due to the lack of appropriate MSAs and diverse, loop-mediated interfaces (Fromm *et al*. 2026). Docking membrane proteins remains challenging due to their hydrophobic nature, poor expression, low abundance, and crystallization difficulties (Kaczor 2024). Current docking models are trained predominantly on crystallographic structures in the Protein Data Bank (Berman 2000), which over-represent stable, soluble complexes and provide limited coverage of intrinsically disordered interfaces (Erdős *et al.* 2017), transient interactions, and PTMs (Schofield *et al.* 2024). Compounding this, crystallographic structures are static snapshots that do not capture the solution-state behavior of proteins: Temperature, pH, and ionic concentration modulate binding interfaces and interaction specificity in ways that data-driven approaches do not account for. Binary binding prediction, the task of predicting whether two proteins interact at all, remains poorly solved, with current AI models showing limited accuracy across broad protein families (Wohlwend *et al.* 2025). An emerging trend in co-folding and docking models is the incorporation of diffusion models (Abramson *et al.* 2024; Discovery *et al.* 2024; Wohlwend *et al.* 2025) (Fig. 11) to sample multiple poses. While protein AI models continue to improve, classical approaches remain indispensable for interpretability: Physics-based energy functions allow decomposition of a predicted score into mechanistically identifiable terms (e.g. electrostatics, solvation, van der Waals), providing insight that purely data-driven models do not yet offer. Four unsolved problems stand out as priorities for the next decade: Accurately modeling conformational change upon binding; reliably predicting binding affinities; generalizing robustly across the full diversity of biologically relevant complexes; and developing integrated physics-based and data-driven frameworks capable of overcoming the scarcity of experimentally characterized binding data. A promising direction is the integration of physics with AI (Chu *et al*. 2024; Harmalkar *et al*. 2025), bridging decades of progress in physics-based modeling with the modern, orthogonal advances of deep learning to solve the remaining challenges in computational protein docking.

### Computational enzyme design (Benjamin J. Smith and Roberto A. Chica^*^)

*De novo* enzyme design, which aims to create biocatalysts from first principles to catalyze specific reactions, emerged in the early 2000s following advances in computational protein design (Bolon and Mayo 2001). Early methods relied on quantum mechanical calculations to define the optimal spatial arrangement of catalytic residues around a model transition state, or theozyme, which was then grafted into a pre-existing protein scaffold. The central challenge lay in engineering both the catalytic machinery and a substrate-binding pocket capable of stabilizing that transition state with high precision. In practice, natural protein scaffolds were repurposed to host these artificial active sites, and computational algorithms searched for amino acid configurations that best stabilized the theozyme geometry. This structure-based strategy established the conceptual foundation of *de novo* enzyme design and enabled the creation of the first computationally designed biocatalysts for reactions such as ester hydrolysis (Bolon and Mayo 2001; Richter *et al.* 2012), Kemp elimination (Rothlisberger *et al.* 2008; Privett *et al.* 2012), retro-aldol condensation (Jiang *et al.* 2008; Althoff *et al.* 2012), Diels–Alder cycloaddition (Siegel *et al.* 2010), and Morita–Baylis–Hillman reaction (Bjelic *et al.* 2013). Although successful, these early enzymes exhibited modest activity, revealing limitations of the approach: imperfect conformational sampling, inaccurate scoring functions, and oversimplified mechanistic assumptions frequently produced misaligned active sites and weak transition-state stabilization.

**Figure 12:**
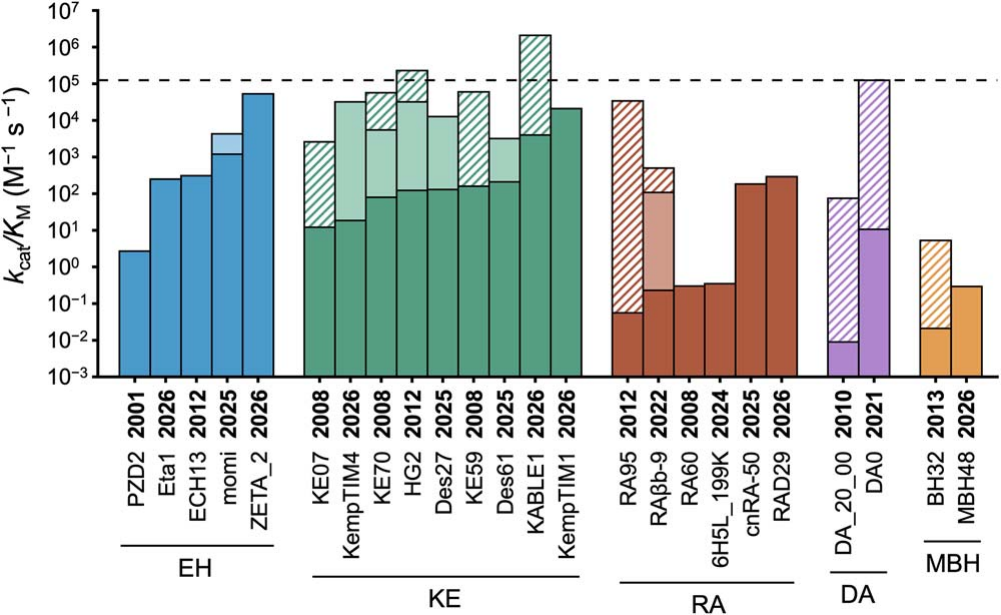
Catalytic efficiency of designed, optimized, and evolved *de novo* enzymes. Enzymes are grouped by reaction type and ordered by the catalytic efficiency of the first-round variant (dark bars), with its name and publication year listed below each column (Bolon and Mayo 2001; Jiang *et al.* 2008; Rothlisberger *et al.* 2008; Siegel *et al.* 2010; Althoff *et al.* 2012; Privett *et al.* 2012; Richter *et al.* 2012; Bjelic *et al.* 2013; Basler *et al.* 2021; Kipnis *et al.* 2022; Elaily *et al.* 2024; Anishchenko *et al.* 2025; Beck *et al.* 2026; Lauko *et al.* 2025; Listov *et al.* 2025; Ahern *et al.* 2026; Braun *et al.* 2026; Chen *et al.* 2026; Kim *et al.* 2026). Lighter bars represent variants optimized by computational design (Kipnis *et al.* 2022; Rakotoharisoa *et al.* 2024; Beck *et al.* 2026; Lauko *et al.* 2025; Listov *et al.* 2025), and hashed bars indicate variants optimized by directed evolution (Khersonsky *et al.* 2011, 2012; Blomberg *et al.* 2013; Preiswerk *et al.* 2014; Obexer *et al.* 2017; Basler *et al.* 2021; Crawshaw *et al.* 2022; Kipnis *et al.* 2022). The dashed line indicates the median *k*_cat_/*K*_M_ of natural enzymes (125 000 M^−1^ s^−1^) (Bar-Even *et al.* 2011). Reaction types EH, KE, RA, DA, and MBH, corresponding to ester hydrolysis, Kemp elimination, retro-aldol condensation, Diels–Alder cycloaddition, and the Morita–Baylis–Hillman reaction, are shown in blue, green, brown, purple, and orange, respectively. For enzyme reactions involving two substrates, *k*_cat_/*K*_M_ was calculated using the *K*_M_ for 4-carboxybenzyl *trans*-1,3-butadiene-1-carbamate, azachalcone, or 4-nitrobenzaldehyde for DA_20_00, DA0, or BH32 and MBH48, respectively. Catalytic efficiency for BH32 was taken from Crawshaw *et al.* (2022).

Over the past 5 years, the field has undergone a transformation driven by advances in AI, enhanced sampling, and *de novo* protein generation. Rather than adapting natural scaffolds, researchers can now design entirely new protein structures for more accurate theozyme incorporation. One strategy is to generate thousands of candidate backbone templates *in silico*, either through deep learning-based, family-wide hallucination of cavity-containing natural folds or combinatorial assembly of fragments from homologous TIM barrels. These backbones have supported designs of luciferases (Yeh *et al.* 2023), retro-aldolases (Anishchenko *et al.* 2025) and Kemp eliminases (Listov *et al.* 2025). In parallel, generative AI has enabled the construction of proteins around known catalytic arrangements derived either from theozyme models or from catalytic motifs with associated ligands extracted from the Protein Data Bank. These methods allow simultaneous design of backbones that precisely anchor catalytic residues and form substrate-specific active-site pockets, yielding enzymes for the retro-aldol condensation and Morita–Baylis–Hillman reaction (Braun *et al.* 2026), as well as for ester hydrolysis (Lauko *et al.* 2025; Ahern *et al.* 2026; Kim *et al.* 2026). More recently, generative AI has been used to customize minimalist *de novo* TIM barrels by adding structural lids that anchor catalytic residues and shape active-site pockets, enabling the design of Kemp eliminases (Beck *et al.* 2026). These methods produced more active initial designs than earlier approaches (Fig. 12), underscoring the advantages of building tailored structures rather than repurposing proteins that evolved for other functions. However, many of these advances primarily reflect improvements in sampling efficiency, throughput, and structural prediction accuracy rather than entirely new catalytic design principles.

There have also been numerous examples of *de novo* enzyme design focused on creating scaffolds that bind metal ions or organometallic cofactors mediating diverse bond-forming and bond-breaking reactions, where catalysis arises primarily from the cofactor’s intrinsic reactivity (Stenner *et al.* 2020; Mann *et al.* 2021; Berglund *et al.* 2024; Klein *et al.* 2024a; Zou *et al.* 2025a). Because these designs do not rely on theozymes, early scaffolds featured generic pockets not tailored to particular substrates. Recent work has refined these pockets using theozyme-based approaches to remodel active sites (Basler *et al.* 2021; Kalvet *et al.* 2023; Hou *et al.* 2025) or generative AI to construct them from scratch (Hoffnagle *et al.* 2025; Ahern *et al.* 2026; Kim *et al.* 2026), improving reactivity and selectivity. Beyond designing custom protein templates to anchor active sites, an orthogonal strategy exploits crystallographic fragment screening to reveal small-molecule binding sites on nascent scaffolds, guiding the placement of structurally related theozymes within these pockets (Chen *et al.* 2026). This workflow yielded one of the most active Kemp eliminases produced using a theozyme-based design approach.

A notable emerging trend is the advent of computational methods capable of boosting activities of initial designs by orders of magnitude without relying on directed evolution. Ensemble-based design employs conformational ensembles derived from X-ray crystallography to sample substates that more accurately represent the true transition-state ensemble, improving the accuracy of theozyme incorporation (Rakotoharisoa *et al.* 2024; Beck *et al.* 2026). An alternative approach, FuncLib, uses phylogenetic analysis and stability calculations to identify combinations of mutations at active-site positions that are both evolutionarily tolerated and energetically stable, thereby focusing the design space on variants compatible with the structural integrity of the scaffold (Risso *et al.* 2020; Listov *et al.* 2025). Both strategies have dramatically increased the catalytic efficiencies of Kemp eliminases by optimizing active-site geometry (Fig. 12), demonstrating that *in silico* optimization can rival early rounds of laboratory evolution without relying on high-throughput screening. Yet even these enhanced designs still fall short of natural enzymes in turnover rates and catalytic efficiencies, and generally exhibit lower activity than variants improved by extensive directed evolution. Closing this gap will likely require accounting for biological features still largely neglected in current design methods, such as conformational dynamics, long-range electrostatics, and allosteric interactions, as well as deeper mechanistic understanding of enzyme catalysis itself. Progress could also be accelerated by the generation of larger, more standardized datasets linking enzyme sequence, structure, and activity (Wang *et al.* 2018; Long *et al.* 2026; Yuan *et al.* 2026), analogous to the role the Protein Data Bank played in structural biology. With computational and generative AI approaches accelerating at unprecedented speed, *de novo* enzyme design is moving steadily toward the long-standing goal of producing useful biocatalysts on demand for real-world applications.

### Computational design of protein dynamics (Alfie-Louise R. Brownless and Shina Caroline Lynn Kamerlin^*^)

Proteins are dynamic entities that explore generally rugged conformational landscapes. As a result, multiple protein conformations are thermodynamically accessible, and the transitions among these metastable states are critical for mediating catalysis (Georgelin and Jackson 2025). Following the recognition of the importance of such dynamics for modulating protein evolvability (Tawfik’s “New View” of proteins (James and Tawfik 2003)), there has been increasing interest in also exploiting such dynamics for protein engineering purposes (Crean *et al*. 2020; Damry and Jackson 2021). However, altering protein function by manipulating conformational dynamics is challenging for two fundamental reasons: (1) protein dynamics are controlled by residues that span the full scaffold in non-obvious ways, and it is uncertain how modifications in one part of the scaffold will impact the functionality of a different region (even before taking into account epistatic effects (Campbell *et al.* 2018)) and (2) different strategies are needed to minimize unproductive conformations vs. enhance productive conformations, necessitating intimate knowledge of the conformational landscapes and residue communication networks within the particular protein target.

**Figure 13:**
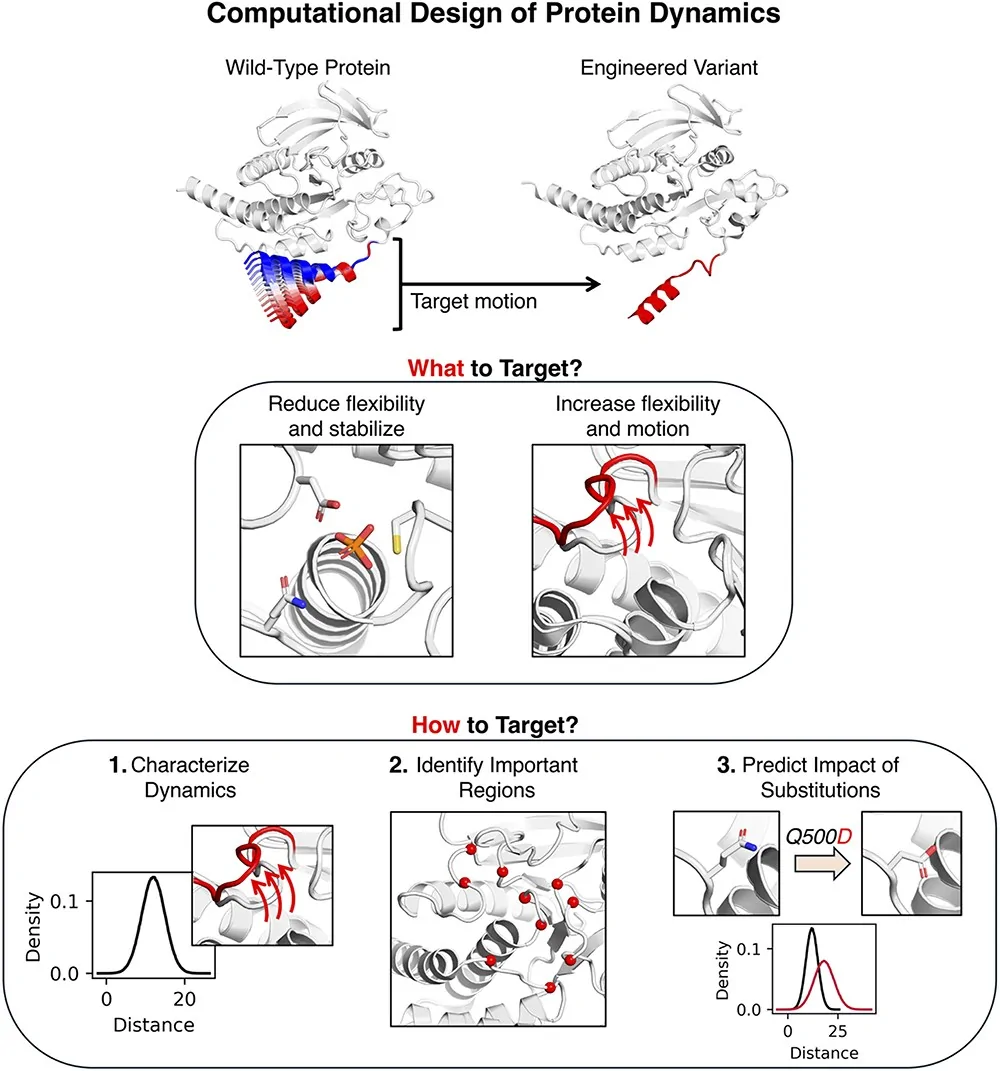
Fundamental concepts of computational design of protein dynamics. The choice of whether to aim to increase or reduce flexibility in a chosen region is often nontrivial and thus a major component of the design process. Computational methods allow for full characterization of dynamics, identification of regions which manage key dynamical motions, and accurate predictions of the effects of amino acid substitutions at these positions. Continuous advances in implementation of computational methodologies will allow for more efficient and successful engineering prospects *via* strategic tailoring of dynamical properties.

Despite these challenges, there has been significant progress in using both experimental and computational methods to characterize functionally important protein dynamics, identify regions linked with those dynamics, and predict the impact of amino acid substitutions at those positions. We will focus here on computational advances (Fig. 13), and for reviews of experimental advances, we refer readers to previously published works (Campbell *et al.* 2018; Damry and Jackson 2021). Importantly, the early-stage developments of highly accurate forcefields, hybrid QM/MM methods, and enhanced sampling approaches cannot be overstated in their impact toward modern computational methods (Jorgensen 2013). In particular, early forcefield developments by Jorgenson, Karplus, Case, Kollman, Gunsteren, and others (Kaminski *et al.* 2001; Wang *et al.* 2004; Brooks *et al.* 2009; Schmid *et al.* 2011; Hwang *et al.* 2024), and the emergence of QM/MM hybrid methods pioneered by Warshel and Levitt, and others (Levitt and Warshel 1975; Warshel and Levitt 1976), provided the foundations upon which modern simulation protocols continue to rely and iterate upon. With more modern infrastructure available, the scale of computational accessibility has increased dramatically. As a non-exhaustive overview of earlier computation-driven innovation of protein design, computational approaches have succeeded in designing peptides which can adopt both a zinc finger fold and a trimeric coiled coil fold (Ambroggio and Kuhlman 2006) as well as proteins which exhibit desired conformational shifts among targeted states by optimizing for energetically accessible states using meta-multistate design (Davey *et al.* 2017). Specifically, meta-multistate design was used to select sequences derived from the Streptococcal protein G domain β1 fold which exhibit notable exchange between two targeted conformational states, a trait which, remarkably, is not seen in the wild-type sequences (Davey *et al.* 2017). More recent advances include Conformational Biasing (CB), which has allowed for mutations which strategically alter protein dynamics to be rapidly and accurately predicted (Cavanagh *et al.* 2026), as well as new approaches for designing protein systems with tailored allosteric properties (Pillai *et al.* 2024). Finally, Kemp eliminases have provided an excellent model system for testing conformational design strategies, including the use of structural ensembles to generate variant sequences with catalytic efficiencies increased by 100–250-fold compared to wild-type (Rakotoharisoa *et al.* 2024), as well as the combination of NMR-based hotspot selection and ML (Bhattacharya *et al.* 2022) or FuncLib (Khersonsky *et al.* 2018; Gutierrez-Rus *et al.* 2025) to obtain Kemp eliminases with catalytic proficiencies comparable to natural enzymes.

Looking forward, although accurately capturing protein dynamics remains challenging, and limited experimental data sets and training set biases complicate reliable predictions, the rapid evolution of advanced deep learning and generative AI approaches for modeling protein conformational ensembles will significantly push forward the boundaries of this field (Brownless *et al.* 2025). Although such models typically ignore conformational dynamics, advances in this space such as BioEmu (Lewis *et al.* 2025) and ESMDynamic (Kleiman *et al.* 2025) among others (Brownless *et al.* 2025) could be used in tandem with supplemental analysis tools used to identify residues which regulate conformational changes and those involved in allosteric pathways (Romero-Rivera *et al*. 2017; Crean *et al.* 2023; Raju *et al.* 2025). Further, such approaches would substantively reduce computational cost associated with the need otherwise for exhaustive dynamical simulations. Strategic combining of these methods will allow for more accurate and efficient procedures for incorporating consideration of conformational ensembles into computational design workflows, making it eventually possible to tune conformational dynamics to facilitate specific function(s).

### Computational design of membrane proteins (Jingyi Zhu and Peilong Lu^*^)

Transmembrane proteins carry out many important biological functions. However, their *de novo* design is more challenging than that of soluble proteins due to the complex membrane environment and the difficulties in experimental characterizations. While the general design principle for transmembrane proteins is consistent with that of soluble proteins, key differences exist: (1) transmembrane proteins typically adopt an all-α or all-β topology in order to traverse the membrane, and (2) Their surface features a highly hydrophobic transmembrane region that interacts with the lipid bilayer, and hydrophilic extramembrane regions that interact the aqueous environment. Early efforts included designing transmembrane oligomers (Mravic *et al.* 2019), ion-conducting (Lear *et al*. 1988; Scott *et al.* 2021) or transporting proteins (Joh *et al.* 2014), and interactions with porphyrin molecules (Korendovych *et al.* 2010). However, these attempts were limited to the assembly of chemically synthesized single-span peptides, and the compatibility of these designs within living organisms remains to be explored.

**Figure 14:**
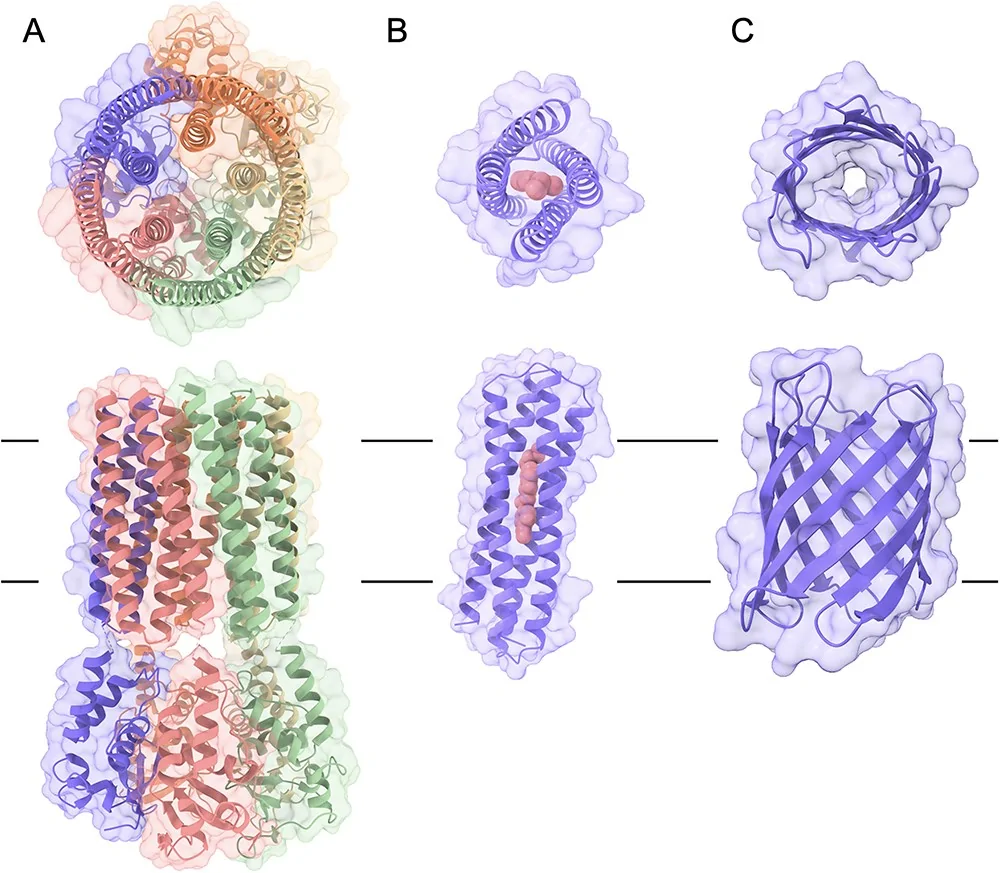
Representative functional *de novo* transmembrane proteins. (A) *De novo* designed helical ion channel (Zhou *et al.* 2025). (B) *De novo* designed fluorophore-binding transmembrane protein (Zhu *et al.* 2025). (C) *De novo* designed β-sheet-based nanopore (Berhanu *et al.* 2024). The top row shows the top view, the bottom row shows the side view. The black lines indicate the approximate position of the membranes.

The advance of computational protein design methods has enabled the design of multi-pass transmembrane proteins (Lu *et al.* 2018), allowing *de novo* design of more complicated structures and functions in membranes. Parametric generation of helical bundles allowed the construction of transmembrane pores with specific ion conductance (Xu *et al.* 2020). Voltage-gating was achieved by strategic placement of charged residues in helical channels (Zhou *et al.* 2025) (Fig. 14). Diffusion-based scaffold generation methods enabled bottom-up design of calcium channels based on its selectivity filter (Liu *et al.* 2025c). Iterative sequence optimization using structure prediction networks has allowed the accurate design of noncovalent small molecule binding and bright fluorescence activation in membranes (Zhu *et al.* 2025). Progress has also been made in designing transmembrane β-barrels, using fragment assembly methods with optimization of the loop sequences (Vorobieva *et al.* 2021). Recently, the use of deep learning-based scaffold generation methods has enabled parametric design of β-barrels as well (Kim *et al.* 2025a). A series of transmembrane β-barrels with various pore sizes were designed, showing different ion conductance profiles in lipid bilayers (Berhanu *et al.* 2024). The designed nanopore was further engineered to become ligand-gated through fusion of a soluble chemical-induced dimerization domain (An *et al.* 2024b).

The rapid advancement of deep learning-based protein design and structure prediction methods (Jumper *et al.* 2021; Dauparas *et al.* 2022; Watson *et al.* 2023; Abramson *et al.* 2024), although developed for protein design in general, has greatly facilitated transmembrane protein design, especially in improving the accuracy and efficiency of generating scaffolds and sequences with desired properties. Nevertheless, challenges remain. Membrane proteins reside in a highly dynamic and complicated environment, making them intrinsically hard to study: the cell membrane contains a high concentration of lipids and a strong membrane potential across it, and the exact folding and biogenesis mechanism of membrane proteins remains elusive. The training of deep learning-based methods specific for membrane protein sequence design has therefore been difficult due to the scarcity of appropriate data sets. Furthermore, structural characterization of these proteins continues to pose significant challenges. Fortunately, advances in cryo-electron microscopy (cryo-EM) over the past decade have facilitated structural determination. By designing fusion domains, cryo-EM structure determination of small membrane proteins is now becoming feasible (Liu *et al.* 2025c; Zhou *et al.* 2025; Zhu *et al.* 2025). Taken together, the field is progressing toward the design of *de novo* transmembrane proteins with increasingly complicated functions and dynamics, such as gating of the transmembrane pores, biosensing, catalysis, and ligand transport.

## Enzymes

### Engineering cytochrome P450 enzymes (Douglas J. Fansher and Joelle N. Pelletier^*^)

The heme-containing cytochrome P450 monooxygenases (P450) natively catalyze a wide range of reductive oxidation reactions. They provide a valuable approach to late-stage modification of small molecules without necessitating protection steps (Fansher *et al.* 2024). Their particular interest stems from reactivity on non-activated C-H bonds, giving selective access to chemically unattainable products. The electron source, NADPH, transfers two electrons to FAD, and further to the heme-bound oxygen *via* three domains which either belong to separate polypeptides or to a single protein. P450s are mainly applied to synthesis of value-added chemicals or to produce drug metabolites. For instance, the engineered CYP105AS1 performs a key hydroxylation for industrial production of the cholesterol-lowering drug pravastatin (McLean *et al.* 2015; Ashworth *et al.* 2022). The potential of P450s for bioremediation of persistent pollutants such as polyaromatic hydrocarbons has also been explored.

**Figure 15:**
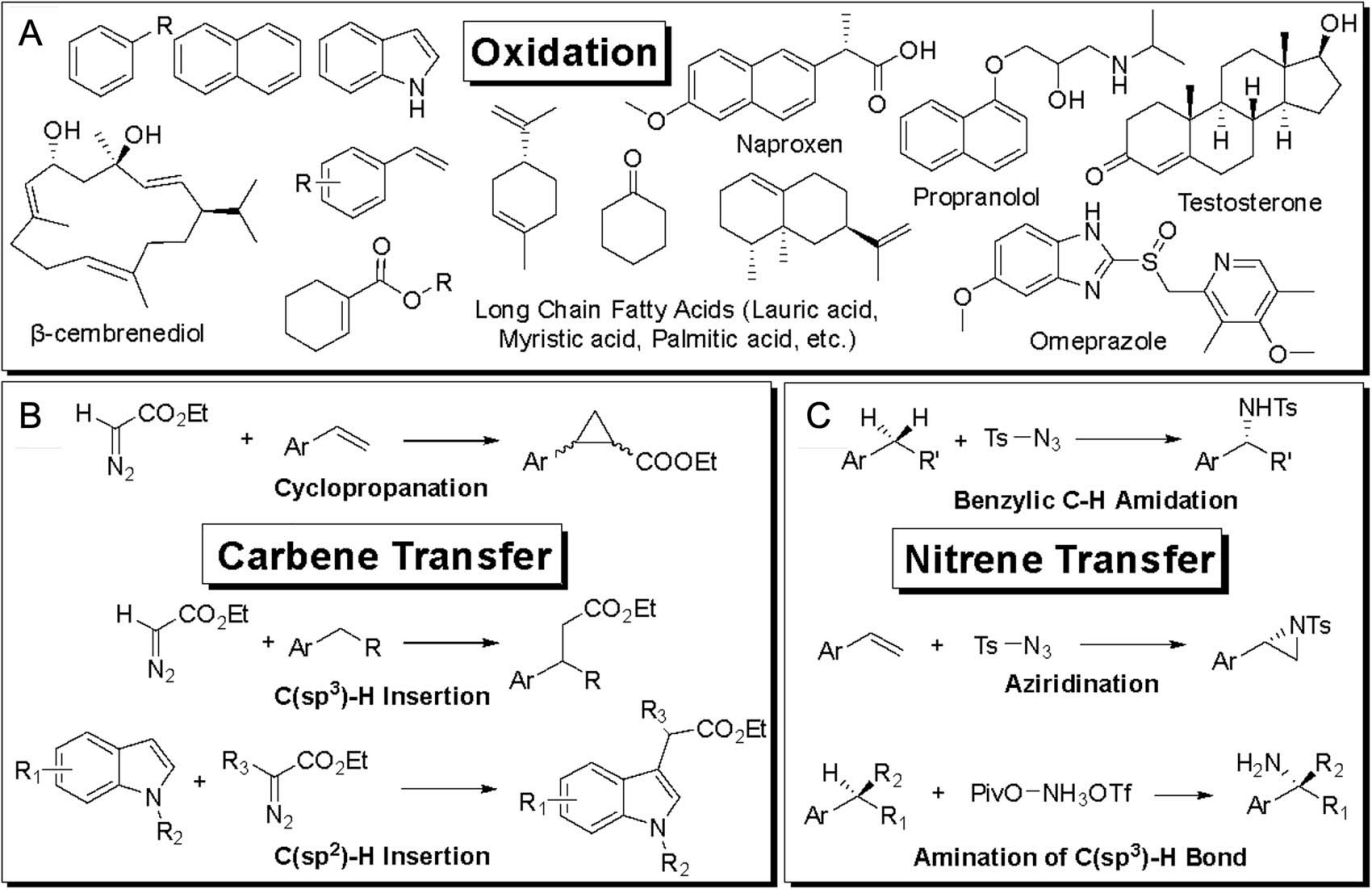
Overview of reactions catalyzed by native and engineered P450 BM3. (A) Representative substrates involved in oxidation reactions. (B) Representative carbene-transfer reactions and (C) nitrene-transfer reactions.

Members of the P450 superfamily, both native and engineered, from animals, plants or microbes, have been applied to synthetic reactions including regioselective fatty acid oxidation, regio- and stereoselective steroid oxidation, drug metabolism, and epoxidation of olefins. For instance, P450 enzymes from fungus, toad, plants, and microbes were recently reported in diversification of steroids, procuring hydroxylation at a variety of sites (Chen *et al.* 2020; Peng *et al.* 2022; Zhao *et al.* 2022). The P450 most frequently engineered is P450_BM3_ (CYP102A1), from *Bacillus megaterium* (reclassified as *Priestia megaterium* in 2020 (Gupta *et al.* 2020)). Contrary to membrane-bound P450s, which are more common in eukaryotes, this soluble self-sufficient enzyme functions as a single polypeptide, contributing to its fast electron transfer and high catalytic efficiency. Recent successes, summarized in several reviews (Ren and Fasan 2021; Yang and Arnold 2021; Fansher *et al.* 2024), include altering regio- and enantioselectivity in oxidation of fatty acids and steroids, oxidation of small alkanes as biofuels, aromatic, and olefin oxidation to generate phenols and epoxides, oxidation of drugs and bioactive natural products (Fig. 15A). Other examples of new-to-nature reactions include carbene (Fig. 15B) (Coelho *et al.* 2013) and nitrene transfer reactions (Fig. 15C), which will be further discussed in Section Engineered metalloenzymes for new-to-nature reactions (Rudi Fasan^*^).

Discovery of further P450s (the “P450ome”) (Wang *et al.* 2024b) by genomic and metagenomic approaches, or using ASR (see Section Ancestral sequence reconstruction (Gabriel Foley and Elizabeth M. J. Gillam^*^)) (Harris *et al.* 2022), continues to expand their scope of applications. Whereas engineering—in particular of P450_BM3_—was earlier investigated as an alternative to hepatic P450s to evaluate drug metabolites during drug development (Thistlethwaite *et al.* 2021), discovery of diverse P450s is now favored. For engineering, soluble P450s are favored over membrane-bound variants; self-sufficient P450s are preferred for ease of expression and better electron transfer rates, boosting efficiency. Remaining protein engineering challenges concerning biocatalysis include improving substrate specificity and activity, stereo- and regioselectivity, enzyme stability, cost of an electron source—even when using enzyme-coupled recycling systems, coupling efficiency with non-native substrates, and solvent tolerance. Methods to improve screening throughput, including computational approaches, will be beneficial. Using H_2_O_2_ as a co-substrate is an opportunity to replace NADPH yet requires engineering to mitigate loss of stability; recent progress is promising (Podgorski *et al.* 2024). Thus, scalability must be addressed to move the field beyond fine chemicals and pharmaceuticals.

In the future, unspecific peroxygenases (UPO) may become an attractive alternative to P450s. These heme-dependent oxygenases are readily obtained at a large scale and use exogenous H_2_O_2_, reducing application cost. Their substrate promiscuity suggests that engineering broader reactivity is feasible, as demonstrated for targets in drug metabolism (Gomez de Santos *et al.* 2018), and biocatalysis (Aukema *et al.* 2022; Münch *et al.* 2023). Their high activity and stability lend themselves to reaction scale-up, demonstrated up to half a kilogram scale (Hilberath *et al.* 2023). Alpha-ketoglutarate-dependent oxygenases, which do not require NADPH, are also attractive alternatives to P450 biocatalysts and are seeing considerable adoption (He *et al.* 2024).

### Flavin-dependent enzymes (Kridsadakorn Prakinee and Pimchai Chaiyen^*^)

The majority of flavin-dependent enzymes (flavoenzymes) mediate redox processes such as monooxygenation, oxidation, dehydrogenation, or electron transfer of organic substrates. Research groups worldwide have elucidated reaction mechanisms of these enzymes using diverse kinetic and mechanistic approaches, providing in-depth insights into how enzymes such as monooxygenases (Paul *et al.* 2021; Phintha and Chaiyen 2023), oxidases (Trisrivirat *et al.* 2022), dehydrogenases (Tan *et al.* 2015), reductases (Pimviriyakul *et al.* 2025), electron-transferring flavoproteins (Buckel and Thauer 2018), and non-redox flavin-dependent enzymes catalyze their reactions. These fundamental studies are key to supporting successful engineering of flavin-dependent enzymes. Over recent decades, we have seen growing applications of engineered flavoenzymes in biocatalysis, as highlighted below.

A combination of rational design and protein dynamics-guided approaches has been applied to engineer old yellow enzymes or ene-reductases, to obtain variants for efficient asymmetric synthesis (Yin *et al.* 2025a). These flavoenzymes have significantly advanced asymmetric biocatalysis, achieving transformations that are challenging through traditional synthetic routes. Significant ground-breaking research on ene-reductases has also demonstrated their potential, particularly in combination with photoactivation processes, enabling non-natural photoenzymatic reactions such as enantioselective decarboxylative alkylation (Sun *et al.* 2024), stereo-controlled radical hydroarylation (Zhao *et al.* 2023), and asymmetric synthesis of fluorinated compounds (Li *et al.* 2024). The formation of oxidative radicals is key to these versatile biotransformations, which exhibit remarkable chemo- and enantio-selectivity. These intriguing, broad-scope reactions will undoubtedly continue to expand in the future.

**Figure 16:**
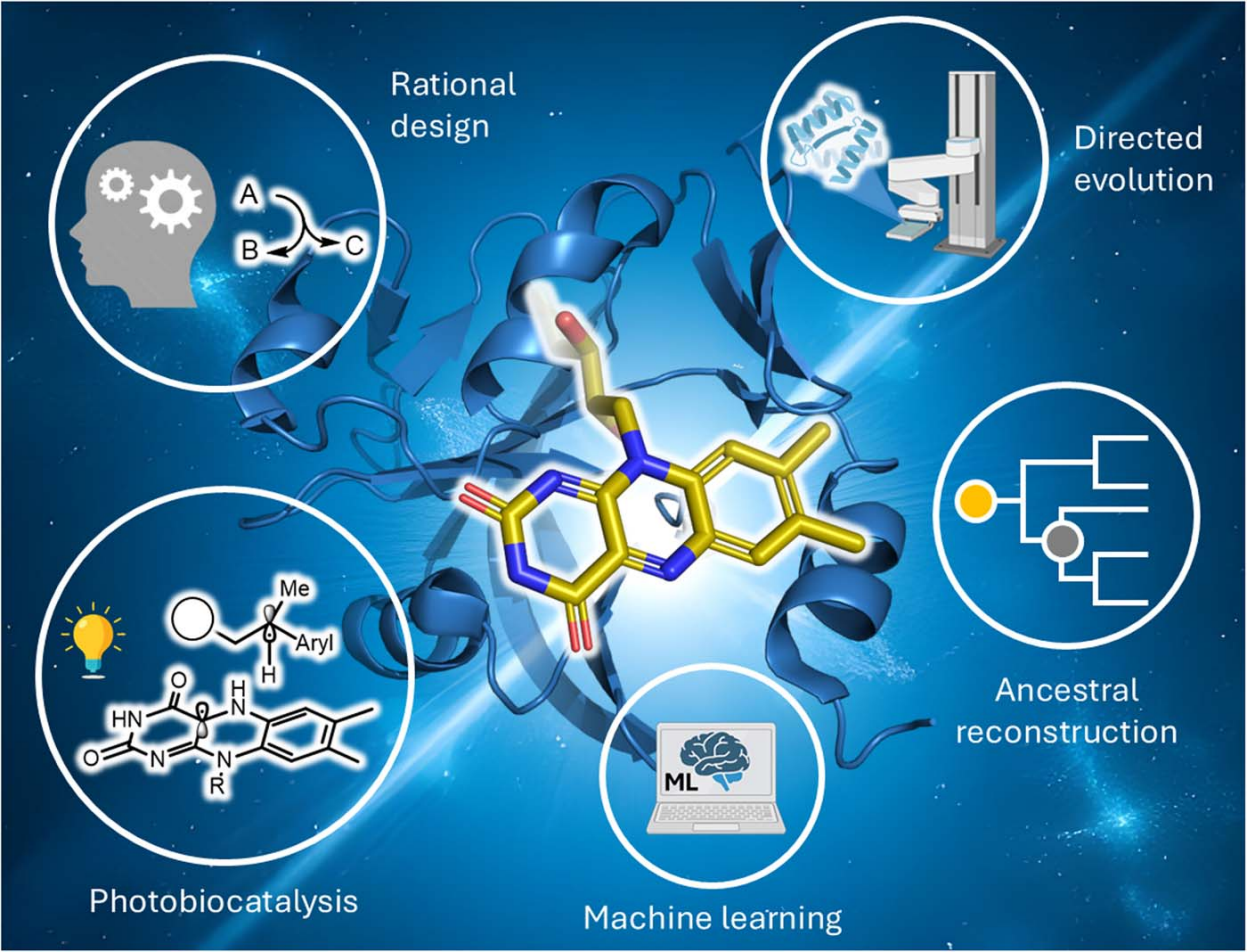
Overview of approaches used to engineer flavin-dependent enzymes.

Enzyme engineering based on rational design and directed evolution has proven highly effective in unlocking potentials of flavoenzymes beyond their native functions (Fig. 16). For instance, understanding how different oxidation mechanisms are governed by various flavoenzymes can be exploited for reprogramming their reactivity (Guerriere *et al.* 2025; Kumaran *et al.* 2025). A recent discovery has shown that monooxygenases and oxidases can be repurposed as non-native halogenase scaffolds (Phintha *et al.* 2021; Prakinee *et al.* 2024). Identification of bottlenecks in flavin-dependent halogenases has led to the engineering of substrate tunnels to transport reactive intermediates, thereby increasing halogenation efficiency (Prakinee *et al.* 2022). A semi-rational approach has also been applied to improve the thermostability of a flavin-dependent dehalogenase, yielding a variant later used for biodetoxification and *in situ* detection of pesticides (Pongpamorn *et al.* 2019). Similar approaches have successfully engineered an enzyme which can convert tryptophan to a plant hormone precursor (Kongjaroon *et al.* 2024). Likewise, Baeyer–Villiger and styrene monooxygenases—promising biocatalysts for the synthesis of esters and epoxides, respectively—have been engineered using structural and mechanistic insights (Li *et al.* 2018, 2025a). These developments highlight a powerful and unified framework: leveraging mechanistic insights to guide enzyme engineering and obtain desirable flavin biocatalysts.

Research on flavoenzyme engineering has also entered the digital era. Evolutionary-based ASR (see Section Ancestral sequence reconstruction (Gabriel Foley and Elizabeth M. J. Gillam^*^)), in combination with computational and mutagenesis studies, has provided valuable insights into the structure and functions of flavoenzymes, revealing how specific structural features control stereoselectivity and chemoselectivity in monooxygenases (Bailleul *et al.* 2023; Chiang *et al.* 2023), and enabling the creation of highly thermostable enzymes for D-amino acid synthesis (Kawamura *et al.* 2023). Moreover, ML-guided protein engineering has recently been applied to improve the hydroxylation activity of a phenolic hydroxylase through rational-guided library design (Matsushita *et al.* 2024). These recent works demonstrated that the traditional evolutionary and rational methods are still important as guiding foundations for the computational design. Although this data-driven approach to engineering flavoenzymes is still in its early stages, it is expected to mature and expand over time. With the growing depth of structural and functional understanding of flavoenzymes, this field is poised to grow and even more remarkable discoveries are yet to be seen.

### Engineered metalloenzymes for new-to-nature reactions (Rudi Fasan^*^)

**Figure 17:**
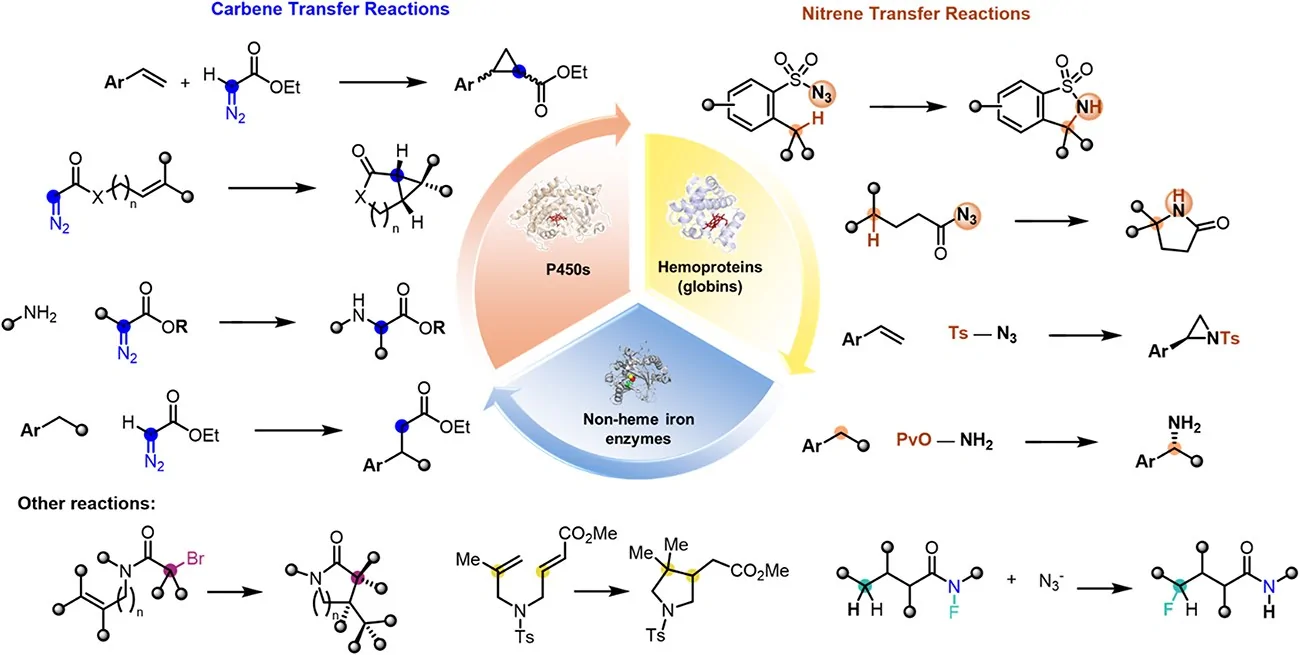
New-to-nature reactions catalyzed by engineered metalloenzymes.

A breakthrough in the field of enzyme engineering has involved the “repurposing” of metalloenzymes as biocatalysts for “new-to-nature” chemistries, that is chemical transformations not found in nature (Fig. 17). This field was initiated by the extension of the reaction scope of engineered heme-dependent enzymes and hemoproteins to abiological carbene and nitrene transfer reactions (Yang and Arnold 2021; Couture *et al*. 2024). Traditionally carried out using transition metal catalysts, these new enzymatic transformations enable the synthesis of medicinally important compounds such as cyclopropanes, amines, and thioethers, among others.

Seminal contributions in this area have demonstrated the ability of engineered cytochrome P450 enzymes (i.e. P450_BM3_) as well as hemoproteins (i.e. myoglobin) to catalyze the cyclopropanation of olefins with high degrees of catalytic efficiency and stereoselectivity (Coelho *et al.* 2013; Bordeaux *et al*. 2015). These advances have later extended to the development of efficient biocatalysts, based on engineered variants of different hemoproteins (e.g. cytochrome P450s, (myo)globins, cytochrome c, dehaloperoxidase, *etc.*) for the stereoselective synthesis of enantioenriched thioethers, amines, silanes, and organoboron compounds *via* carbene insertion reactions into Y-H bonds (Y = S, N, Si, B) (Tyagi *et al*. 2015; Kan *et al.* 2017; Steck *et al.* 2020a). Initially utilizing diazoesters as carbene donor reagents, the reaction scope of engineered “carbene transferases” have been later extended to a variety of other carbene precursors, including diazofluoroalkanes, diazoketones, diazirines, and pyridotriazoles, greatly expanding the spectrum of chiral products and synthetically valuable building blocks accessible through biocatalysis (Tinoco *et al.* 2017; Porter *et al.* 2022; Roy *et al.* 2024b). In addition, hemoprotein-catalyzed carbene transfer reactions have been developed for the functionalization of C(sp^3^)-H bonds (Zhang *et al.* 2019; Ren *et al.* 2023), a reaction previously accessible only using synthetic catalysts or artificial enzymes containing rare transition metals (e.g. Rh, Ir) (Dydio *et al.* 2016).

Concurrent with their exploration as “carbene transferases,” heme-dependent enzymes and proteins were shown to constitute promising biocatalysts for promoting abiological nitrene transfer reactions, which are synthetically important C-N bond forming reactions (Yang and Arnold 2021; Couture *et al*. 2024). Progress in this area led to the development of efficient and stereoselective biocatalysts for the synthesis of chiral sultams (Singh *et al*. 2014), aziridines (Farwell *et al.* 2015), carbamates, sulfamates (Yang *et al.* 2019), lactams (Roy *et al.* 2024a), and amines (Prier *et al.* 2017), *via* intramolecular and intermolecular C-H amination reactions. Beyond carbene and nitrene transfer reactions, engineered heme enzymes, and proteins were recently shown to catalyze other interesting abiological transformations such as radical-mediated cyclizations *via* atom transfer (Zhou *et al.* 2021; Fu *et al.* 2024) and metal-hydride hydrogen atom transfer mechanisms (Zhang *et al.* 2025b).

Along with hemoproteins, other classes of metalloenzymes such as non-heme iron dependent enzymes have been shown to hold great promise for the engineering of biocatalysts for new-to-nature chemistries. In nature, these enzymes utilize reactive iron-oxo intermediates to catalyze a variety of oxidation reactions, including hydroxylation, halogenation, epoxidation, and rearrangement reactions (Neidig and Solomon 2005). Over the past few years, these enzymes were shown to be versatile and evolvable scaffolds for catalyzing a number of non-native, abiological reactions, such as transfer-mediated C-H amination and aziridination reactions as well as C(sp^3^)-H azidations and alkyne difunctionalization *via* oxidative coupling reactions involving photochemically generated radical intermediates (Rui *et al.* 2022, 2024; Zhang *et al.* 2024b).

In addition to directed evolution, computational design, structure- and mechanism-guided rational design, and ML-guided protein engineering have represented valuable approaches for tuning or enhancing the catalytic properties of engineered metalloenzymes for abiological chemistries (Wu *et al.* 2019; Steck *et al.* 2020b; Ding *et al.* 2024; Vargas *et al.* 2024). Various studies have begun to showcase key advantages of these systems for synthetic applications. For example, enzyme-catalyzed new-to-nature reactions have been shown to enable more efficient and stereoselective chemoenzymatic routes for the synthesis of drug molecules (Bajaj *et al.* 2016; Roy *et al.* 2024a) or even enable transformations not previously accessible *via* chemical catalysis. Other studies have demonstrated the possibility to integrate abiological carbene transfer reactions with reconstituted biosynthetic pathways for producing new chemicals using engineered organisms (Huang *et al.* 2023). Overall, the protein engineering of metalloenzymes and other cofactor-dependent enzymes for new-to-nature chemistries is a rapidly expanding field that it is expected to continue to grow and evolve in the coming several years.

### Chemoenzymatic synthesis (Henrik Terholsen and Sandy Schmidt^*^)

Emulating nature’s capacity to generate high-value-added organic molecules has spurred the integration of enzymatic transformations in synthetic methodologies, particularly for the production of chiral active pharmaceutical ingredients (APIs). The field originated in the mid-20th century with early biocatalytic processes (Bornscheuer and Buchholz 2005), and the discovery of stable, selective enzymes such as lipases later drove their industrial adoption (Buller *et al.* 2023; Bayer *et al.* 2025). In the pharmaceutical industry, the synthesis of chiral intermediates is of particular interest due to the high stereoselectivity, regioselectivity, and substrate specificity of enzymes. However, bulky substrates and process parameters such as temperature, pH value, substrate loading, and cosolvent concentration are often incompatible with wild-type enzymes. In these cases, enzyme engineering, including random mutagenesis and (semi)rational design is frequently used to develop chemoenzymatic routes for synthesizing pharmaceuticals with the desired properties. Furthermore, incorporating (engineered) enzymes into chemical technologies can dramatically shorten synthetic pathways, resulting in reduced waste generation and improved cost efficiency.

**Figure 18:**
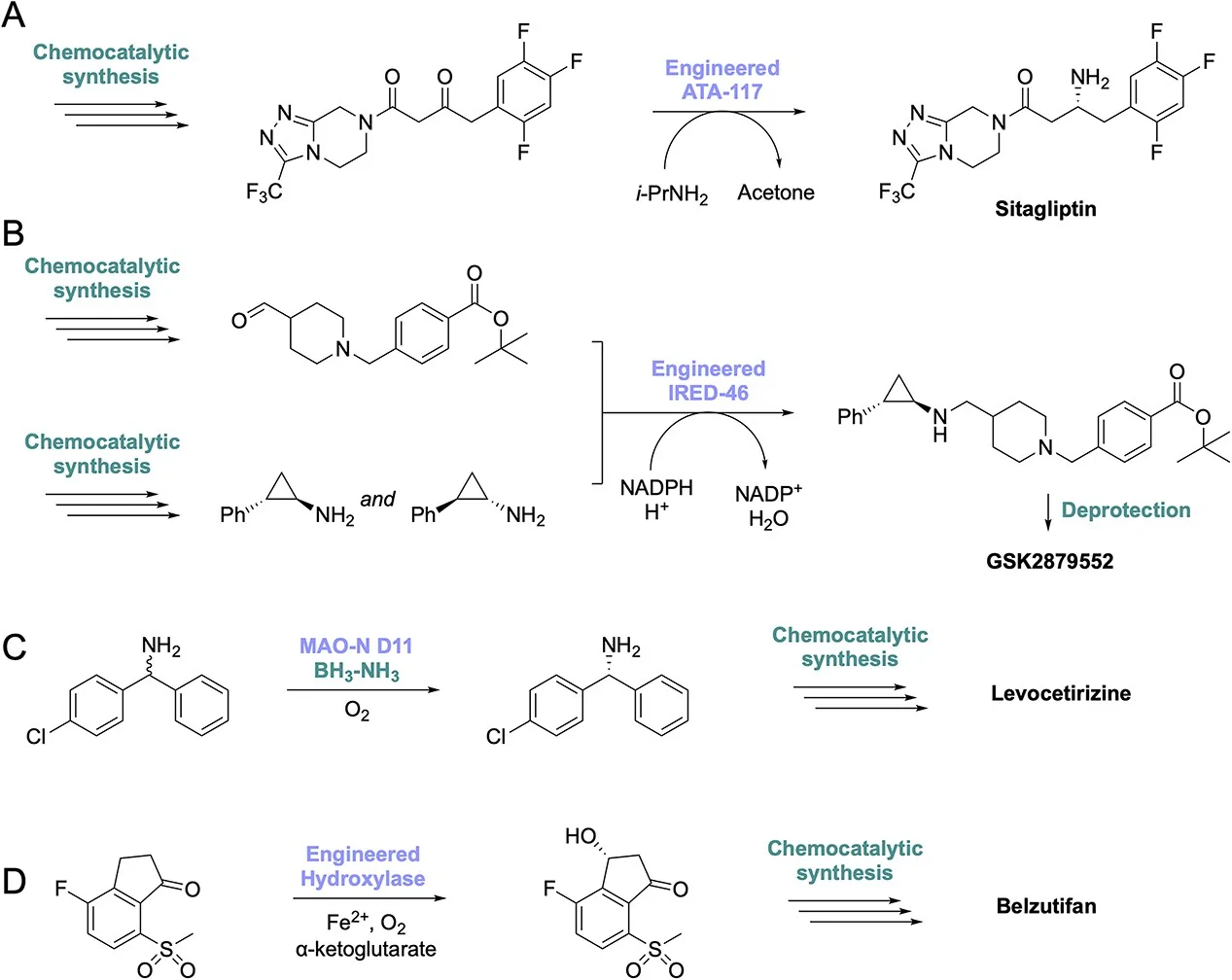
Engineered enzymes for the chemoenzymatic synthesis of APIs. (A) Enantioselective transamination in the synthesis of sitagliptin; (B) racemic resolution of the key intermediate of GSK2879552 using an engineered IRED; (C) deracemization of levocetirizine precursors; and (D) selective hydroxylation in the synthesis of belzutifan.

As early as 2007, Codexis combined multivariate optimization theories, genetic recombination, and laboratory evolution methodologies to improve the biocatalytic function of a halohydrin dehalogenase (HHDH). The conversion efficiency of (*S*)-4-chloro-3-hydroxybutyrate to ethyl (*R*)-4-cyano-3-hydroxybutyrate in the presence of cyanide was increased 4000-fold and has been used in the production of the cholesterol-lowering drug atorvastatin (Lipitor) (Fox *et al.* 2007). Another important example of an engineered enzyme incorporated into a synthetic route is the use of a transaminase in the amination reaction of prositagliptin ketone during the final step of sitagliptin synthesis (Savile *et al.* 2010) (Fig. 18A). The (*R*)-selective transaminase ATA-117 was engineered through 11 rounds of library screening that combined rational and random design strategies. This yielded a highly active variant capable of using high substrate loads (200 g/l) in the presence of 50% DMSO. Another industrial example is the evolution of an enantioselective imine reductase (IRED) in the core step of a chemoenzymatic synthetic route, which resulted in a commercially relevant manufacturing process to lysine-specific demethylase-1 inhibitor GSK2879552 (Schober *et al.* 2019) (Fig. 18B). The evolution of the IRED resulted in an enzyme variant that outperformed the wild type by over 38 000-fold and enabled the optimization of the process to a level that is economically viable at the kilogram scale. In addition to transaminases and IREDs, monoamine oxidase from *Aspergillus niger* (MAO-N) has been engineered for the production of enantiomerically pure chiral amines (Fig. 18C). Using a combination of random mutagenesis and rational design increased the substrate scope to accommodate amine substrates with bulky aryl substituents (Ghislieri *et al.* 2013). These variants were then used to deracemize several pharmaceutical building blocks in combination with a regenerating chemical reductant (Dunsmore *et al.* 2006; Rowles *et al.* 2012). Another example of an engineered oxidizing enzyme used in chemoenzymatic synthesis is the non-heme iron/α-ketoglutarate-dependent dioxygenase FoPip4H (Fig. 18D). This enzyme enables the direct, stereoselective, and regioselective hydroxylation of a fluoroindanone intermediate, replacing five steps in the Belzutifan synthesis process (Cheung-Lee *et al.* 2024).

These examples showcase how combining multiple catalytic steps involving (engineered) enzymes can achieve highly precise chemical transformations, enhancing the efficiency, sustainability, and applicability of biocatalysis in organic synthesis for producing chiral APIs. Furthermore, they demonstrate that target process parameters must be identified and applied in the initial screening rounds of evolution campaigns. This allows for the development of a tailor-made enzyme for the process, in line with the general principle of directed evolution (Schmidt-Dannert and Arnold 1999): “You only get what you screen for.” In the near future, biocatalysis will address increasingly complex reactions through the development of new biocatalysts and chemoenzymatic strategies. Integrating multiple enzymes with complementary methodologies derived from photo- and/or electrochemistry into chemoenzymatic processes promises the efficient synthesis of complex molecules. Additionally, the emergence of ``unnatural biocatalysis'' will facilitate efficient and stereoselective C–C and C–heteroatom bond formations *via* directed enzyme evolution. This emerging wave of biocatalysis is expected to create novel opportunities for advancing cutting-edge technologies and enabling the cost-effective production of chiral APIs. Ultimately, fine-tuning computationally designed enzymes for specific reactions and integrating AI and ML into enzyme engineering will be essential to unlocking new catalytic functions and promoting greener industrial processes. This will be especially true for applications in non-aqueous solutions, facilitating the use of enzymes in chemoenzymatic processes.

### Enzymatic degradation of synthetic polymers (Andreas Gagsteiger, Onur Turak, and Birte Höcker^*^)

Naturally occurring and engineered enzymes have demonstrated the ability to degrade resilient biogenic polymers such as cutin, cellulose, lignin, and chitin. These enzymes have also been investigated for their potential to break down chemically related synthetic polymers including highly robust plastics. In the 1990s microbial cultures were commonly used to assess polymer degradation. One of the earliest isolated enzymes shown to degrade polyethylene terephthalate (PET) was *Tf*H (Müller *et al.* 2005). Later, a great source of enzymes turned out to be metagenomes from sites where natural polymers accumulate e.g. compost searches led to identification of the leaf-branch compost LC-cutinase (Sulaiman *et al.* 2012). Many other hydrolases were identified, of which most depolymerize PET (PETases) but also other polymers such as polyurethane (see plastic-active enzyme database, PAZy (Buchholz *et al.* 2022)). The growing interest in engineering enzymes for synthetic polymer degradation correlates strongly with public concern over plastic waste mismanagement. A major milestone was the discovery of a multi-enzyme PET degradation pathway in *Ideonella sakaiensis* in 2016 (Yoshida *et al.* 2016) which highlighted the potential for biological recycling and inspired efforts to identify and optimize more efficient enzymes. Key strategies included metagenome mining for novel enzyme candidates and engineering of these enzymes to enhance PET degradation performance.

**Figure 19:**
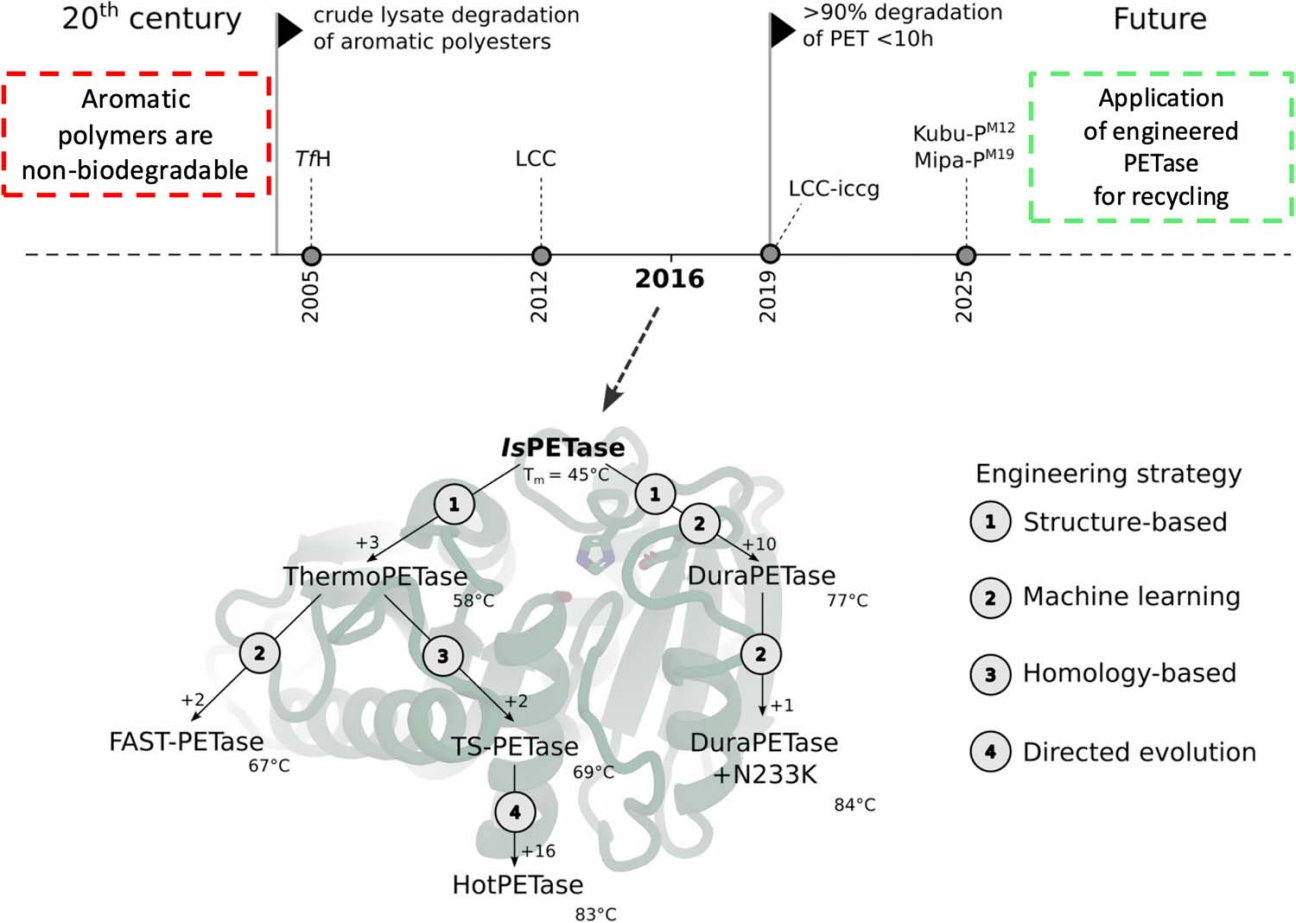
Progress towards enzymatic recycling using engineered PETases. Before the first isolated PETase from *Thermobifida fusca*, *Tf*H, in 2005, it was assumed that aromatic polyesters such as PET are non-biodegradable. Over time, PET hydrolysis rates were improved mostly by increasing the melting temperatures (*T*_m_) of PETases to operate closer to the glass-transition temperature of PET and increase their thermostability. With the discovery of *Ideonella sakaiensis* PETase (*Is*PETase) in 2016, extensive engineering campaigns were started to increase its *T*_m_. Several methodologies were applied (strategies 1 to 4) which led to variants such as FAST-PETase (*T*_m_ = 67°C), HotPETase (*T*_m_ = 83°C) or DuraPETase (*T*_m_ = 77°C). The number of mutations added to each variant is indicated by “+*N*” at the arrow heads.

Numerous studies focused on increasing enzyme thermostability to enable activity near the glass-transition temperature of PET. These engineered variants exhibit significantly improved degradation efficiency due to accelerated reaction kinetics and increased substrate accessibility enabled by enhanced polymer chain mobility at elevated temperatures. Several potent enzymes were engineered based on *Is*PETase, such as DuraPETase, FAST-PETase, or HotPETase (Wei *et al.* 2022). These variants were generated using diverse protein engineering strategies such as directed evolution, computational design, and AI-based predictions (see Fig. 19), but other enzymes like PHL7 (Sonnendecker *et al.* 2022) and LC-cutinase (Sulaiman *et al.* 2012) have also been successfully optimized for use in industrial recycling processes. This includes robustness against changes in reaction conditions, such as fluctuations in temperature, pH, ionic strength, and resistance to product inhibition. The most efficient enzymes can depolymerize preprocessed post-consumer PET almost completely within 10 hours. However, while these enzymes perform well on amorphous PET, the degradation of highly crystalline regions remains a major challenge. One of the difficulties in engineering and design of these enzymes remains the limited understanding of enzyme-polymer interfacial interactions. Often shorter chain substrate analogs have been used in interaction studies and modeling, which fail to accurately mimic the real PET substrates. Recent efforts in computational modeling of bulk substrate and its surfaces offer promising avenues to elucidate these protein-polymer interactions and guide the rational design of more effective biocatalysts (Jäckering *et al.* 2024).

Building on current insights into PETases, multiple routes can and should be explored to advance enzymatic plastic degradation. Alternative processes yielding educts for different compounds or utilizing non-aqueous media to make polymer chains more accessible may offer more viable solutions than current recycling efforts. To fully exploit these strategies, it is important to further diversify the enzyme portfolio through engineering, e.g. Kubu and Mipa (Seo *et al.* 2025), or the identification of enzymes from (potentially) extremophile organisms (Turak *et al.* 2025) allowing less conventional reaction conditions to be employed. Standardization of substrate, evaluation, and reporting is critical to enable meaningful comparisons between new enzymes and connected processes (Wei *et al.* 2025). To broaden the impact of enzyme engineering for plastic degradation, other polymers such as the difficult to degrade polyolefins, have to be targeted, and the key lessons from PETase research need to be incorporated to accelerate progress and successes. This includes a deeper connection between material and enzyme engineering to find the optimal conditions for breaking down existing polymers or engineering novel and easier degradable polymers alongside their tailored enzyme counterparts.

### Biomass processing (Anikó Várnai and Vincent G.H. Eijsink^*^)

**Figure 20:**
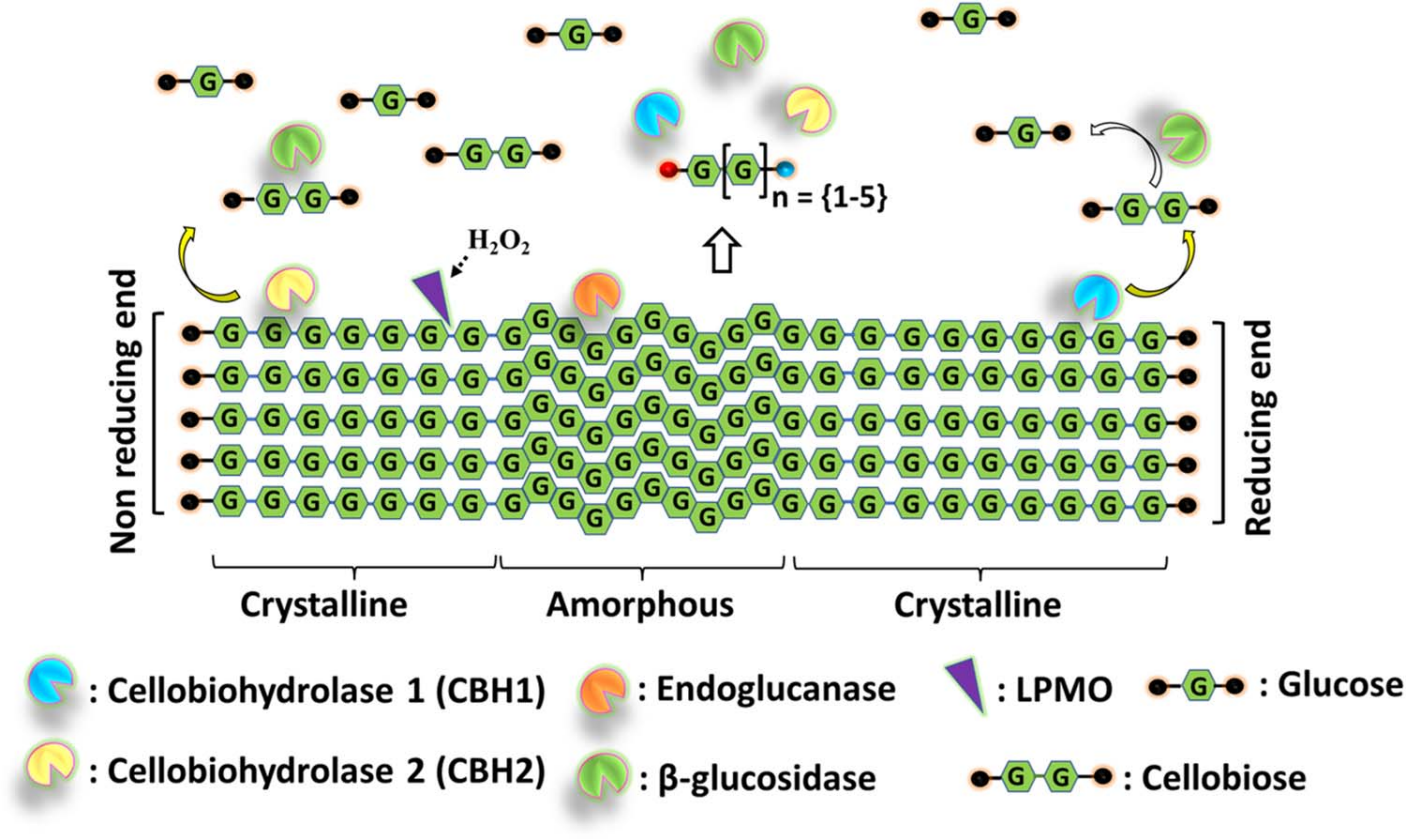
Enzymes involved in the degradation of cellulose. Different cellulase types, with different names, act on different parts of the cellulose fiber; e.g. processive cellobiohydrolases act from chain ends in crystalline regions, and endoglucanases act on more amorphous regions. LPMOs use oxidative chemistry to introduce chain breaks in crystalline regions, thus making these regions more accessible. Soluble products released by LPMOs will mostly be 4–8 sugars long and will be oxidized at one of the chain ends, as indicated by the coloured dots. This picture was taken from Chaudhari *et al.* (2023). A typical enzymatic system for depolymerization of chitin looks similar (Vaaje-Kolstad *et al.* 2013).

Enzymatic valorization of biomass, in particular non-edible biomass, has received much attention in recent decades and has stimulated massive efforts in enzyme discovery and engineering, both in academia and industry. Most research in this area has been devoted to unraveling and engineering the complex enzyme systems (Fig. 20) (Østby *et al.* 2020) that are needed to depolymerize abundantly available insoluble homopolymeric polysaccharides in biomass, in particular cellulose in straw and other plant materials, and, to a lesser extent, chitin in crustacean shells, and fungal cell walls. Depolymerization is achieved by the combined action of hydrolases (cellulases (Payne *et al.* 2015) and chitinases (Vaaje-Kolstad *et al.* 2013)) and recently discovered redox enzymes called lytic polysaccharide monooxygenases (LPMOs) (Horn *et al.* 2012; Bissaro and Eijsink 2023).

Using cellulases as an example, and looking at it from an industrial perspective, multiple protein engineering targets have been pursued and reached, yielding enzymes with improved stability and catalytic activity over a specific operational pH range, and enzymes showing reduced product inhibition or reduced non-productive adsorption to the substrate (Chaudhari *et al.* 2023). The ease (cost) of production is another important selection and engineering target. Early engineering work was mainly focused on understanding cellulase function, including pioneering studies on cellulases from *Trichoderma reesei* and *Thermobifida fusca* (Schülein 2000; Wilson 2009; Lantz *et al.* 2010). As to improving cellulase properties, engineering of stability and activity has been most visible in the literature, starting with notable early examples (Heinzelman *et al.* 2009; Lantz *et al.* 2010; Taylor *et al.* 2018). Today’s efficient commercial cellulase cocktails are the result of decades of enzyme discovery followed by tailoring of enzyme properties using protein engineering technologies (Harris *et al.* 2014; Østby *et al.* 2020). This development would not have been possible without the generation of large mutant libraries and using realistic (i.e. insoluble) substrates for screening (Lantz *et al.* 2010). The emergence of these complex cocktails is a major achievement in industrial enzymology, since success in this area has required the parallel optimization of multiple enzymes. Of note, much of the underlying enzyme engineering work has been done by commercial enzyme suppliers and is largely unknown to the scientific community. It should also be noted that publicly available engineering studies often employ substrates and reaction conditions that are not industrially relevant.

Protein engineering has played a crucial role in unravelling the structural determinants of the performance of processive exo-acting hydrolases, which are key players in the enzymatic conversion of chitin and cellulose (Vaaje-Kolstad *et al.* 2013; Payne *et al.* 2015). These are complex enzymes, with substrate-binding clefts or tunnels that are lined with aromatic residues and that are capable of simultaneously interacting with multiple sugar units in the polysaccharide chain. Engineering studies, by several groups and on several enzymes, have revealed structural determinants of substrate binding and processivity. Importantly, these studies have revealed a trade-off between strong binding, which is needed for processive action and activity on crystalline material, and catalytic rate, which is hampered by the slow off-rates associated with strong binding (Horn *et al.* 2006; Igarashi *et al.* 2011; Kurasin and Väljamäe 2011; Kari *et al.* 2014, 2018; Schaller *et al.* 2022). High substrate binding also brings risks of product inhibition and of non-productive binding to the substrate. Clearly, substrate and product affinity need to be carefully tuned and are major targets in cellulase and chitinase engineering. Of note, manipulation of substrate affinity by appending or deleting carbohydrate-binding modules in cellulases is a popular engineering strategy. However, the value of this strategy for industrial biomass processing remains somewhat unclear (Cruys-Bagger *et al.* 2013; Várnai *et al*. 2013; Chaudhari *et al.* 2023).

Further development of enzymes for biomass processing will undoubtedly benefit from the rapid development of AI-based tools for enzyme (re-)design. However, as pointed out above, the optimization challenge is daunting, because of particular features of interfacial catalysis (Kari *et al.* 2018) and the need for multiple enzymes working together in an optimal manner. Little is known about how the contribution of LPMOs may be optimized (Østby *et al.* 2023). Interestingly, these crucial enzymes pose a somewhat unusual, and hitherto hardly explored, engineering challenge, since their ability to withstand autocatalytic oxidative inactivation needs to be improved to increase industrial performance, e.g. by tailoring protective hole hopping routes (Ayuso-Fernández *et al.* 2024). Despite major progress (Igarashi *et al.* 2011; Jalak *et al.* 2012; Vaaje-Kolstad *et al.* 2013; Payne *et al.* 2015; Zajki-Zechmeister *et al*. 2022; Sørlie *et al*. 2023), better understanding of the interplay between the various enzyme types is still much needed to drive future optimization of individual enzymes and enzyme cocktails. Importantly, this interplay will vary with the nature of the substrate (e.g. crystallinity) and will change during enzyme treatment of those substrates (Olsen *et al*. 2017; Hamre *et al.* 2019) adding an additional level of complexity to rational design of improved biomass-converting enzymes.

## Antibodies and therapeutics

### Engineered antibodies for oncology applications (Sacha Zinn and Daniel Christ^*^)

**Figure 21:**
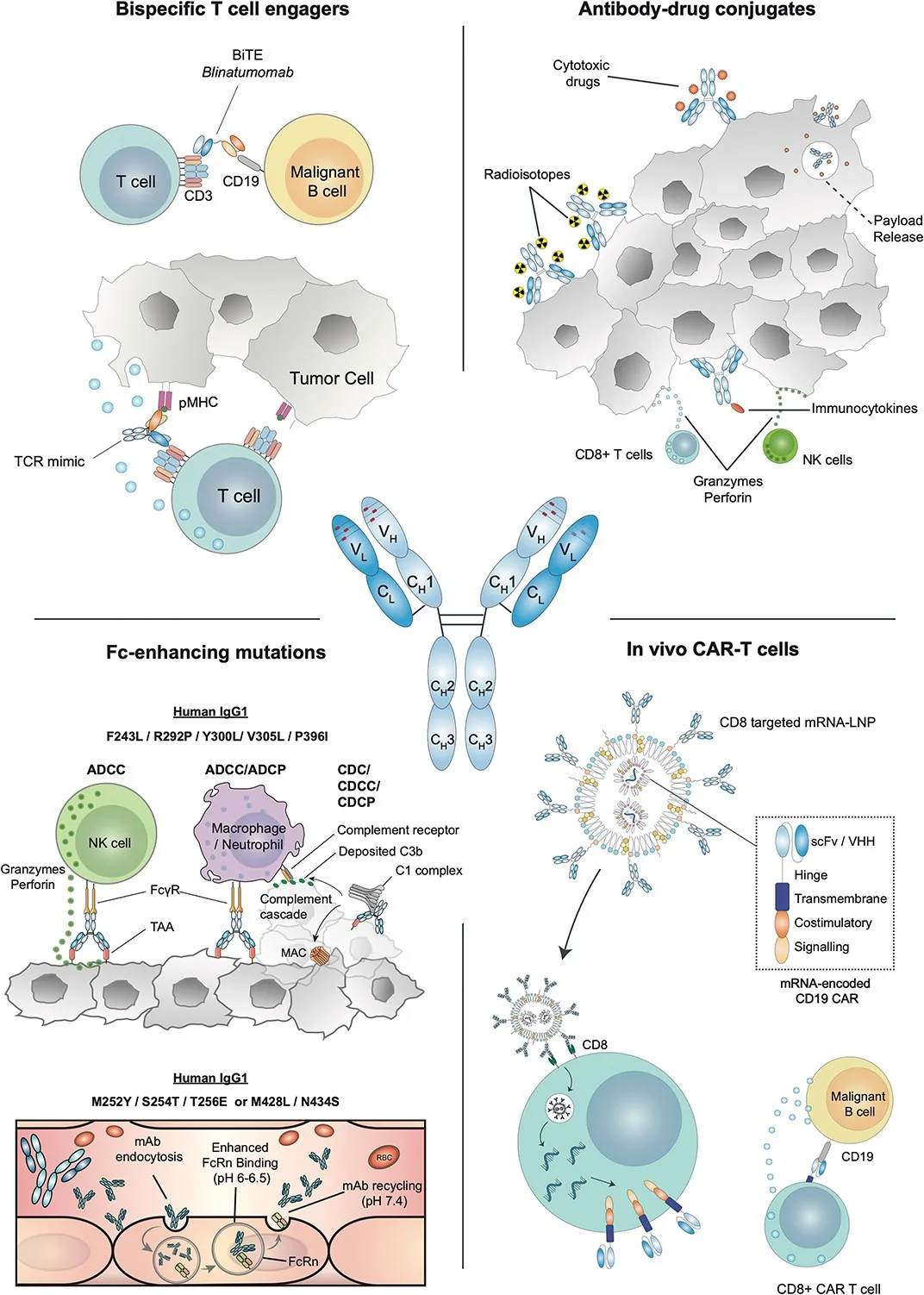
Antibodies and antibody-derived agents in the treatment of cancer. *Center panel*: Schematic of an engineered full-length IgG1 monoclonal antibody (150 kDa). *Top-left panel (therapeutic modality)*: T cell engagers (TCEs) are typically bispecific molecules directed against tumor associated antigens (TAAs) and agonistic T cell surface receptors (e.g. CD3). TCR-mimics target cancer-associated pMHC (e.g. KRAS G12V), allowing for intracellular proteome targets to now be effectively investigated (Johansen et al. 2025a; Skrzypczynska, et al. 2025). *Top-right panel (therapeutic modality)*: Cytotoxic-drug conjugates comprise the vast majority of clinically approved ADCs (Staudacher and Brown 2017; Fu *et al.* 2022; Woodford *et al.* 2025), while radioisotope-conjugates and immunocytokines are both maturing technologies that demonstrate unique potential in anti-tumor activity (Yamaguchi *et al.* 2022; Rotta *et al.* 2024; Santollani *et al.* 2024). *Bottom-left panel (recent advances)*: Fc-engineering for enhancement of effector functions typically aims to increase affinity for FcγR and C1q to improve antibody-dependent cellular cytotoxicity (ADCC), antibody-dependent cellular phagocytosis (ADCP), and complement-dependent cytotoxicity (CDC) activity against tumors (van der Horst *et al.* 2020; Hale *et al*. 2024). FcRn enhancing mutations improving mAb recycling have been adopted in clinically approved mAbs, leading to significant improvements in serum half-lives (Ramdani *et al.* 2022; Ko *et al.* 2025). *Bottom-right panel (recent advances)*: Conjugation of antibodies to mRNA-LNPs confers targeting properties allowing for transfection of specific cell types (Bui *et al.* 2024). CD8-targeting LNPs (tLNPs) encoding an anti-CD19 CAR have shown success in the generation of active CAR T cells in pre-clinical studies (Hunter *et al.* 2025).

With the approval of rituximab in 1997 now past its 25-year anniversary (Grillo-López *et al.* 1999), monoclonal antibodies (mAbs) have become a mainstay of cancer therapy (Zinn *et al.* 2023). Since the pioneering of hybridoma technology by Kohler and Milstein in 1975 (Köhler and Milstein 1975), the therapeutic limitations of murine-derived antibodies quickly became apparent and the first chimeric human antibodies were developed in 1984 (Boulianne *et al*. 1984; Morrison *et al.* 1984). This was quickly followed by the discovery of antibody humanization in 1986 by Winter and colleagues (Jones *et al.* 1986a). In 1987, it was first demonstrated that protein engineering could be used to enhance the affinity of an antibody for its antigen (Roberts *et al*. 1987) and in 1990 the first fully human monoclonal antibodies would arise from phage-display systems (McCafferty *et al.* 1990b). These antibody engineering strategies, including humanization, affinity maturation, developability optimization, and immunogenicity reduction have been essential in the overwhelming success of antibodies as therapeutic agents (Jones *et al.* 1986b; Riechmann *et al.* 1988; Lowe *et al.* 2011; Dudgeon *et al.* 2012; Rouet *et al.* 2012; Liu *et al.* 2020). More recently, Fc engineering, glycoengineering, and structural optimization have become important strategies to enhance antibody safety and potency, forming the basis of modern engineered antibody platforms (Fig. 21).

Immune checkpoint inhibitors, including antibodies targeting CTLA-4, PD-1, and PD-L1, represent transformative advances in cancer immunotherapy, with the potential of producing durable responses across select tumor types (Lee *et al.* 2016; Waldman *et al*. 2020). Animal models and the analysis of patient polymorphisms have highlighted the central role of Fcγ receptor biology in therapeutic efficacy (Clynes *et al.* 2000; Weng and Levy 2003; Musolino *et al.* 2008). Antibody–drug conjugates (ADCs) constitute a therapeutic breakthrough by merging antibody specificity with potent cytotoxic payloads (Fu *et al.* 2022). Advances in payload chemistry, linker design, and site-specific conjugation have driven the success of clinically validated ADCs such as trastuzumab emtansine, brentuximab vedotin, and trastuzumab deruxtecan (Chari *et al*. 2014; Dott *et al*. 2018; Woodford *et al.* 2025). These therapeutics highlight the complex mechanics of target internalization, lysosomal processing, and payload release in achieving a high therapeutic index (Staudacher and Brown 2017). Bispecific T-cell engagers, including the fragment-based blinatumomab (Burt *et al*. 2019), enable potent cytotoxicity by recruiting CD8+ effector T cells directly to targeted cancer antigens (Douglass *et al.* 2021). Radioimmunoconjugates, including targeted radionuclide therapies such as ^177^Lu-PSMA-617 (Pluvicto), have enabled systemic delivery of tumor-localized radiation (Sartor *et al.* 2021).

Current areas of innovation focus on enhancing selectivity, expanding target scope, and improving therapeutic windows (Vazquez-Lombardi *et al.* 2015; Zinn *et al.* 2023; Rotta *et al.* 2024). Masked antibody formats with engineered protease-cleavable sites aim to reduce systemic toxicity and broaden therapeutic potential (Ratcliff *et al.* 2021; Orozco *et al.* 2022). Alternative antibody isotypes, as well as engineering of the antibody Fc region (van der Horst *et al.* 2020; Hale *et al*. 2024) are being explored for ability to engage new effector cell populations, potentially unlocking antitumor activities not accessible to the human IgG1 (and IgG4) isotypes that dominate current antibody therapeutics (Vukovic *et al.* 2021). T cell receptor mimic antibodies enable the targeting of intracellular antigens *via* recognition of peptide–major histocompatibility complexes (pMHCs), with binder specificity, MHC diversity and target selection all remaining key engineering challenges (Douglass *et al.* 2021; Wright *et al.* 2023). Advances in ADC technology, including novel payloads, promise to limit resistance mechanisms and enhance efficacy (Chari *et al*. 2014; Fu *et al.* 2022; Woodford *et al.* 2025). Cytokine-armed antibodies, co-stimulatory receptor agonists, multivalent bispecifics, as well as lipid-nanoparticle (LNP) conjugated and/or LNP-encoded antibodies provide promising new approaches (Fig. 21) and position engineered antibodies at the forefront of next-generation cancer therapeutics (Waldman *et al*. 2020; Douglass *et al.* 2021; Bui *et al.* 2024; Hunter *et al.* 2025).

### Bispecific antibodies (Oliver Seifert and Roland E. Kontermann^*^)

The concept of bispecific antibodies (bsAbs) originated in the 1960s when researchers first demonstrated that the antibodies of mixed specificity can be obtained by recombination of a mixture of univalent antibody fragments (Nisonoff and Rivers 1961). However, practical development began in the 1980s with the advent of the hybridoma technology, which enabled the production of monoclonal antibodies and inspired scientists to engineer dual-specific constructs by methods such as chemical conjugation of two antibodies or fusing two hybridoma into a quadroma, producing, among other species, bispecific antibodies (Milstein and Cuello 1983). Subsequently, advances in recombinant DNA technology allowed for genetic fusion of antibody fragments, leading to more defined formats (Riethmüller 2012). Central to this engineering is the use of building blocks, including scFv, single-domain antibodies, and Fab fragments, which can be further combined with Fc-regions, as well as derivatives thereof designed to facilitate cognate light chain plus heavy chain pairing and to allow for the formation of heterodimeric heavy chains (Madsen *et al.* 2024). This has led to an entire zoo of more than 100 different formats with varying valency and geometry (Brinkmann and Kontermann 2017). The guiding principle behind bsAbs is to harness the antibody with the ability to simultaneously or consecutively bind two distinct targets to enhance therapeutic specificity and efficacy while maintaining drug-like properties.

**Figure 22:**
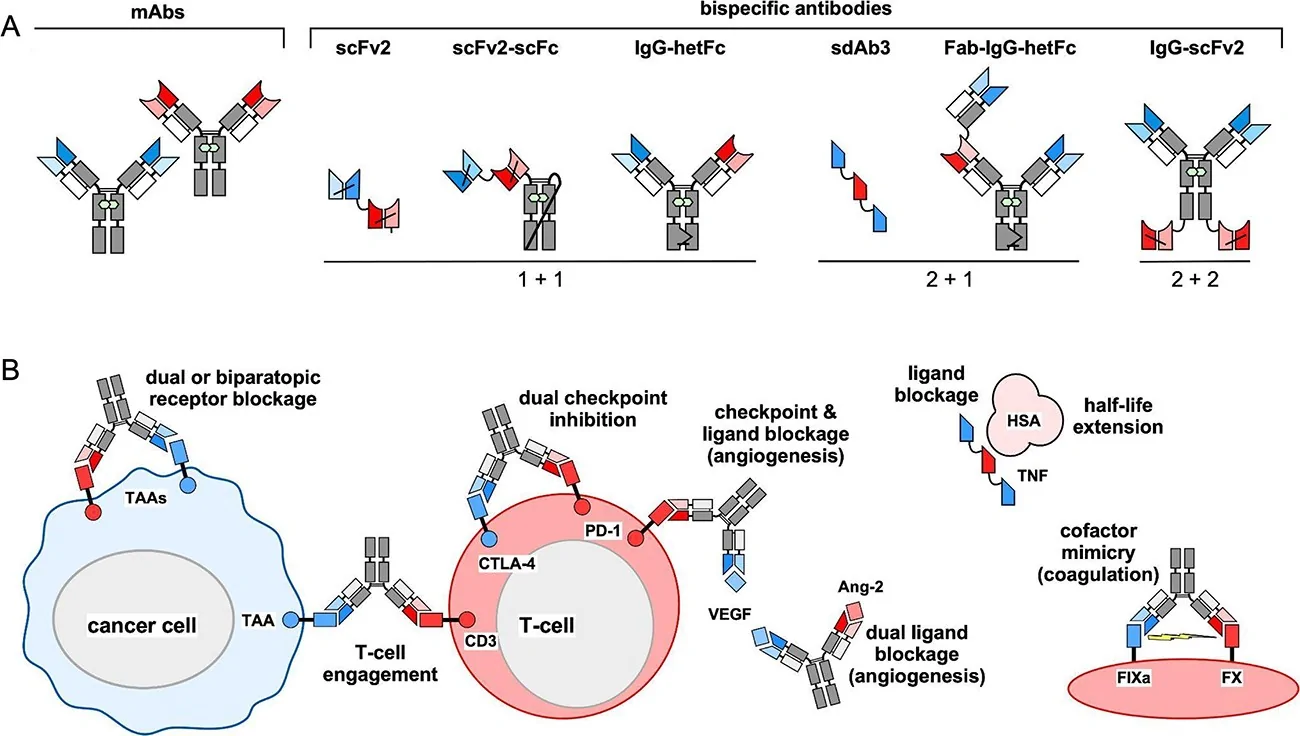
Structures and applications of bispecific antibodies. (A) Examples of various formats of bispecific antibodies with different valencies approved for therapy, from left to right: Tandem scFv (scFv2), tandem scFv-single-chain Fc (scFv2-scFc), IgG-hetFc, triple single-domain antibody (sdAb3), fab-IgG-hetFc, IgG-scFv2. (B) Therapeutic applications of bispecific antibodies in cancer and non-cancer indications.

The current state of the field is characterized by rapid growth, with over 360 bsAbs currently in clinical development, exploring diverse targets and mechanisms (Brinkmann and Kontermann 2026). Approaches are dominated by T-cell engagement and immune checkpoint modulation, with additional mode-of actions being covered such as cytokine redirection, dual-pathway inhibition, and drug delivery (Fig. 22) (Nie *et al.* 2020). As of now, 20 bsAbs have been approved for therapeutic applications, most of them for cancer therapy, and a few for non-cancer indications (Klein *et al.* 2024b). Representative examples of bsAbs highlight the field’s evolution from conceptual innovation to clinical success. Catumaxomab, a quadroma-derived bsAb redirecting T-cells to epithelial-cell adhesion molecule (EpCAM)-expressing tumor cells, was the first therapeutic bsAb, approved 2009 in Europe. The first genetically engineered bsAbs, approved in 2014, was blinatumomab, a bispecific T-cell engager (BiTE) that engages T-cells and CD19-expressing B-cell malignancies. Another major achievement is emicizumab, which bridges activated factor IX and factor X, restoring coagulation in hemophilia A patients, an example of bsAb application beyond oncology. More recent innovations include amivantamab for EGFR- and MET-driven non-small cell lung cancer and teclistamab for multiple myeloma, both demonstrating the versatility of bsAbs across cancer types. Of note, more than 140 bsAbs studied in clinical trials have been terminated or were not further pursuit, often due to lack of efficacy, safety issues, or for business reasons, highlighting that there is still room for improvements and considerations of the best target combinations (Brinkmann and Kontermann 2026).

Emerging trends in the field of bsAbs are focused on Fc engineering for modulation of pharmacokinetics, stability, and Fc-mediated effector functions, and improving therapeutic precision and safety by engineered bsAbs with tunable affinities and conditional activation mechanisms, such as prodrug or tumor microenvironment-activated designs, to minimize on-target off-tumor effects and systemic toxicity. Novel formats like trispecific and multispecific antibodies extend bsAb principles to engage multiple pathways simultaneously, enhancing immune modulation and overcoming resistance (Goebeler *et al*. 2024). Additionally, combination strategies integrating bsAbs with CAR-T cells, checkpoint inhibitors, or cytokine therapies are showing synergistic efficacy. The field is also moving toward off-the-shelf modular platforms and gene-delivered bsAbs using viral vectors or mRNA, offering more flexible and durable treatment options. Collectively, these innovations are shaping a new era of smarter, safer, and more effective antibody therapeutics, making bsAbs a cornerstone of next-generation immunotherapy.

### Single domain antibodies (Fang Zheng and Serge Muyldermans^*^)

**Figure 23:**
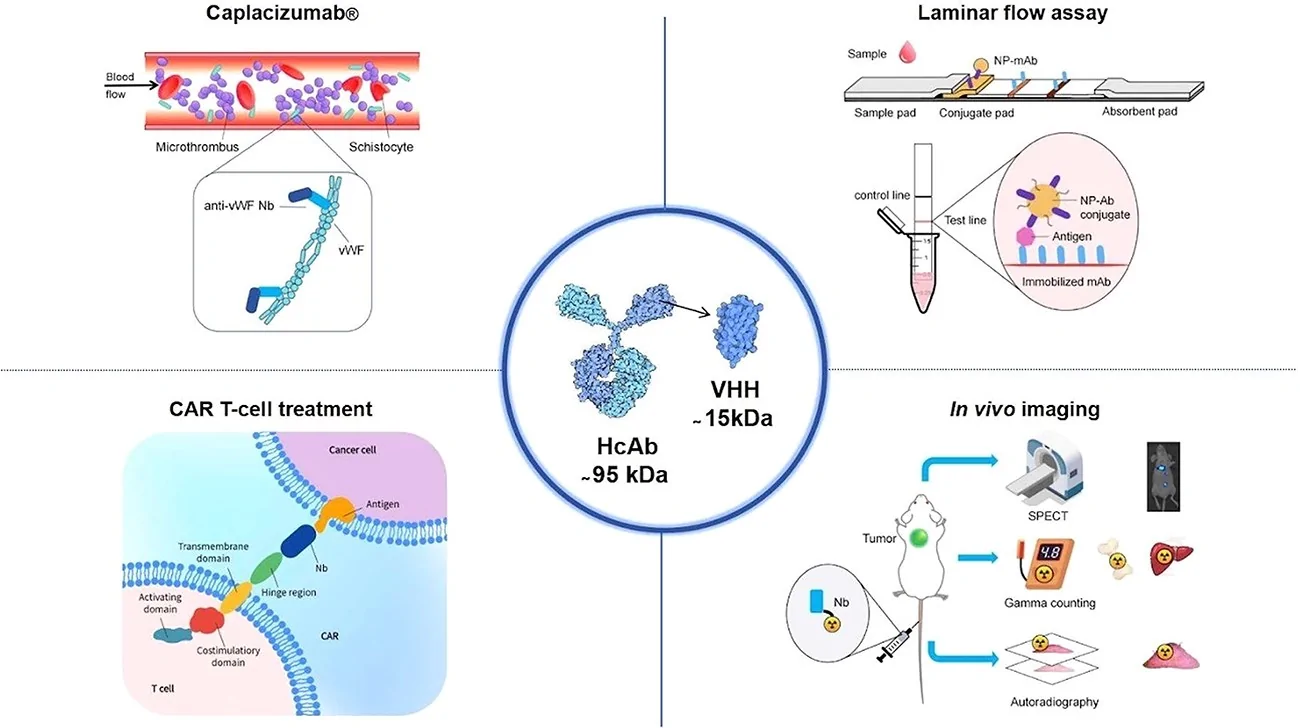
Applications of nanobodies in therapy and diagnosis. *Center panel*: Structures and molecular weights of HcAb (~95 kDa) and VHH (~15 kDa, PDB ID 7DJY) (Wen and Zheng 2020). HcAb representation is adapted from Goodsell (Goodsell 2011). *Top-left panel*: The therapeutic Caplacizumab® is an anti-vWF nanobody that inhibits microthrombosis by binding vWF, reducing microthrombi and schistocytes. *Bottom-left panel*: CAR T-cell therapies can employ nanobodies to enable T cells to target cancer cell antigens. *Top-right panel*: Nanobody-based lateral flow assays can be used in diagnostic applications to detect antigens *via* NP-Ab conjugate and immobilized mAb. *Bottom-right panel*: Radiolabeled nanobodies can enable *in vivo* tumor imaging *via* SPECT, gamma counting, and autoradiography.

The development of single-domain antibodies (sdAbs) followed the serendipitous discovery of functional heavy chain-only antibodies in camelids (camels, dromedaries, llamas, alpacas) (Hamers-Casterman *et al.* 1993). These homodimeric antibodies recognize antigens through their single variable domains, referred to as the variable domain of the heavy chain of heavy-chain-only antibodies (VHH) (Fig. 23). Researchers initiated sdAb generation by immunizing camelids with target antigens, cloning the VHH repertoire from peripheral blood lymphocytes, and isolating antigen-specific binders using phage display (see Section *In vitro* display (Takuya Terai and Naoto Nemoto^*^)) (Arbabi Ghahroudi *et al.* 1997). In addition to immune libraries, large naïve and synthetic libraries—where codons of antigen-binding loops are randomized—have been established as valuable alternatives when immunization is impractical or ethically restricted (Muyldermans 2021). Recent progress in ML and AI has enabled the *in silico* design of sdAbs with predefined epitope specificity and moderate to high affinity (Mille-Fragoso *et al.* 2025). VHHs express efficiently in microbial hosts, exhibit high solubility, and are structurally stable and robust. Importantly, they share substantial sequence identity with human VH domains, supporting their low immunogenicity (Ackaert *et al.* 2021) and therapeutic potential.

High-affinity, antigen-specific VHHs, also referred to as Nanobodies®, can typically be isolated within two months following immunization. These Nanobodies® have found wide application in research, diagnostics, and therapy (Yu *et al.* 2024). In structural biology, Nanobodies® act as crystallization chaperones, facilitating the structure determination of otherwise recalcitrant proteins. The GFP-specific Nanobody® is among the most widely used reagents for capturing GFP-tagged proteins (Rothbauer *et al.* 2008). When expressed intracellularly, Nanobodies® remain stable and functional, serving as intrabodies targeting proteins inside the cell (Rothbauer *et al.* 2006). Gene fusion of a target-specific Nanobody and GFP or mRFP (a Chromobody), followed by expression in mammalian cells, enables real-time visualization of antigen localization and trafficking within living cells. Nanobodies® are cost-effective to produce and easy to conjugate with enzymes such as horseradish peroxidase or alkaline phosphatase, forming the foundation of numerous ELISA and lateral-flow assays (Yu *et al.* 2024). Their small molecular size—below the renal filtration threshold—and compatibility with various radiolabeling methods allow their use as non-invasive imaging probes in oncology. Currently, four Nanobody®-based therapeutics have received regulatory approval: Cablivi, Carvykti, Nanozora, and Anweida (Jovčevska and Muyldermans 2020).

Nanobodies are expected to remain indispensable tools in structural biology, particularly for X-ray crystallography and cryo-EM. As Chromobodies, they will enable dynamic imaging in cell and developmental biology. Radiolabeled Nanobodies® are emerging as promising theranostic agents in oncology. Furthermore, enzyme-inhibiting Nanobodies® may serve as novel immunomodulators for treating diverse diseases, including inflammatory conditions and viral infections. Protein engineering strategies are also being employed to broaden their reactivity against viral escape mutants and to target challenging membrane proteins such as GPCRs (Kalogriopoulos *et al.* 2025). Integration of Nanobodies® into CAR-T platforms is anticipated to become an expanding frontier in targeted immunotherapy (Jovčevska and Muyldermans 2020). Finally, their ability to cross the blood–brain barrier opens up new possibilities for treating neurological disorders (Zheng *et al.* 2022).

### Alternative scaffolds as specific binding proteins (Andreas Plückthun^*^)

For decades, monoclonal antibodies were generated through the immunization of animals. Advances in sequencing their genes and structural characterization enabled the design and construction of semisynthetic and synthetic DNA libraries and the development of display technologies, allowing antibody fragments to be selected for specific target binding without the need for immunization (Winter and Milstein 1991; Knappik *et al.* 2000). Once these approaches were established, the antibody scaffold itself was no longer essential—virtually any protein could, in principle, be engineered into a library with randomized surfaces and/or loops. The pursuit of alternative scaffolds was driven largely by the limitations of antibody fragments, including their reliance on disulfide bonds (which restrict efficient expression in the cytoplasm) and their β-sandwich architecture (which can promote aggregation) (Wörn and Plückthun 2001). Still, the high affinities and specificities achieved by recombinant antibody fragments set a demanding benchmark that all alternative scaffolds were required to surpass.

**Figure 24:**
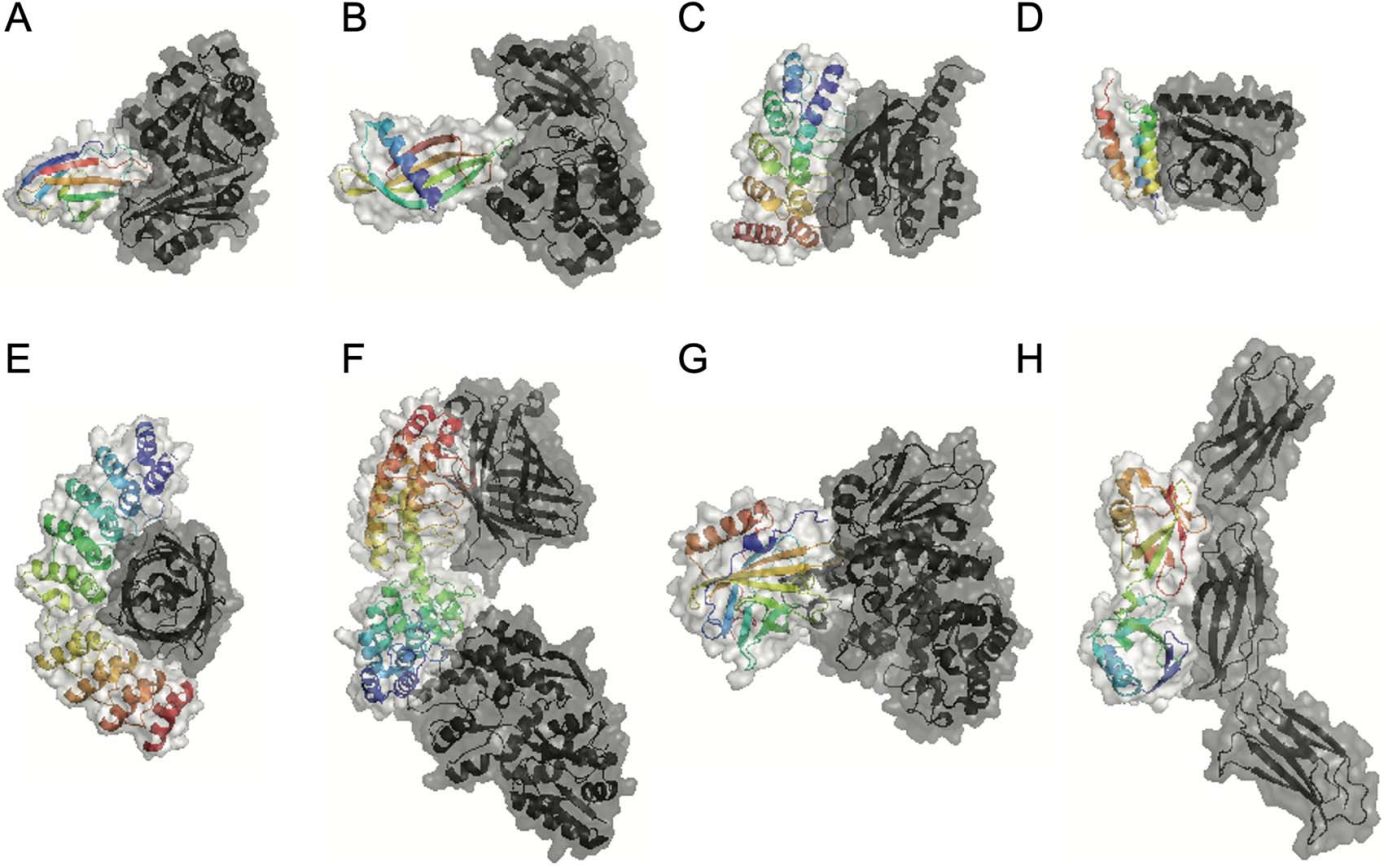
Exemplary structures of scaffold proteins with their cognate targets. These structures illustrate the different modes of interaction (loops and/or surfaces) and different architectures (mainly α-helical, mainly β-sheet, or mixed). In all cases, the binding protein is shown as cartoon in rainbow coloring from its N- to C-terminus, with a transparent light grey surface, while the target is shown as dark grey cartoon with dark grey surface. The originating domain and common names are mentioned. (A) Fibronectin type-III domain (Monobody, Adnectin, Centyrin) (PDB ID 8GZX) (Fujita *et al.* 2023), (B) Cystatin-A (Affimer) (PDB ID 8C12) (Martin *et al.* 2023), (C) Designed Ankyrin Repeat Protein (DARPin) (PDB ID 9GTK) (Kapp *et al.* 2025), (D) IgG-binding domain of protein A (Affibody) (PDB ID 5DJU) (Wang *et al.* 2016; Zhang and Zhang 2024), (E) linked DARPins wrapping around a target (PDB ID 5MA5) (Hansen *et al.* 2017), (F) rigidly fused DARPins engaging two targets (PDB ID 5LEM) (Wu *et al*. 2017b), (G) lipocalin (Anticalin) (PDB ID 6SUA) (Deuschle *et al.* 2020), and (H) ubiquitin or γ-B-crystallin (Affilin) (PDB ID 8PEQ) (Katzschmann *et al.* 2024).

The scaffolds have been generally chosen based on properties that favor engineering (Gebauer and Skerra 2020): their native role in protein–protein interactions, compact single-domain architecture, and high intrinsic stability. In some cases, antibody-mimetic designs were pursued by introducing randomized loops to serve as binding regions (Fig. 24). Other scaffolds exploit surfaces, such as α-helical arrays or β-sheet surfaces, as binding interfaces. Repeat proteins further extend this concept by enabling modular tuning of contiguous interaction surface size (Plückthun 2015; Stark *et al.* 2024). While most scaffolds originated from a single chosen natural backbone with randomized segments, an alternative strategy was to leverage consensus design (Porebski and Buckle 2016): sequence alignments of homologous family members are used to select the most frequent residue at each position, yielding highly stable consensus frameworks. Designed Ankyrin Repeat Proteins (DARPins, binding to folded domains) (Plückthun 2015) and Armadillo Repeat Proteins (modular binding to linear epitopes) (Stark *et al.* 2024) exemplify this approach. Only the contiguous helix surfaces and loops were diversified, ensuring very robust folding and stability. This focus on highly stable library scaffolds, achieved both through the use of stable underlying architectures and consensus framework design, addresses the inherent tension between function and stability. Specific binding requires particular surface residues, often in close proximity on the scaffold surface, and these can be destabilizing. Starting from a highly stable consensus scaffold therefore permits substantial stability loss during engineering for binding while still yielding a stable, specific binder.

Progress in synthetic library technologies—such as trinucleotide-based synthesis to eliminate stop codons and to control mixed codon bias (Virnekäs *et al.* 1994)—combined with powerful selection and directed evolution platforms (ribosome, phage, and yeast display) (Lipovsek and Plückthun 2004; Mahdavi *et al.* 2022) has driven scaffold binder development. Resulting affinities and specificities were thus frequently comparable to antibodies, and binders have been applied in many preclinical models, particularly for tumor targeting and imaging. Notably, their small size and disulfide-independent folding have enabled unique intracellular applications under reducing conditions, including live-cell imaging, intracellular inhibition, and transcriptional modulation. Another application has been their use in structural biology, either as a chaperone or in forming host-guest crystals (Mittl *et al*. 2020).

Recent progress in *ab initio* protein design is beginning to shift the paradigm of library construction and selection (Krishna *et al.* 2024; Butcher *et al.* 2025). Instead of starting with one scaffold, one can begin with a defined epitope, define a complementary binding patch, and subsequently build a scaffold around it. However at present, this strategy still requires large numbers of parallel designs and extensive experimental testing, and it does not yet consistently deliver faster development or higher affinities, nor is it directly testing for specificities. Since AI-based design methods are primarily trained on geometry, that is, on structures present in the PDB, the geometric accuracy of the resulting designs can be spectacular. Interestingly, this is often not accompanied by exceptionally high affinity, at least not at the level currently achievable through directed evolution, highlighting that affinity itself is not part of the training set. Affinity data can also be problematic because they are highly dependent on experimental methods and parameters, each prone to error and often of unclear generalizability. Furthermore, energetic contributions from factors such as residual entropy in the bound state, solvent effects, and conformational changes in either binding partner are often ignored, and the field will likely need to incorporate more sophisticated models of binding in the future. Nevertheless, given the rapid pace of innovation in computational protein design, it is reasonable to expect continued progress in this direction.

A major opportunity—and driving force—in this field is the development of specific binding proteins for therapeutic applications. Many of these alternative scaffold molecules have already advanced into clinical trials, including some with highly sophisticated mechanisms of action that demand multiple domains and architectures not easily achievable with antibodies (Stumpp *et al.* 2025). Despite these advances, immunogenicity remains a significant challenge for engineered scaffolds, *de novo* designed binding proteins and antibodies themselves (De Groot *et al.* 2023). Current mitigation strategies are largely empirical, and the development of generalizable solutions to reduce or eliminate immunogenicity would provide a transformative boost to the field.

### Cytokines and growth factors (Lauren O. Chisholm and Jamie B. Spangler^*^)

One extraordinarily active area in the field of molecular engineering has been the design and manipulation of secreted proteins such as cytokines and growth factors. As cytokines and growth factors play crucial roles in human physiology by regulating cell growth, migration, signaling, differentiation, and survival, these molecules harbor tremendous potential as therapeutics. However, the clinical performance of cytokines and growth factors has been limited by several intrinsic properties of these proteins, including: (i) pleiotropic and often contradictory activities, which hamper efficacy and lead to off-target toxicities; (ii) redundancy of functions, which complicates inhibition of protein activities; (iii) difficulties in recombinant expression, which impede biomanufacturing; and (iv) short half-life in the bloodstream, which leads to transient responses and reduced therapeutic window (Moraga *et al.* 2014; Spangler *et al.* 2015; Ren *et al.* 2019; Saxton *et al*. 2023; An *et al.* 2024a). Protein engineering offers the exciting opportunity to overcome these challenges by increasing the specificity, stability, and *in vivo* persistence of cytokines and growth factors through various approaches that involve either direct modification of the protein, conjugation/fusion to another molecule, or both. In addition, the function of cytokines and growth factors can be antagonized by engineering binding molecules, for instance receptor decoy proteins, that target these proteins and block their activity. Structural insights have been pivotal for informing the rational design of engineered cytokines and growth factors. Moreover, experimental techniques, such as site-specific mutagenesis (Cunningham *et al*. 1990; Cunningham and Wells 1991) and directed evolution (Smith 1985; Boder and Wittrup 1997; Zhou *et al.* 2010), as well as computational approaches, such as ML (Jumper *et al.* 2021) and *de novo* design (Huang *et al*. 2016c), have monumentally advanced our ability to create customized cytokines and growth factors for a vast range of biomedical applications.

Seminal work by Wells and colleagues launched the field of cytokine and growth factor engineering in 1990 by grafting receptor-binding residues from human growth hormone into the distantly related hormone prolactin, creating a variant of prolactin that bound to human growth hormone receptor with >10 000-fold improved affinity compared to the native protein (Cunningham *et al*. 1990). Subsequent work extended this concept to rationally design a variant of human growth hormone that reversed its receptor binding bias from human growth hormone receptor to prolactin receptor (Cunningham and Wells 1991). These early proof-of-concept studies set the stage for future efforts in cytokines and growth factor engineering, which continue to evolve as new technologies for design and characterization are developed.

**Figure 25:**
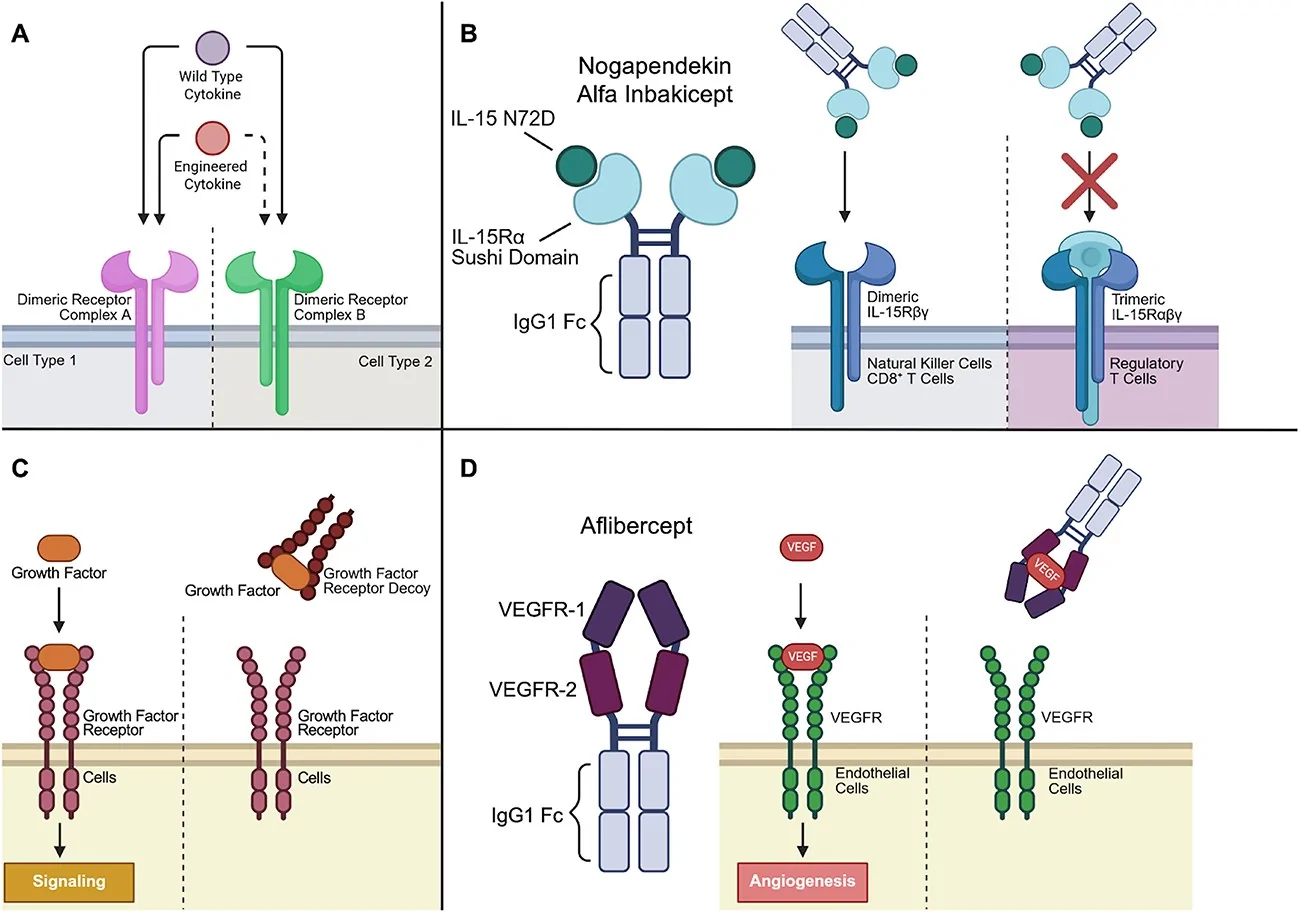
Engineered cytokines and growth factors. (A) Generic example of a cytokine that has been engineered to modulate receptor specificity. The wild type cytokine activates receptor complex A and receptor complex B equally, whereas the engineered cytokine preferentially activates receptor complex A over receptor complex B. The two receptor complexes are expressed by different cell types, thus resulting in activation of cell type 1 *vs.* cell type 2 when the engineered cytokine is present. (B) Schematic depicting the FDA approved engineered cytokine therapeutic nogapendekin alfa inbakicept, which consists of an IL-15 variant (N72D) engineered for superagonist activity combined with the IL-15Rα sushi domain fused to a human IgG1 Fc. The presence of the IL-15Rα sushi domain prevents nogapendekin alfa inbakicept from activating the trimeric IL-15Rαβγ complex, resulting in proliferation of natural killer cells and CD8^+^ T cells, but not regulatory T cells. (C) Generic example of a growth factor receptor decoy protein. When the growth factor receptor decoy is present, it binds to and sequesters the growth factor, preventing receptor activation. (D) Schematic depicting the FDA approved VEGF receptor decoy therapeutic aflibercept, which consists of two VEGF receptor domains fused to a human IgG1 fc domain. When aflibercept is present, circulating VEGF binds to aflibercept, precluding activation of VEGFR and, consequently, preventing angiogenesis. Created in BioRender. Chisholm (2025). https://BioRender.com/vjzxzez.

Fast forward to today, the field of engineering cytokines and growth factors as therapeutics is thriving. Cytokine engineering efforts have largely focused on either enhancing or disrupting the cytokine-receptor interface, with some efforts to redirect, expand, or narrow receptor specificity (Fig. 25A) (Spangler *et al.* 2015). These engineered cytokines have applications in a vast array of disease conditions, ranging from cancer to autoimmune disorders, with several engineered variants entering clinical trials (O’Neal *et al.* 2026). Though engineered cytokine and growth factor therapeutics are not as clinically advanced as antibody-based therapeutics, efforts to engineer interleukins, in particular interleukin-2 (IL-2) and IL-15, are amongst the most developed. The only engineered cytokine currently approved by the FDA is nogapendekin alfa inbakicept (Anktiva®, ImmunityBio Inc.), which gained approval for treatment of bladder cancer in 2024. Nogapendekin alfa inbakicept consists of an engineered IL-15 variant (N72D), which exhibits superagonist activity levels, combined with the IL-15Rα sushi domain fused to a human immunoglobulin (IgG) 1 Fc domain (Fig. 25B) (Han *et al.* 2011; Chamie *et al.* 2022). Growth factor engineering efforts include mutation of ligands and design of receptor decoys (Fig. 25C). Engineered growth factors have been most prominently applied in cancer and regenerative medicine. One success story for growth factor engineering is aflibercept (EYLEA®, Regeneron Pharmaceuticals). Aflibercept is an FDA-approved vascular endothelial growth factor (VEGF) receptor decoy protein consisting of two VEGF receptor domains fused to a human IgG1 Fc domain (Fig. 25D) (Holash *et al.* 2002; Papadopoulos *et al.* 2012). Aflibercept is clinically approved to treat neovascular eye diseases, and in the form of Ziv-Aflibercept (Zaltrap®), this drug is approved to treat metastatic colorectal cancer (Patel and Sun 2014). In addition to development of engineered cytokines and growth factors as therapeutics, there have been intensive efforts to conjugate or fuse these proteins to other molecules to enhance their pharmacological properties. Examples include: (i) localizing activities through fusion to a disease-targeting antibody; (ii) engineering conditional activation through the addition of a cleavable masking moiety; and (iii) half-life extension through conjugation to polyethylene glycol (PEG) or antibody Fc domains (Silver *et al.* 2021; An *et al.* 2024a).

Looking ahead, many exciting new engineering directions are emerging, which will transform our understanding of cytokine and growth factor biology, as well as our ability to fundamentally alter these natural molecules in order to develop more effective drugs. Particular areas of interest include: (i) development of synergistic combination therapies; (ii) fusion of native or engineered cytokines and growth proteins to other proteins such as antibodies, enzymes, and receptors; and (iii) devising novel strategies for targeting and conditional activation (Saxton *et al*. 2023; An *et al.* 2024a). Furthermore, the integration of experimental and computational evolutionary toolkits will empower higher throughput and more effective discovery workflows to overcome bottlenecks with targeting, manufacturability, and delivery to further advance the field (Wu *et al.* 2019; Li *et al.* 2025b). In particular, the emergence of *de novo* design for generation of cytokine mimetics with increased specificity and stability or even novel functionalities promises to widen the repertoire of therapeutic proteins (Huang *et al*. 2016c; Silva *et al.* 2019; Ren *et al.* 2022; Yang *et al.* 2023; Huang *et al.* 2024). Furthermore, exciting advances in gene delivery allow for synthesis of cytokine and growth factor engineering with innovative gene and cell therapy approaches. In particular, mRNA-based gene therapy offers a promising solution for some of the challenges of cytokine and growth factor therapies by enabling sustained delivery of these proteins, which typically have a short half-life when delivered as recombinant molecules (Zhen *et al*. 2025). In the case of cell engineering, chimeric antigen receptor (CAR) T cells have been modified to secrete cytokines, thus improving the immunostimulatory functions and persistence of CAR T cells for immunotherapeutic applications (Kizerwetter *et al.* 2023). Overall, the outlook is bright for cytokine and growth factor engineering endeavors to transform the scientific and medical landscapes in the years to come.

### Engineered proteins for vaccine development (Isabelle F. T. Viana and Roberto D. Lins^*^)

In addition to the development of antibodies and biopharmaceuticals, protein engineering has also reshaped modern vaccinology, enabling the design of immunogens with far greater stability, precision, and immunogenicity than traditional approaches. Advances in antigen engineering now allow for fine control over protein conformation, quaternary structure, and surface chemistry to optimize both biochemical behavior and immunologic performance. Engineered antigens can be tailored either to mimic the architecture of native key pathogens’ epitopes or to selectively expose target epitopes that drive potent neutralizing antibody responses.

Early vaccines relied on whole pathogens, either attenuated or inactivated, following classical empirical approaches established in the late nineteenth and early twentieth centuries (Vallery-Radot 1935; Salk 1955; Sabin *et al.* 1960). The advent of recombinant DNA technology marked a major shift, allowing individual antigens to be cloned and expressed in heterologous systems. A major milestone was passed in the 1970–80s with the development of a recombinant hepatitis B surface antigen produced in yeast. This protein self-assembled into subviral particles that mimicked those secreted by human cells during natural infection (Valenzuela *et al.* 1982), demonstrating for the first time that a single engineered protein could safely replace pathogen-derived material while successfully eliciting a protective immune response. This achievement established the conceptual foundation for modern recombinant subunit vaccines and catalyzed a wave of recombinant protein-based vaccines, particularly for pathogens that exhibit limited antigenic variability and for which immunity is protective. Nonetheless, highly challenging pathogens, such as HIV-1 and hepatitis C virus, have revealed the limitations of classical subunit strategies. Extensive antigenic diversity, low density of the envelope glycoprotein on the virion surface, conformational plasticity, glycan shielding, strain-specific amino acid variations, and transient exposure of relevant epitopes (vulnerability sites) have caused many vaccine attempts to fail in conferring relevant immunogenicity and inducing broadly neutralizing antibodies capable of preventing infection. These challenges prompted intensive efforts to understand the biology of the viruses and the determinants governing immune recognition. Beginning in the 1960s (Kendrew *et al.* 1960; Matricardi *et al*. 1972) and accelerating in the following decades, advances in X-ray crystallography, cryo-EM, and immunochemistry have revealed atomic-level details about the three-dimensional organization and chemical structure of antigens. Those details facilitated understanding how subtle conformational features shape immune recognition and laid the conceptual foundation for rational manipulation of antigen structure to improve vaccine responses. Importantly, this transition represented more than a technical improvement in structural resolution; it marked a shift from empirically selecting antigens toward deliberately engineering immune recognition based on molecular structure.

**Figure 26:**
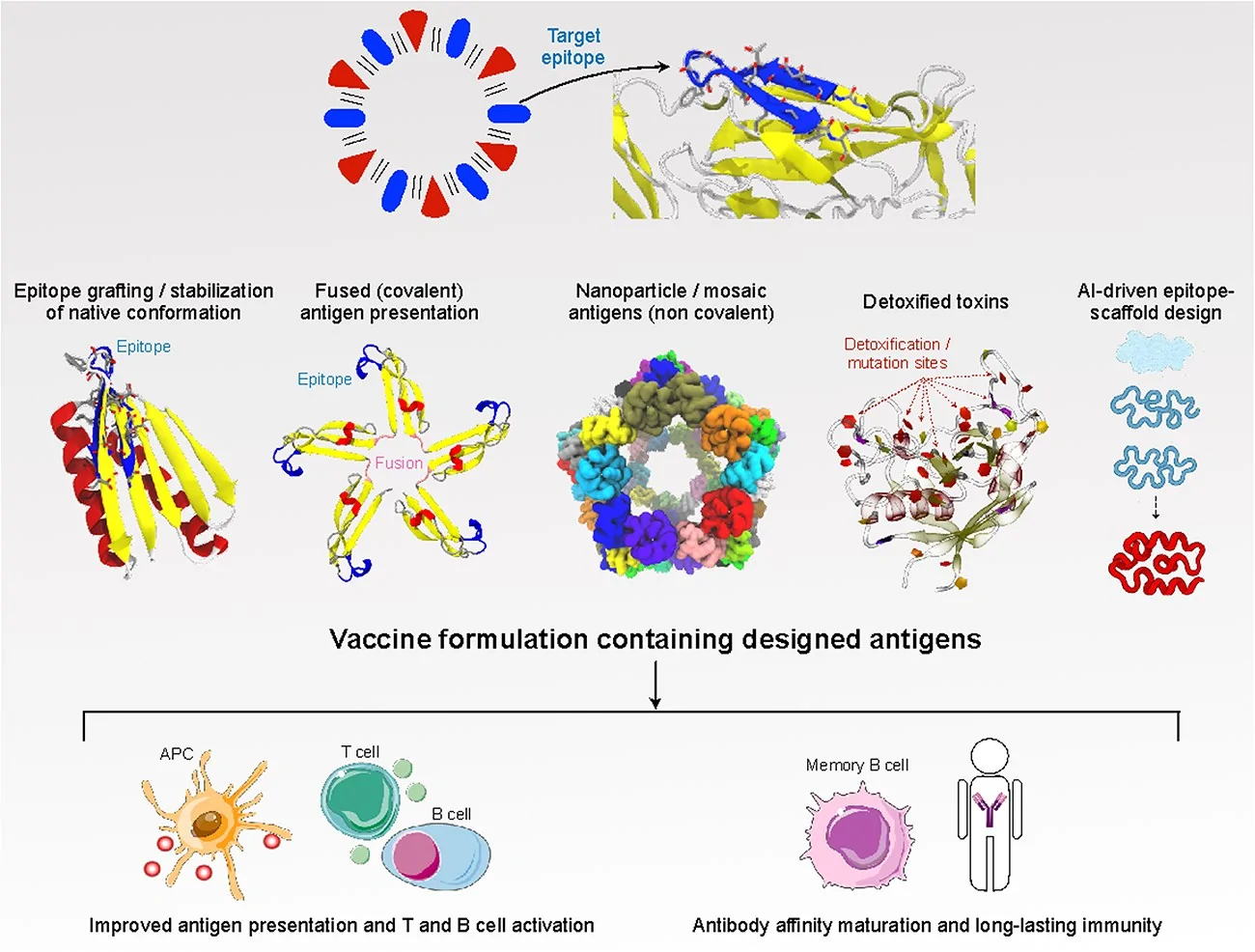
Antigen design strategies for protein engineered-based vaccines. Epitope grafting consists of inserting a previously identified epitope into a protein scaffold, subsequently modified to maintain its topology and vehicle the epitope as in the native/relevant conformation. Fused antigens consist of covalently linked subunits to enhance epitope density. Nanoparticle antigens are composed of several antigen units capable of assembling into a particle. The process of “detoxifying” toxins involves replacing key residues towards reducing toxicity while maintaining the epitopes of interest. Antigens can now be designed in the absence of templates by AI-driven generative models from either structure-based or language model instructions.

To elicit a robust protective response, vaccine antigens must prime the immune system with the specific components that are targets of neutralizing immunity. While T cell mediated immunity relies on the presentation of short unstructured peptides, the production of neutralizing antibodies by B cells requires the recognition of three-dimensional structured amino acid landscapes. Therefore, vaccine antigens must engage the appropriate naïve low-affinity B-cell receptors to induce clonal expansion and production of antibodies with the desired specificity in a way that facilitates the development of high-affinity antibodies. Early attempts at rational antigen engineering initially focused on improving stability and presentation, eventually evolving into sophisticated structure-guided and computationally driven strategies (Ofek *et al.* 2010a; McLellan *et al.* 2011; Azoitei *et al.* 2012). Purposeful manipulation of proteins has led to the development of a variety of approaches to meet the requirements for B-cell activation and antibody affinity maturation, including: (i) stabilization of native antigenic conformations (Viana *et al.* 2013; Crank *et al.* 2019; Tan *et al.* 2023); (ii) domains multimerization to enhance epitope density (Jagu *et al.* 2013; Jumadila *et al.* 2025); (iii) computational design of epitope scaffolds (Correia *et al.* 2010; Ofek *et al.* 2010b); (iv) engineering of mosaic antigens to broaden immune recognition (Barouch *et al.* 2010; Han *et al.* 2024); and (v) detoxification of bacterial toxins while preserving relevant epitopes (Rappuoli *et al.* 1995; Esmaeilnejad-Ahranjani *et al*. 2025) (Fig. 26). These efforts culminated in landmark achievements, such as the prefusion-stabilized RSV F protein, which demonstrated that rational design could overcome long-standing challenges in vaccine development (Huang *et al.* 2025). A transformative leap followed in the 2000s with the rise of computational protein design, more recently accelerated by several AI-assisted design methods. Platforms such as Rosetta (Leaver-Fay *et al.* 2011) and generative diffusion models like RFdiffusion (Watson *et al.* 2023) made it possible to model protein energetics, predict stabilizing mutations, and create synthetic scaffolds capable of presenting neutralizing epitopes with extraordinary precision. In this context, AI has primarily accelerated and expanded design capabilities that emerged from earlier structure-based approaches, although generative modeling may represent a more fundamental transition by enabling the *de novo* creation of proteins unconstrained by naturally occurring templates. Nevertheless, contemporary AI-assisted strategies still operate largely in synergy with classical protein engineering methodologies, including directed evolution, display technologies and structure-guided optimization, which remain essential for experimental validation, functional refinement, and selection of biologically relevant phenotypes. As a result, computationally designed antigens have been developed as subunits, nucleic acid encoding proteins, or protein nanoparticles. These engineered vaccines can now be found undergoing *in vitro* characterization (Rehmani *et al.* 2025), animal model validation (Weidenbacher *et al.* 2023; Miranda *et al.* 2024; Teixeira *et al.* 2024), and human clinical trials (Walls *et al.* 2020, 2021; Boyoglu-Barnum *et al.* 2021; Song *et al.* 2022). Nanoparticle platforms, such as ferritin and mosaic assemblies, have advanced to clinical trials, illustrating the power of programmable antigen geometry (Song *et al.* 2021; Cohen *et al.* 2024).

Protein engineering-based vaccines have matured into a data-driven, mainstream discipline. High-resolution structural biology, particularly cryo-EM, now routinely guides antigen design, while AI-driven generative models enable the creation of entirely new immunogens. Despite these rapid advances in AI-assisted design, automation, and high-throughput screening, achieving highly complex functional phenotypes remains considerably more challenging. It depends on the quality of the underlying experimental data and in the intrinsic complexity of populational immunological responses. Accurate structure determination, carefully curated sequence datasets, standardized immunological assays, and well-annotated functional measurements remain essential for training predictive models and validating engineered antigens. From the biological perspective, the ability to stabilize an antigen does not necessarily translate into the capacity to reproducibly elicit broadly neutralizing, durable, or precisely targeted immune responses. That depends on intricate, and only partially understood interactions between antigen structure, epitope accessibility, immune repertoire engagement, and affinity maturation pathways. This tension between structural stability and optimal biological function has emerged as a recurring theme across protein engineering disciplines and remains one of the major conceptual bottlenecks in next-generation vaccine design. Additional challenges include how to induce broad protective immunity against highly variable pathogens, predict T-cell responses, combine engineer antigens with optimal adjuvants and delivery systems, integrate functional immune complexity into computational design pipelines and translate engineered antigens into scalable, accessible vaccine platforms.

Notwithstanding these challenges, the trajectory of the field is unmistakable, as engineered proteins, whether used as direct antigens or encoded *via* nucleic acid sequences, are poised to stand at the forefront of next-generation vaccine development, powered by AI-driven synthetic biology pipelines, high-throughput screening, and rational design frameworks. Recent improvements in protein expression systems and thermostability support global manufacturability and distribution. Therefore, these advancements position protein engineering as a cornerstone technology to provide most effective responses against current and emerging infectious threats.

## Detection, imaging, and diagnostics

### Fluorescent proteins (Sohum Mehta and Jin Zhang^*^)

**Figure 27:**
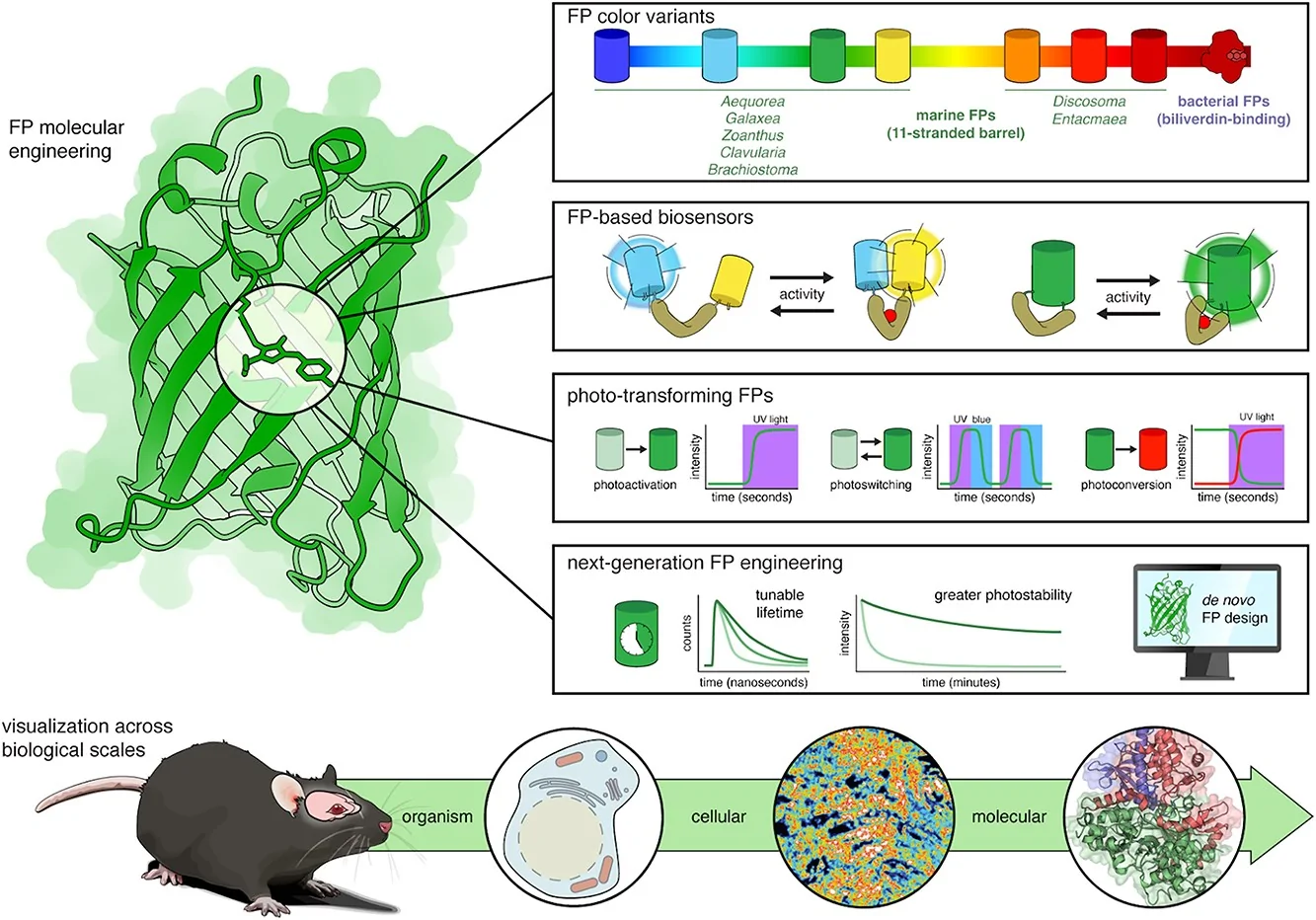
Fluorescent protein technology for visualizing biological processes. *Left*: The discovery of green fluorescent protein inspired decades of molecular engineering that continues to yield innovative fluorescent protein (FP)-based tools. *Right*: A rainbow of FP color variants (top) derived from various marine species spans the visible spectrum from blue to far-red, along with near-infrared FPs derived from biliverdin-binding bacterial proteins. A myriad of FP-based biosensors (upper middle), utilizing either FRET- (left) or single-FP- (right)-based designs, enable robust, dynamic visualization of biochemical activities. Photo-transforming FPs (lower middle), which undergo irreversible photoactivation (left), reversible photoswitching (middle), or photoconversion between different colors (right; e.g. green-to-red) upon illumination at specific wavelengths, enable powerful and sophisticated imaging strategies. Meanwhile, next-generation FP engineering (bottom) efforts to achieve tunable fluorescence lifetimes, greater photostability, and even *de novo* design of entirely new FPs, continue to push the envelope. *Bottom*: These technologies allow researchers to visualize biological processes across scales, from the whole organism down to the molecular level.

“A protein giving solutions that look slightly greenish in sunlight though only yellowish under tungsten lights, and exhibiting a very bright, greenish fluorescence in the ultraviolet of a Mineralite, has also been isolated from [Aequorea] squeezates” (Shimomura *et al*. 1962). With this throwaway observation—a footnote on the purification of Aequorin—Osamu Shimomura unwittingly sparked a revolution. Thirty years later, working from clones generated by Douglas Prasher (Prasher *et al.* 1992), Martin Chalfie would demonstrate *Aequorea victoria* green fluorescent protein (GFP) as an intrinsically fluorescent, genetically encoded probe in living cells and animals (Chalfie *et al.* 1994), and Roger Tsien would engineer GFP variants with improved performance and capability for live-cell imaging (Heim *et al*. 1994; Heim *et al*. 1995; Heim and Tsien 1996) (Fig. 27). What started as a decades-old curiosity quickly spawned an indispensable toolkit for biological research, earning Shimomura, Chalfie, and Tsien the 2008 Nobel Prize in Chemistry.

Roger Tsien’s visionary use of molecular engineering to open new frontiers in visualizing life processes remains the guiding light of fluorescent protein (FP) technology. Early blue, cyan, and yellow GFP variants (Heim *et al*. 1994; Heim and Tsien 1996) soon yielded Förster resonance energy transfer (FRET)-based biosensors (Miyawaki *et al.* 1997) for monitoring a host of molecular events inside cells (Gest *et al.* 2024). Coral-derived and non-standard, bacterial FPs subsequently expanded the spectral pallet (Rodriguez *et al.* 2017) to red and even near-infrared wavelengths, enabling highly multiplexed labeling and deeper tissue penetration for *in vivo* imaging. Successful insertion of receiver domains into circularly permuted GFP (Baird *et al*. 1999) gave rise to a class of single-fluorophore biosensors capable of achieving *in vivo* biosensing (Gest *et al.* 2024). Intrinsic FP photophysics have also been leveraged to engineer variants that undergo irreversible or reversible on/off switching or that change color, allowing more precise tracking of biomolecules, enhanced multiplexing and quantification, and functional super-resolution imaging of genetically encoded biosensors (Gest *et al.* 2024). FPs have steadily matured from passive markers of gene and protein expression to smart labels for visualizing dynamic biological processes across scales.

Now entering their fourth decade, what lies ahead for FPs? Engineering focused on fluorescence lifetime has yielded improved FPs (Goedhart *et al.* 2012; Bindels *et al.* 2017) for more robust quantification of molecular events in cells, as well as temporal-spectral multiplexing with new “time-resolved” FPs (Tan *et al.* 2025). Recent efforts have also delivered FPs with dramatically improved photostability (Hirano *et al.* 2022; Ando *et al.* 2024) to withstand prolonged illumination during long-term or super-resolution imaging, as well as more chemically stable FPs (Katayama *et al.* 2020; Campbell *et al.* 2022; Zhang *et al.* 2024a) compatible with harsh intracellular environments or stringent chemical processing. Structural and molecular insights, long crucial for FP engineering, are also beginning to emerge regarding the molecular determinants of a broader range of photophysical properties, including quantum yield (Prangsma *et al.* 2020) and photostability (Ahmed *et al.* 2025). Meanwhile, *de novo* engineering efforts are teasing the development of entirely new types of FPs (Liu *et al.* 2026). We therefore look forward to a very bright future for FP technology.

### Luciferases (Rachna Sharma and Hui-wang Ai^*^)

**Figure 28:**
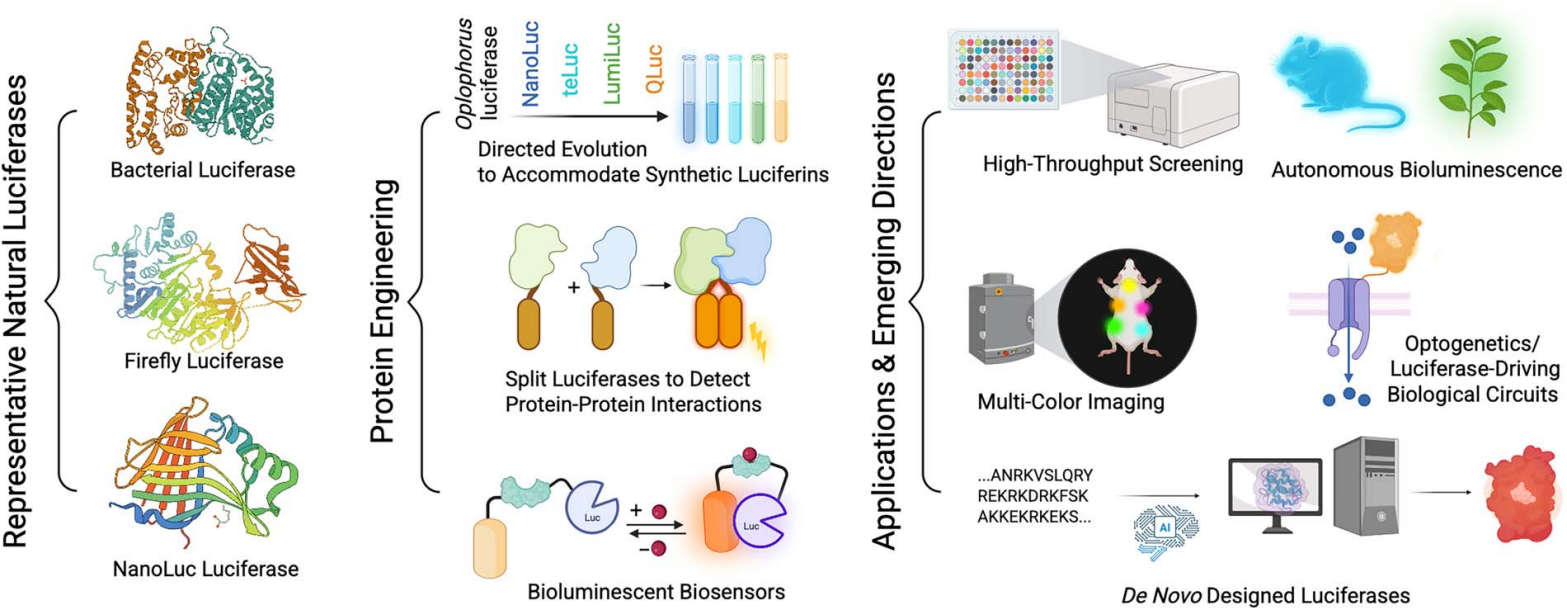
Overview of luciferase engineering and bioluminescence technologies. *Left:* Representative structures of several natural luciferases, which serve as the foundation for developing engineered variants. Structures were rendered using Protein Data Bank (PDB) entries 1BRL (Fisher *et al.* 1995), 1LCI (Conti *et al*. 1996), and 5IBO (Lovell *et al.* 2016). *Center:* Representative luciferase engineering research directions to enhance enzyme performance and expand functionality. *Right:* Applications and emerging directions illustrating the broad impact of engineered luciferases. Created in BioRender. Ai (2025) https://BioRender.com/zovlmop.

The phenomenon of bioluminescence has fascinated humans for millennia, with documented observations dating back to ancient civilizations. However, it was not until the cloning of several luciferase genes, beginning in the 1980s, that luciferases emerged as powerful tools in biological and biomedical research (Fig. 28) (Yeh and Ai 2019). These enzymes catalyze the oxidation of small-molecule substrates called luciferins in the presence of oxygen, emitting visible light without the need for external illumination (Yeh and Ai 2019). This intrinsic property enables imaging with a high signal-to-noise ratio and minimal background or phototoxicity, offering distinct advantages over fluorescent labels. Over the past two decades, advances in biomolecular engineering including rational mutagenesis, directed evolution, synthetic luciferin development, and fusion protein design have further enhanced brightness, spectral diversity, and functional versatility. As a result, the luciferase toolbox now supports diverse applications ranging from *in vitro* assays and drug screening to intracellular biosensing and whole-animal imaging.

A widely used luciferase is NanoLuc, a small (19 kDa) enzyme derived from the deep-sea shrimp *Oplophorus gracilirostris*, which utilizes a synthetic furimazine substrate and is ~100-fold brighter than conventional firefly luciferase (Hall *et al.* 2012). Split NanoLuc systems, such as NanoBiT (LgBiT/SmBiT), have been developed for detecting protein–protein interactions (Dixon *et al.* 2016). To extend the utility of blue-photon-emitting NanoLuc for *in vivo* imaging, the enzyme has been engineered to accommodate luciferin analogs with extended π-conjugation, shifting the emission toward the red spectrum for enhanced tissue penetration (Yeh *et al.* 2017; Xiong *et al.* 2022). In addition, NanoLuc variants have been fused to long-wavelength emitting FPs to achieve red-shifted emission through bioluminescence resonance energy transfer (BRET) (Chu *et al.* 2016; Yeh *et al.* 2017; Tian *et al.* 2022a). Another prominent example of luciferase engineering for deep-tissue imaging is AkaLuc, a firefly luciferase variant optimized together with the synthetic substrate AkaLumine to enhance brightness and red-shifted near-infrared emission (~677 nm), thereby enabling highly sensitive *in vivo* imaging (Iwano *et al.* 2018). Furthermore, luciferases are increasingly used to construct bioluminescent biosensors that detect metal ions, signaling molecules, enzyme activities, and various other targets (Suzuki *et al.* 2016; Arts *et al.* 2017; Oh *et al.* 2019; Su *et al*. 2019; Tian *et al.* 2022a; Halbers *et al.* 2024; Zhao *et al.* 2024). These innovations have greatly expanded the utility of luciferases as dynamic, non-invasive reporters of biological processes.

Looking ahead, luciferase engineering is moving into new frontiers. The integration of computational protein design with directed evolution is enabling the generation of *de novo* luciferases with customized structures and tailored substrate specificity (Yeh *et al.* 2023). Efforts are also being invested to derive orthogonal luciferase-luciferin pairs to support multiplexed bioluminescence imaging across distinct spectral channels (Jones *et al.* 2017; Chen *et al.* 2025c). Meanwhile, luciferases are being integrated into optogenetic systems, serving as light sources to drive engineered biological circuits (Berglund *et al.* 2016; Adir *et al.* 2022). With all genetic components now known, bacterial and fungal luciferases are being reengineered into fully autonomous bioluminescent systems that require no exogenous substrates (Close *et al.* 2010; Kotlobay *et al.* 2018; Kusuma *et al.* 2024; Shakhova *et al.* 2024; Kiszka *et al.* 2025). These advances are pushing the field toward next-generation, multi-functional luciferase platforms for truly non-invasive imaging, diagnostics, and synthetic biology applications.

### Gas vesicle proteins (Zhiyang Jin and Mikhail G. Shapiro^*^)

**Figure 29:**
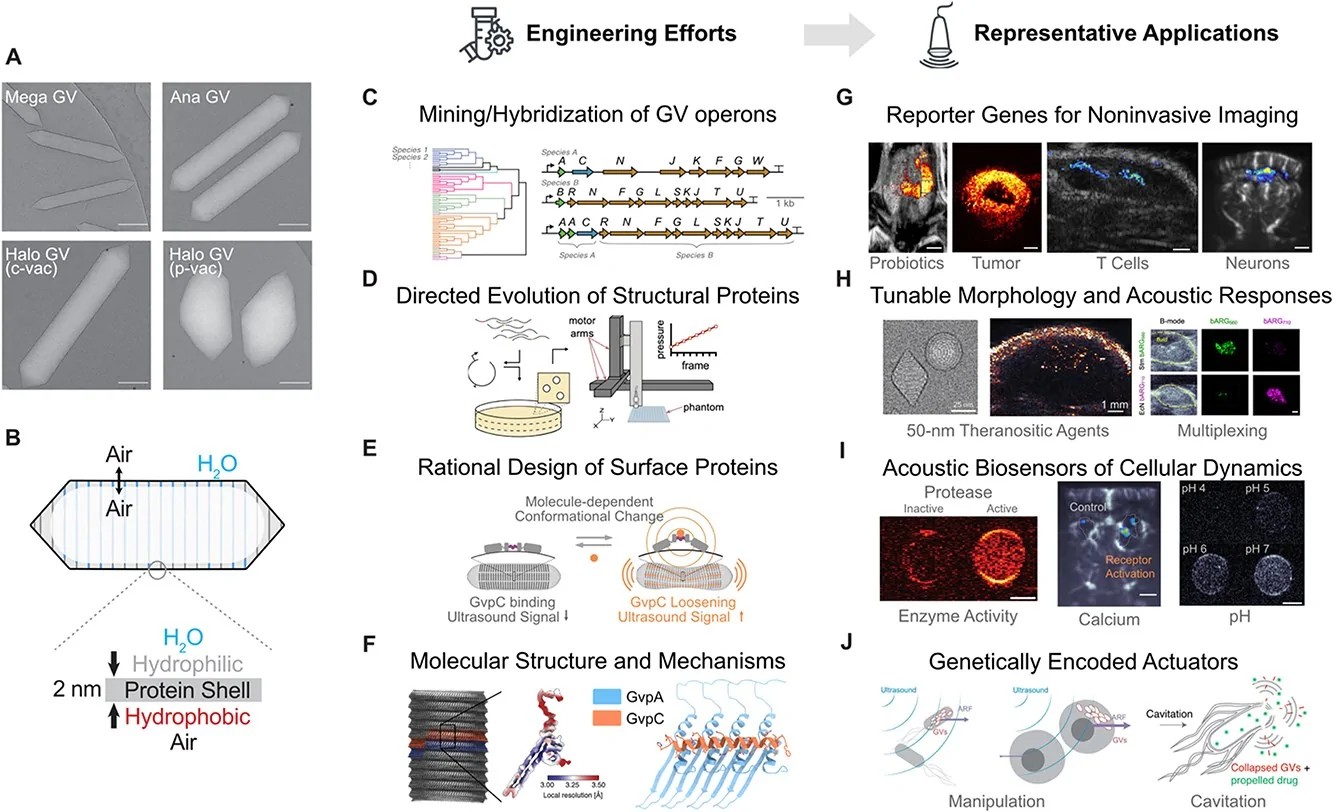
Engineering of gas vesicle (GV) proteins and representative applications. (A) Representative cryo-EM of GVs from different organisms. Adapted with permission from Dutka *et al.* (2021). (B) Schematics of a GV showing its ability to allow gas diffusion across the shell and exclude water condensation inside with the physiochemical properties of its shell. (C) Schematics showing the genomic mining and hybridization of GV gene clusters from different organisms. Adapted with permission from Hurt *et al.* (2023). (D) Schematics showing the directed evolution and ultrasound screening pipeline for GVs. Adapted with permission from Nyström *et al.* (2025). (E) Schematics showing the GvpC-mediated ultrasound signal changes. (F) Schematics showing the molecular structures of the GV shell and its primary structure proteins. Adapted with permission from Dutka *et al.* (2023) and Huber *et al.* (2023). (G) Representative ultrasound images of different types of cells expressing GVs as reporter genes *in vivo*. Adapted with permission from Hurt *et al.* (2023), Shivaei *et al.* (2024, 2025), and Buss *et al.* (2025). (H) Left: Representative electron micrograph of 50-nm GVs. Middle: Representative ultrasound images of accumulation of systematically administered 50-nm GVs in tumor. Right: Representative ultrasound images of therapeutic bacteria expressing GVs with different acoustic properties for multiplexed imaging. Adapted with permission from Ling *et al.* (2024) and Nyström *et al.* (2025). (I) Representative ultrasound images of acoustic biosensors sensing enzyme activity (left), calcium dynamics (middle) and pH (right). Adapted with permission from Lakshmanan *et al.* (2020), Jin *et al.* (2023), and Terwiel *et al.* (2025). (J) Schematics of GVs as genetically encoded mechanical actuators for manipulation (left) and cavitation-enhanced therapy (right). Adapted with permission from Bar-Zion *et al.* (2021) and Wu *et al.* (2023). Scale bars = 100 nm for (A), 25 nm (H, leftmost panel), and 1 mm for (G, I, and the right two panels of H).

In 1895, German microbiologists noticed that certain aquatic bacteria float by forming intracellular compartments filled with air. Over the next century, a handful of curious biochemists studied how these air-filled bodies are created by an unusual class of proteins called gas vesicles (GVs) (Walsby 1994). Then, in 2014, these little-known proteins suddenly found a new life as a “GFP for ultrasound” (Shapiro *et al.* 2014), enabling biomolecular and cellular imaging in deep tissues. GVs are air-filled self-assembling protein nanostructures with dimensions of ~100 nm that evolved to help photosynthetic microbes float (Walsby 1994) (Fig. 29A). GVs’ 2 nm-thick protein shell excludes water but allows gases from surrounding media to partition inside (Walsby 1994; Dutka *et al.* 2023; Huber *et al.* 2023) (Fig. 29B). We discovered that because GVs are less dense and more compressible than cells and tissues, they produce strong ultrasound contrast (Shapiro *et al.* 2014). A decade after this foundational finding, GVs are being applied as injectable contrast agents (Shapiro *et al.* 2014; Ling *et al.* 2024; Shen *et al.* 2024), genetically encoded reporter genes (Bourdeau *et al.* 2018; Farhadi *et al.* 2019; Hurt *et al.* 2023; Shivaei *et al.* 2024, 2025; Buss *et al.* 2025), dynamic molecular biosensors (Lakshmanan *et al.* 2020; Jin *et al.* 2023; Terwiel *et al.* 2025), mechanical actuators (Wu *et al.* 2023), and explosive cavitation agents for ultrasound (Bar-Zion *et al.* 2021), as well as a few applications in optics and magnetic resonance (Fig. 29C–J).

As their assembly involves more than 8 genes (Hurt *et al.* 2023), GVs are more challenging to express and engineer than most proteins. Nevertheless, at the whole-operon scale, genomic mining of GV gene clusters and construction of compact multi-gene expression cassettes yielded GV constructs with high expression and ultrasound contrast in a variety of cell types (Hurt *et al.* 2023) (Fig. 29C), making it possible to non-invasively track gene expression *in vivo* in probiotics (Hurt *et al.* 2023; Buss *et al.* 2025), tumors (Hurt *et al.* 2023), T-cells (Shivaei *et al.* 2024), and neurons (Shivaei *et al.* 2025) (Fig. 29G). Meanwhile, directed evolution of GVs’ key structural proteins and assembly factors (Hurt *et al.* 2024; Nyström *et al.* 2025), along with hybridization of those from different gene clusters (Shen *et al.* 2024) (Fig. 29C and D), generated constructs with astonishing morphology (e.g. 50-nm GVs as injectable theranostic agents) (Ling *et al.* 2024; Shen *et al.* 2024) or tunable acoustic properties (e.g. pressure responses for multiplexing) (Hurt *et al.* 2024; Nyström *et al.* 2025) (Fig. 29H). Moreover, GvpC, a surface protein that mechanically stiffens GV shell, was rationally designed to modulate GVs’ ultrasound contrast in response to different biomolecules (e.g. enzymes (Lakshmanan *et al.* 2020), calcium ions (Jin *et al.* 2023), or pH (Terwiel *et al.* 2025)) (Fig. 29E), resulting in the first acoustic biosensors enabling ultrasound readout of dynamic cellular activity (Fig. 29I). With most of those engineering efforts validated in proof-of-concept *in vivo* applications, GVs now represent a class of powerful tools for non-invasive imaging of cellular activity and a versatile engineering platform for more enabling technologies to come.

Admittedly, many challenges await. For instance, minimization of the large gene cluster and reducing potential side effects of GV expression (e.g. cellular burden and immunogenicity) are needed for broader applications of GVs. Both may involve engineering many or all the protein components in the cluster, which is impeded by incomplete knowledge on the function of each chaperone and the assembly mechanisms. Fortunately, there are efforts attempting to elucidate the protein–protein interaction and the biochemical mechanisms in GV assembly (Jost and Pfeifer 2022; Iburg *et al.* 2024). Along with the recently published cryo-EM structures of GVs (Dutka *et al.* 2023; Huber *et al.* 2023) (Fig. 29F) and fast-evolving computational tools for protein designs, the pieces are coming together for more rational, structure-guided designs to improve GV performance and create new biosensors. In parallel to protein engineering efforts, genetic circuits and delivery methods are constantly being improved as a joint solution. We are excited to unfold a future where GVs and their derivatives make non-invasive biomolecular imaging as easy and impactful as fluorescent imaging.

### *In vitro* diagnostics (Chenggang Xi and Andy Hsien-Wei Yeh^*^)

**Figure 30:**
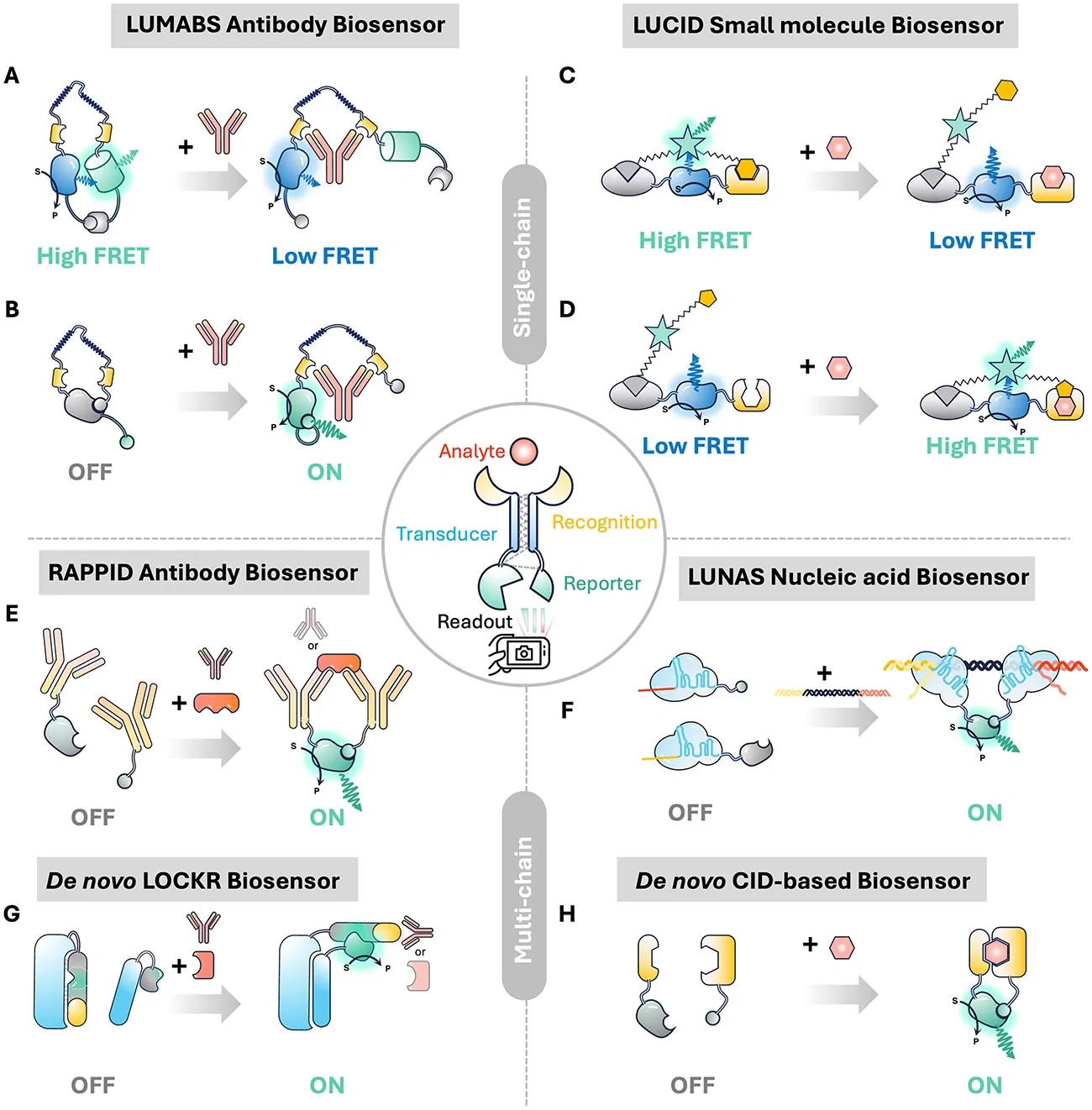
Modular design of protein-based biosensors. (A and B) schematic of single-chain LUMABS biosensors for antibody detection. These constructs incorporate two epitope domains as the recognition module and utilize either (A) FRET pairs or (B) split luciferase reporters to monitor conformational switching. Upon antibody binding, the closed conformation opens, disrupting energy transfer, or luciferase complementation, which results in color shifts or luminescence activation. (C and D) Schematic of semisynthetic LUCID biosensors for small-molecule detection. A ligand receptor and a luciferase reporter domain are covalently linked *via* a fluorophore-tethered ligand analogue. Target binding modulates fluorophore proximity by either (C) displacing or (D) recruiting the tethered ligand, leading to ratiometric color changes in the luminescent signal. (E) Schematic of multi-chain RAPPID biosensors for antibody or antigen detection. Commercial antibodies are photo-conjugated to split luciferase fragments, which reconstitute enzymatic activity upon binding to either antigens or anti-drug antibodies. (F) Schematic of the LUNAS platform for nucleic acid detection. Split luciferase fragments are fused to dCas9, which is guided by programmable gRNAs. When two gRNA-dCas9 complexes bind adjacent sites on the target DNA, they colocalize the luciferase fragments to enable target-specific luminescence. (G) Schematic of LOCKR-based biosensors for protein biomarker detection. A recognition domain is fused to the latch domain, stabilized in a closed state by a *de novo*-designed Cage protein. Target binding provides the thermodynamic driving force to allow the Key-Cage association and facilitate split luciferase reconstitution. (H) Schematic of a *de novo*-designed chemically inducible dimerization (CID) biosensor for small-molecule detection. Ligand binding (e.g. cortisol) induces heterodimerization of the designer dimer fused to split *de novo* luciferase fragments, triggering luminescence activation.

Protein-based biosensors are emerging *in vitro* diagnostic (IVD) tools that couple analyte-induced conformational changes in a recognition domain to a reporter module, converting specific target binding events into amplified, measurable signals (Fig. 30). Compared with conventional analytical methods (e.g. ELISA), protein-based biosensors enable *in situ*, wash-free, mix-and-read, and quantitative detection of target analytes with minimal sample preparation and rapid turnaround. For the readout, luciferase-catalyzed luminescence can achieve orders of magnitude higher sensitivity than fluorescence in opaque biological matrices (e.g. blood or serum), where autofluorescence background or hemoglobin absorbance dominates. Besides, excitation-free luminescence can be captured with simple devices (e.g. a camera or a smartphone), which is advantageous for point-of-care (POC) diagnostics.

Recent advances in protein engineering have enabled modular assembly of biosensors capable of detecting clinically relevant targets such as antibodies, metabolites, drugs, antigens, nucleic acids, and other protein biomarkers. Here, we highlight representative design principles toward the development of efficient luminescent biosensors by considering the following key factors: (i) recognition domains that undergo sufficient target-induced conformational change or thermodynamic driving force at affinities matched to physiologic or pathologic analyte levels; (ii) scaffolds that efficiently transduce structural changes into quantifiable luminescent outputs; and (iii) programmable reporter modules, commonly implemented as split enzymes or FRET pairs (see Section Fluorescent proteins (Sohum Mehta and Jin Zhang^*^)).

The Merkx lab developed the LUMABS (bioluminescent antibody sensor) platform to detecting therapeutic antibodies with picomolar limits of detection using single-chain switches that combine epitope recognition sites and provide either ratiometric FRET changes (Arts *et al.* 2016) (Fig. 30A) or intensiometric signals *via* split luciferase (Gräwe and Merkx 2022) (Fig. 30B), which can be captured by a camera in solution or on paper (Tenda *et al.* 2018). For small-molecule detection, the Johnsson lab pioneered the semisynthetic LUCID (luciferase-based indicator of drugs) platform, in which a receptor-luciferase fusion is covalently labeled with a fluorophore-tethered ligand analog (Fig. 30C and D). Binding of metabolite (Yu *et al.* 2018) or drug (Griss *et al.* 2014) modulates fluorophore proximity through ligand displacement or recruitment, yielding ratiometric color shifts. The Merkx lab also reported RAPPID (ratiometric plug-and-play immunodiagnostics), where split-luciferase-labeled antibodies reconstitute enzymatic activity upon antigen binding (Ni *et al.* 2021) (Fig. 30E), and LUNAS (luminescent nucleic acid sensor) that uses dCas9-split luciferase fusions for programmable DNA detection (van der Veer *et al.* 2023) (Fig. 30F). Together, these approaches employed experimental approaches (e.g. linker optimization and domain swapping) to showcase the diverse development of luminescent sensors for the detection of proteins, small molecules, and nucleus acids in POC settings.

In recent years, *de novo* protein design has expanded biosensor functionality with tailored architectures and functions without relying on naturally occurring proteins. For instance, the Baker lab created LOCKR (latching orthogonal cage-key protein) switches that provide a modular platform to couple target binding with split luciferase reconstitution *via* a *de novo* designed Cage-Key system (Fig. 30G). By exchanging binding partners, LOCKR biosensors have been configured to detect BCL-2, IgG, HER2, Botulinum neurotoxin, cardiac troponin I, anti-hepatitis B antibody, and the SARS-CoV-2 spike protein (Quijano-Rubio *et al.* 2021). The reversibility of LOCKR biosensors has been leveraged to quantify neutralizing antibodies against various variants of concern of SARS-CoV-2 in a wash-free homogeneous format (Zhang *et al.* 2022). For ligand detection, the Yeh lab designed *de novo* cortisol-inducible dimerization modules that, when coupled to *de novo* split luciferase using a structure-prediction pipeline (Fig. 30H), yield a fully computationally designed cortisol biosensor with over 300-fold luminescent response, picomolar sensitivity, and rapid imaging by standard cameras (Chen *et al.* 2025b). Compared to natural proteins, *de novo* biosensors and luciferases offer exceptional stability, tunability, glow-type emission kinetics (Xi and Yeh 2026), and manufacturability—traits essential for reproducible and field-ready diagnostics. Looking forward, AI-powered protein design has started making biosensor structures increasingly predictable by computation, helping enrich and prioritize designs before experimental optimization. As computational protein design and AI-based generative models continue to advance, custom-made biosensors built from the first principles will drive the next wave of programmable, protein-based biosensors for IVD.

### Engineered proteins for spatial proteomics (Amy M. Weeks^*^)

**Figure 31:**
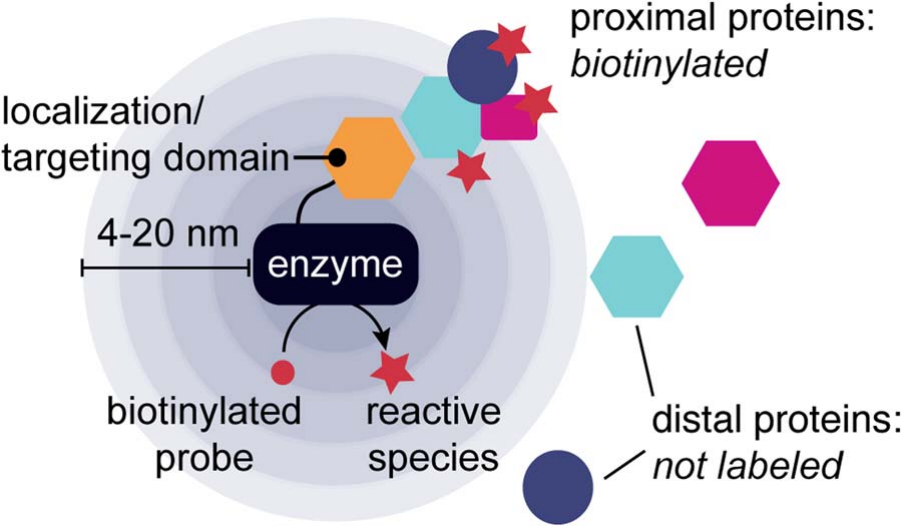
Engineered proximity-labeling enzymes for spatially resolved proteomics. An enzymatic catalyst is targeted to a specific subcellular location and generates a diffusible reactive species that biotinylates nearby proteins. Proximity-based biotinylation enables enrichment and analysis of subcellular subproteomes by tandem mass spectrometry.

Cells organize their biochemical processes by localizing proteins to macromolecular complexes and subcellular compartments. Historically, the interaction landscape of macromolecules in cells was assessed with techniques such as co-immunoprecipitation, which requires high-affinity interactions, and yeast two-hybrid assays, which are prone to false positives. Engineered enzymes that capture transient interactions and spatial proximity have transformed our understanding of subproteomes associated with specific compartments and complexes (Fig. 31). These tools are based on the principle that positioning a catalyst capable of generating diffusible reactive species near a protein of interest results in proximity-dependent labeling of nearby macromolecules (Choi-Rhee *et al*. 2004). Incorporating an affinity handle such as biotin into the reactive species enables labeled proteins to be isolated and analyzed by tandem mass spectrometry (Roux *et al.* 2012). The effective labeling radius of these proximity labeling proteins is determined by how far the reactive species can diffuse in the cellular environment before quenching, and the development of catalysts that generate distinct reactive species now allows spatial proteome mapping across scales ranging from organelles to protein complexes (Roux *et al.* 2012; Rhee *et al.* 2013; Geri *et al.* 2020; McCutcheon *et al.* 2020).

The field of proximity biotinylation began with the development of BirA-R118G (BioID), a single point mutant of the *E. coli* biotin ligase BirA that releases the reactive intermediate biotin-5′-AMP (Choi-Rhee *et al*. 2004). Since this initial report, a diverse suite of enzymes has been engineered for proximity labeling, including APEX (Rhee *et al.* 2013), which generates phenoxyl radicals from hydrogen peroxide and biotin-phenol, and miniSOG, an engineered flavin-binding protein that produces singlet oxygen upon blue-light irradiation (Zheng *et al.* 2023). Protein engineering efforts have further enhanced these systems, yielding variants such as APEX2 (Lam *et al.* 2015) with improved sensitivity and TurboID with faster labeling kinetics (Branon *et al.* 2018). These advances have expanded the utility of proximity labeling, enabling higher sensitivity enrichment of organellar membrane proteins, deployment across model organisms, and construction of a large-scale proximity biotinylation map of the human cell (Go *et al.* 2021).

Recent proximity labeling efforts have focused on tools that resolve proteome-scale biological organization with unprecedented granularity. Hybrid systems that integrate engineered target-binding proteins with small-molecule photocatalysts can generate short-lived reactive species such as carbenes and nitrenes, substantially tightening labeling radius (Geri *et al.* 2020; McCutcheon *et al.* 2020; Tay *et al.* 2023; Lin *et al.* 2024). New classes of engineered enzymes can directly interrogate proteoforms with PTMs, such as those that are glycosylated or proteolytically cleaved (Joeh *et al.* 2020; Weeks *et al.* 2021). These innovations are pushing proximity labeling beyond cataloging protein–protein interactions, toward technologies that can identify specific interaction surfaces and reveal how PTMs sculpt the spatial organization of the proteome.

## Synthetic biology and control systems

### Optogenetics (Chandra L. Tucker^*^)

**Figure 32:**
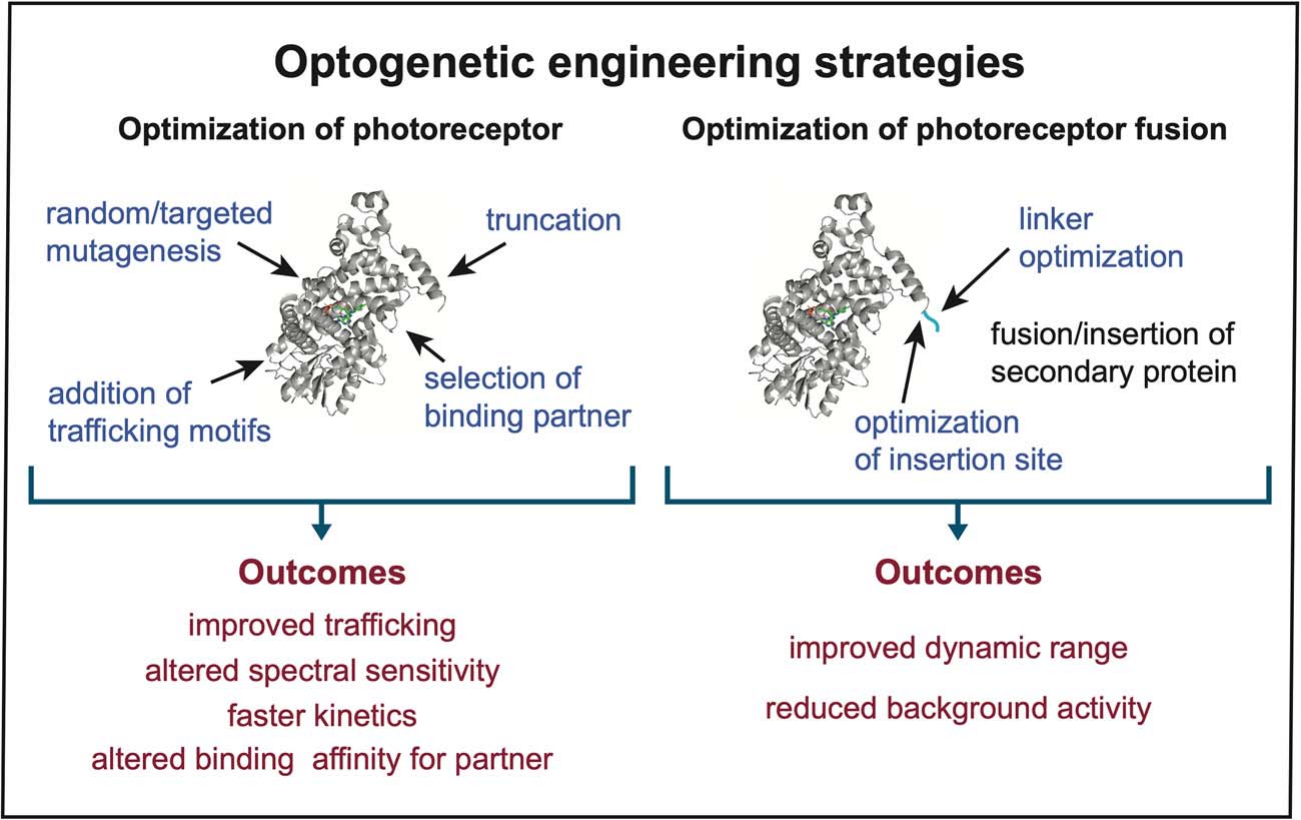
Engineering of photoreceptor proteins or photosensory domains. Photoreceptor and photosensory domains have been strategically engineered in a variety of ways to achieve optimal light-dependent function for control and manipulation of biological activities. The pictured photoreceptor is AtCRY2 (PDB ID 6K8I) (Ma *et al.* 2020).

Optogenetics, enabling the use of light for precise control of cell functions, has transformed neuroscience and cell biology. Advancements in this field have been brought about through the development of light-controlled protein reagents, derived from natural, or engineered photosensory proteins, using protein engineering strategies (Moglich and Moffat 2010) (Fig. 32). A variety of strategies, including directed evolution and mutagenesis screening, insertional libraries, linker randomization, and rational design, have been utilized to build and optimize these reagents.

Most optogenetic tools are derived from light-sensing proteins found in Nature. While some pioneering optogenetic tools were initially implemented with minimal engineering (the initial testing of channelrhodopsin-2 from *Chlamydomonas reinhardtii* in mammalian neurons (e.g. Boyden *et al.* 2005), further engineering has often been required to optimize function. Molecular engineering strategies include the addition of trafficking motifs (Gradinaru *et al.* 2010); mutagenesis to disrupt native interaction domains (such as localization signals, self-interaction motifs, or binding motifs) that interfere with tool use (Wu *et al.* 2009; Kennedy *et al.* 2010; Kawano *et al.* 2015); and construction of chimeric fusions or insertions of photosensory domains with effectors (Levskaya *et al.* 2009; Moglich *et al*. 2009; Wu *et al.* 2009; Lee *et al.* 2014; Dagliyan *et al.* 2016; Zhu *et al*. 2023a). Mutagenesis and chimeric domain swapping have also been used to alter spectral sensitivity (Lin *et al.* 2013; Oda *et al.* 2018), improve photoresponse kinetics (Lin *et al.* 2009; Gunaydin *et al.* 2010; Klapoetke *et al.* 2014), or modulate binding affinities (Guntas *et al.* 2015; Kawano *et al.* 2015).

One productive approach for optogenetic tool development combines mutagenesis with high-throughput selection or directed evolution strategies, such as phage display (see Section *In vitro* display (Takuya Terai and Naoto Nemoto^*^)) and binding assays, yeast surface display (see Section Cell-based display (Owen T. Porth and K. Dane Wittrup^*^)), or yeast two-hybrid (Guntas *et al.* 2015; Jang and Woolley 2021). Beyond improvement of the photosensory molecules themselves, molecular engineering has also been applied to attach target proteins. Light-dependent reconstitution of split proteins, a common approach, requires optimization of multiple factors including protein split sites, linkers, and binding affinities of split protein fragments (Kennedy *et al.* 2010; Kawano *et al.* 2015; Dagliyan *et al.* 2018; Lee *et al.* 2025).

Advances in molecular engineering methodologies, *in silico* protein design, and the affordability of DNA technologies have further extended the possibilities for optogenetic engineering. *De novo* protein design and structure prediction tools can be applied to rationally design new light-dependent binders or optimize existing tools. Modern protein modeling and design approaches, backed by traditional mutagenesis and screening, will likely usher in an exciting wave of optogenetic engineering.

### Genome and epigenome engineering (Izaiah J. Ornelas and James K. Nuñez^*^)

**Figure 33:**
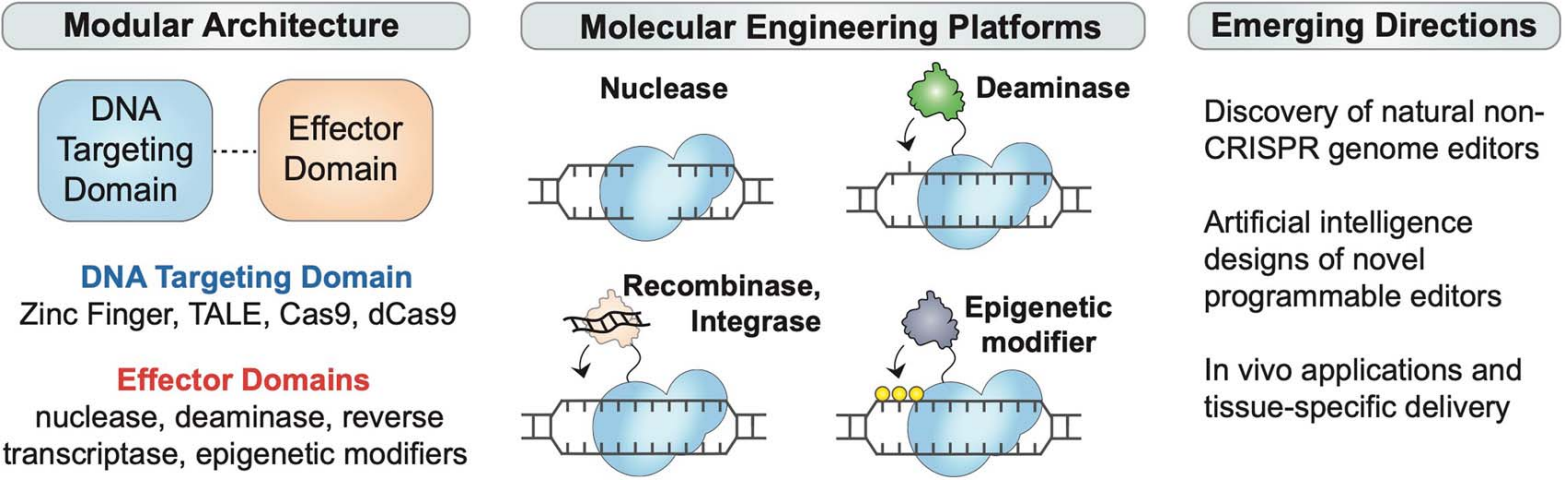
Design architecture of engineered proteins for genome editing. Genome editors consist of a DNA targeting domain and an effector for genome and epigenome editing. Examples of modern platforms include nucleases, base editors, integrases, and epigenetic editors. Ongoing and future work in the field include discovering new modalities for genome editing, the use of AI for optimizing and/or designing new editors, and tissue-specific delivery for *in vivo* therapeutic applications.

Targeted genome and epigenome engineering tools have transformed basic research and wide-ranging applications such as human therapeutics and agriculture. The core architectural design that underlies nearly all modern genome and epigenome editors is modular: (1) a DNA targeting domain directs localization to a specified genomic locus; and (2) an effector domain (e.g. nuclease, deaminase, reverse transcriptase, epigenetic modifier) that carries out genetic or epigenetic changes at the target locus (Gupta and Kumar 2025) (Fig. 33). Early programmable systems relied on effectors tethered to DNA-binding domains (DBDs) such as zinc finger proteins (Kim *et al*. 1996) and transcription activator–like effector proteins that can be engineered rationally to recognize specific DNA sequence (Miller *et al.* 2011). The advent of CRISPR-Cas9 from *Streptococcus pyogenes* in 2012 initiated a revolution in the field due to the facile programmability of Cas9, simply by altering the DNA-targeting sequence of a single guide RNA (sgRNA) (Jinek *et al.* 2012). Cas9 is an RNA-guided nuclease that can be further engineered into a nuclease-dead protein (dCas9) that retains its DNA targeting property that can be fused to genome- or epigenome-modifying effectors (Qi *et al.* 2013).

CRISPR editors can be associated with off-target editing, low editing efficiency, and delivery constraints. To address these, engineering efforts have applied rational design (e.g. PAM-relaxed variants, optimal sgRNA selection, effector selection) and directed evolution (e.g. mutagenesis/selection of Cas variants) to improve fidelity, activity, and reduce the size (Tycko *et al*. 2016). Mining of natural CRISPR diversity has yielded new Cas enzymes with distinct biochemical properties from *S. pyogenes* Cas9, including compact (Cas12, CasΦ), thermostable, and RNA-targeting (Cas13) variants. DNA base editors (cytidine or adenine deaminases fused to nickase Cas/dCas) enable single-base transitions without double-strand breaks (Komor *et al.* 2016), and prime editors (Cas9–reverse transcriptase fusions) further allow small insertions, deletions, and substitutions with fewer undesired byproducts (Anzalone *et al.* 2019). Synthetic protein fusions of site-specific recombinases and integrases to DBDs such as dCas9 or TALES, enable targeted genomic insertions, deletions, and inversions of large DNA segments (Durrant *et al.* 2023).

As the field continues to advance at a rapid pace, several emerging directions are shaping the next generation of editors. Systematic mining efforts beyond CRISPR are uncovering diverse RNA- and protein-guided systems, many derived from transposon-encoded recombinases, such as bridge recombinases that enable programmable DNA insertions, excisions, and inversions beyond traditional Cas-based editors (Pacesa *et al*. 2024). In parallel, alternative editing modalities such as epigenome editors have yielded novel tools for locus-specific transcriptional activation and repression that bypass the need to change the genomic DNA sequence (McCutcheon *et al.* 2024). Another growing frontier is the integration of AI, where deep learning frameworks are enabling the *de novo* discovery and optimization of compact, high-fidelity nucleases, and other programmable effectors (Kim *et al.* 2025b; Ruffolo *et al.* 2025). These approaches complement computational and ML-based efforts already used to refine linker architectures, domain combinations, and off-target prediction. As genome and epigenome editing technologies enter the clinic for use in medicine, engineers are pushing to apply these systems *in vivo* in disease-relevant tissues, such as brain, liver, or stem-cell populations through evolving delivery platforms (viral, lipid nanoparticle, and synthetic vectors) (Madigan *et al*. 2023). Ongoing innovation in modular editor design continues to push the boundary of what can be genetically and epigenetically engineered, expanding the scope of programmable control in living systems.

### Synthetic protein circuits (Dongyang Li and Michael B. Elowitz^*^)

Circuits of interacting proteins sense, process, and respond to biochemical signals and environmental cues (Bray 1995). Synthetic biology seeks to engineer such circuits to program new functions in living cells (Chen and Elowitz 2021). These circuits open up new possibilities for cell-based diagnostics and therapeutics that overcome limitations of existing therapies in terms of specificity, drug tolerance, and limited dynamic range (Tan *et al.* 2021; Yin *et al.* 2025b). Additionally, protein circuits are foundational tools that reveal design principles in signaling pathways (Leopold and Verkhusha 2024), and provide a fertile ground for discovering new paradigms of biological computation (Grozinger *et al.* 2019).

An early approach for engineering protein circuits focused on rewiring existing pathways by “cutting and pasting” protein domains, and this continues to be a powerful way to create new devices. This engineering approach has been widely applied to synthetic receptors, cell adhesion molecules and intracellular signaling pathways. By recombining endogenous Notch receptor transmembrane domains with unique extracellular binding domain and transcription factors, synNotch receptors enabled the creation of novel, bio-orthogonal ligand-receptor signaling systems (Roybal *et al.* 2016; Piraner *et al.* 2025). Similarly, combining interacting extracellular and intracellular protein domains enables the construction of a repertoire of new synthetic cell adhesion molecules with various properties (Chao *et al.* 2022; Stevens *et al.* 2023). Inside cells, modular scaffold proteins and heterologous expression can steer information flow and fate decisions in the MAPK signaling response (Bashor *et al.* 2008; O’Shaughnessy *et al.* 2011; Gordley *et al.* 2016; Mishra *et al.* 2021). More recently, modular phosphorylation or protease networks assembled from kinase-phosphatase domains and protease domains have shown it is possible to engineer signal processing systems and sense-and-respond behaviors in human cells (Gao *et al.* 2018; Yang *et al.* 2025b).

Recent advances in directed evolution, *de novo*/ML-guided design, and cross-species part adaptation (e.g. CRISPR/Cas (Liu *et al.* 2021)) are now enlarging the toolkit of enzymes, binders, and scaffolds, enabling circuits with new inputs, interfaces, and control modalities. Synthetic dimerizers provide predictable, orthogonal interfaces that complement natural scaffolding domains (Thompson *et al.* 2012; Chen *et al.* 2019; Plaper *et al.* 2024). LOCKR cage/key switches (see Section *In vitro* diagnostics (Chenggang Xi and Andy Hsien-Wei Yeh^*^)) and their colocalization-dependent variants (Co-LOCKR) implement logic gating directly through designed conformational latches and proximity, enabling antigen-boolean targeting and cytotoxic control without gene regulation (Langan *et al.* 2019; Lajoie *et al.* 2020). *De novo* multi-state proteins and designed enzymes point to fully orthogonal circuits assembled entirely from engineered parts (Watson *et al.* 2023; Lisanza *et al.* 2025), enabling functions that complement or surpass natural counterparts.

Because of their fast, transient kinetics and genome-sparing operation, synthetic protein circuits are especially attractive for cell-based therapy, where precise, temporally resolved control is paramount. Increasing interest has been drawn to therapeutic circuits that couple detection of cellular states to precise interventions. This approach has been enabled by binders that target disease-associated signaling molecules (Teng *et al.* 2021). For example, therapeutic protease circuits delivered as mRNA in lipid nanoparticles can detect mutant RAS and conditionally trigger tumor-cell killing (Lu *et al.* 2025). Unlike targeted inhibitors, this approach does not rely on oncogene addiction, and appears less susceptible to resistance. Similarly, in a complementary strategy, circuits that specifically target and propagate within cancer cells with hyperactive ErbB activity, convert oncogenic RAS/ERK activity into user-defined therapeutic outputs in mammalian cells (Chung *et al.* 2019; Zou *et al.* 2026).

Mathematical modeling plays a key role in designing new functions and exploring protein circuit design spaces. *In vitro* enzymatic networks with DNA polymerase and proteases have implemented multilayer perceptrons and reservoir computation networks that showed powerful computational capability similar to their *in silico* counterparts, albeit smaller in scale (Okumura *et al.* 2022; Ghosh *et al.* 2026). *In vivo* circuits have also been shown to demonstrate interesting new functions. A non-intuitive circuit design that combined homo- and hetero-dimerizing transcription factors with dimer-dependent autoregulation enabled multistability, a key requirement for multicellular life (Zhu *et al.* 2022). Similarly, self-activating and mutually inhibiting engineered proteases can be composed into a winner-take-all classification circuit (Chen *et al.* 2024a). More broadly, modeling suggests that competitive protein–protein dimerization networks can function as powerful, fast, and flexible computers, whose behavior can be modulated by changing protein expression levels in cells (Parres-Gold *et al.* 2025). These and other results highlight the computational potential of engineered protein circuits.

**Figure 34:**
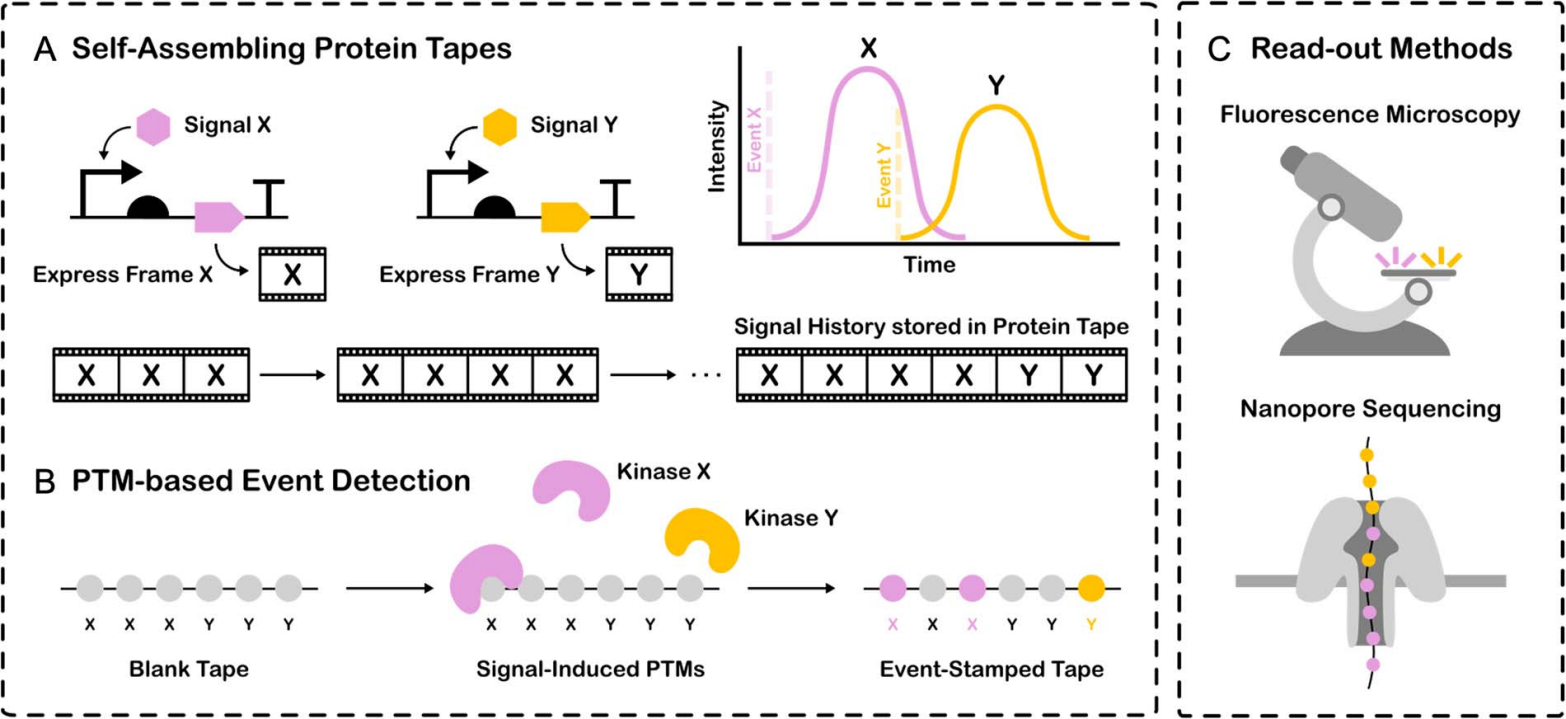
Overview of protein-based recording technologies and detection methods. (A) Self-assembling protein tapes: Signals X and Y drive expression of distinct peptide monomers that co-assemble into linear “tapes,” with the ordered pattern of X/Y units encoding the timing and frequency of upstream events. (B) PTM-based event detection: A constitutive “blank” protein substrate is differentially modified by signal-dependent enzymes (e.g. kinases, acetyltransferases, and other PTM writers), depositing position-specific marks that encode event identity and timing. Histories stored in these protein substrates can be read out by fluorescence or by sequence-level methods such as nanopore protein sequencing or targeted proteomics. (C) Detection methods commonly used in conjunction with protein-based recording methods.

Looking ahead, computation—especially ML-guided protein design and engineering—are poised to expand protein circuit design capabilities. These approaches allow *de novo* design of heterodimers, high-affinity binders, multi-state switches, and allosteric enzymes. Data-efficient protein language model parameterization will continue to expand the parts catalog and accelerate optimization (Watson *et al.* 2023; Jiang *et al.* 2025; Pacesa *et al.* 2025). Broadening modalities beyond proteolysis/phosphorylation (e.g. ubiquitin/SUMO cycles, acetylation/methylation, split inteins) and integrating protein circuits with DNA/RNA/metabolic layers will yield more versatile and robust controllers (Qian *et al.* 2022b; Kaseniit *et al.* 2023; Hua *et al.* 2025). These modalities should also offer precise readouts and perturbations for applications. At the circuit level, a challenge will be to engineer versatile, context-dependent architectures that reuse the same components yet alter behavior with local cues—cell type, ligand stoichiometry, subcellular localization/condensate state, or mechanical context—so a single design can switch between different functions without re-engineering parts. Finally, for translational applications, humanizing protein circuits will be critical for minimizing immunogenicity *in vivo*. Based on the growing ability to engineer precise, robust, and powerful protein circuits, we anticipate growth in this area in the years ahead.

### Protein recorders (Kingsley L.-J. Wong, Mattias Tolhurst, and Jeff Nivala^*^)

Molecular recording aims to capture biological events directly into biomolecular substrates (Fig. 34). Early systems have primarily encoded information in DNA *via* CRISPR-based writers (see Section Genome and epigenome engineering (Izaiah J. Ornelas and James K. Nuñez^*^)), recombinases, or base editors, leveraging stable, heritable edits as a durable log (Shipman *et al.* 2016; Choi *et al.* 2022; Lear and Shipman 2023). Proteins offer a complementary substrate with a larger “alphabet” (20+ residues and diverse PTMs) and richer, tunable chemistry. In protein-based recorders, “writing” can occur by permanent sequence changes, site-specific PTMs, or ordered assembly of modular peptide units; “reading” can be performed with mass spectrometry, fluorescence-based reporters, or, more recently, nanopore protein sequencing. Protein recorders promise higher temporal resolution and direct coupling to cellular physiology, enabling *in vivo* histories of single cells, such as normal state transitions, stimulus responses, and disease progression, while avoiding genomic perturbations inherent to DNA writing. Together, these approaches outline a toolkit that trades DNA’s archival stability for protein’s immediacy and versatility, opening space for recorders tailored to specific time scales, signals, and readout modalities.

**Figure 35:**
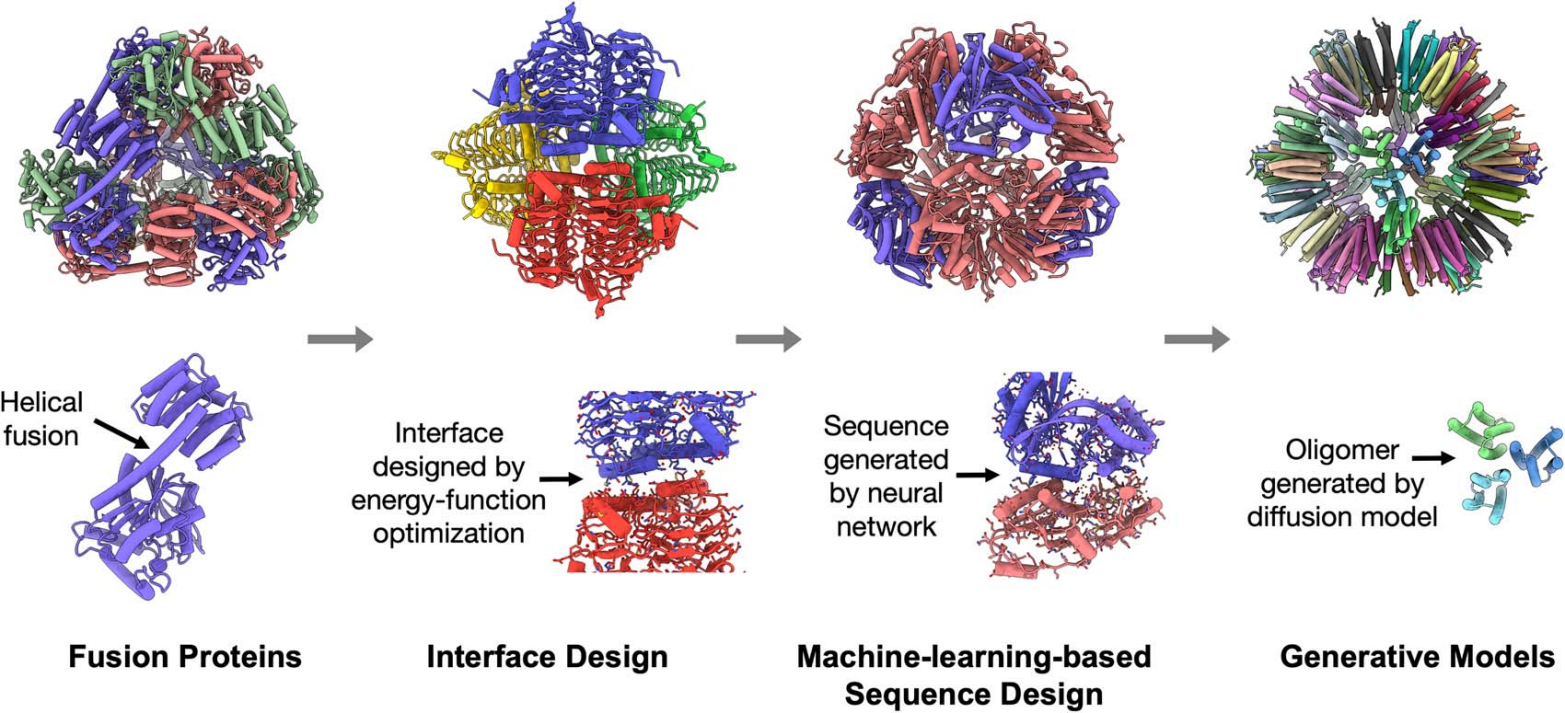
Advances in designing protein cages. Early designs relied on fusion proteins that combined oligomeric building blocks to enforce defined symmetries (Padilla *et al*. 2001). Later approaches used interface redesign to control the geometry and specificity of subunit interactions (King *et al.* 2012). With the advent of ML-based sequence design methods, such as ProteinMPNN, success rates, and structural diversity increased dramatically (de Haas *et al.* 2024; Meador *et al.* 2024). Today, generative models like RFdiffusion and Chroma (Ingraham *et al.* 2023; Watson *et al.* 2023) enable the creation of entirely new folds and architectures, expanding the design space of protein complexes.

Early protein recorders have achieved continuous, time-resolved logging by coupling a promoter of interest to the expression of engineered peptide monomers that co-assemble into elongated “protein tapes” (Lin *et al.* 2023; Linghu *et al.* 2023). The sequence of incorporation encodes event timing and frequency, while embedded fluorescent tags or dye-binding motifs enable optical readout of order and intensity. These proof-of-concept studies have used induced transcription to write over ~1–7 days, highlighting feasibility but also current limits on duration and signal types. Expanding beyond transcriptional inputs to linkers responsive to protease activity, metabolites, or specific signaling pathways could broaden what is recordable and extend timescales with more stable scaffolds. In parallel, a kinase-based recorder has demonstrated reversible phosphorylation of a modular protein substrate, suggesting an alternative PTM-centric strategy that may generalize to other modifications and support erasable or analog-like recording modes *in vivo* (Sun *et al.* 2025).

As protein recorders advance, two levers matter most: how we write and how we read. Current self-assembling tapes have proved the concept but use bulky monomers (>500 aa), assemble slowly (hour-scale resolution), and depend on fluorescence, which constrains multiplexing and density. Shifting readout from photons to sequences, e.g. *via* nanopore protein sequencing or targeted proteomics, would unlock the full amino-acid/PTM alphabet, dramatically increase data density, and enable error-corrected, multiplexed decoding. On the writing side, faster assembly chemistries (e.g. split-intein ligation (Carvajal-Vallejos *et al.* 2012), enzyme-catalyzed peptide modification or bond formation, and tag/catcher pairs (Cai *et al.* 2025)) could push resolution toward minutes while keeping background low. Design goals include smaller, orthogonal monomer units; catalytic or autocatalytic propagation for speed; embedded synchronization/barcode motifs for alignment; and calibrated error models to support reliable decoding at cellular scale. Together, these advances would hint that protein-native writers paired with sequence-level readers could evolve from simple timelines into richly multiplexed, proteome-scale logs, ultimately opening new ways to ask (and maybe answer) questions we do not yet know how to frame.

## Materials, nanostructures, and machines

### Cages and scaffolds (Roger Castells-Graells and Todd O. Yeates^*^)

Designed protein cages, or nanohedra, are self-assembling protein architectures that form geometric shells typically resembling the Platonic solids: tetrahedron, cube, octahedron, icosahedron, and dodecahedron. Their inherent symmetry, defined size, modularity, and biocompatibility make them attractive scaffolds for diverse applications in nanomedicine, materials science, and structural biology. The field originated in the early 2000s, when Padilla *et al.* (2001) designed the first protein cage by helical fusion of trimeric and dimeric oligomers, demonstrating that geometric design principles could be used to create complex self-assembling protein architectures (Fig. 35). That study laid out general principles of symmetry, which framed further developments in the field, as has been previously summarized (Yeates 2017). The key idea was that a surprisingly diverse range of architectures can be realized by bringing together just two separate symmetric components in precise configurations. While the original approach of Padilla *et al*. (2001), further demonstrated by Lai *et al.* (2014), relied on helical fusions between oligomers, a decade later King *et al.* (2012) exploited newly available protein–protein interface design algorithms as a second method for bringing multiple symmetric oligomers together in precise configurations to create protein cages. The power of computational interface design made it possible to create larger sets of designed cages with diverse architectures, including icosahedra, the highest symmetry form possible (Bale *et al.* 2016). In recent work, protein cages or capsids of even larger size have been realized by computational methods that allow for symmetry-breaking (Dowling *et al.* 2025).

Recent advances in AI and the introduction of ML-based sequence design methods such as ProteinMPNN (Dauparas *et al.* 2022) have markedly increased the success rate of *de novo* cage designs (de Haas *et al.* 2024; Meador *et al.* 2024). Designed protein cages now serve as highly versatile platforms for antigen presentation and vaccine development where multivalent display enhances immune activation (Walls *et al.* 2020); for targeted drug and gene delivery (Edwardson *et al*. 2018); and for enzyme co-localization (McConnell *et al.* 2020). Furthermore, they have been employed as imaging scaffolds in cryo-EM to visualize small proteins at high resolution (Cannon *et al*. 2019; Edwardson *et al.* 2022; Castells-Graells *et al.* 2023). Recent advances have also yielded ligand-responsive cages capable of controlled conformational switching, marking a shift from static shells to dynamic, stimulus-sensitive nanoparticles (Lee *et al.* 2024).

Looking ahead, generative AI algorithms for creating (or “hallucinating”) novel protein backbones, such as RFdiffusion (Watson *et al.* 2023) and Chroma (Ingraham *et al.* 2023), are transforming protein design by taking the field beyond the scope of natural protein structures. The vastly expanded protein structural space should pave the way for programmable nanomachines, protein systems capable of conformational switching, that will bridge the gap between static materials and active biological systems. The integration of AI-driven tools with geometric design principles is transforming the landscape of designed protein cages. The possibilities for new architectures and functions will continue to expand, driving breakthroughs in protein engineering across biomedicine and nanomaterials.

### Hydrogels (Ruiyan Gao and Diego López Barreiro^*^)

**Figure 36:**
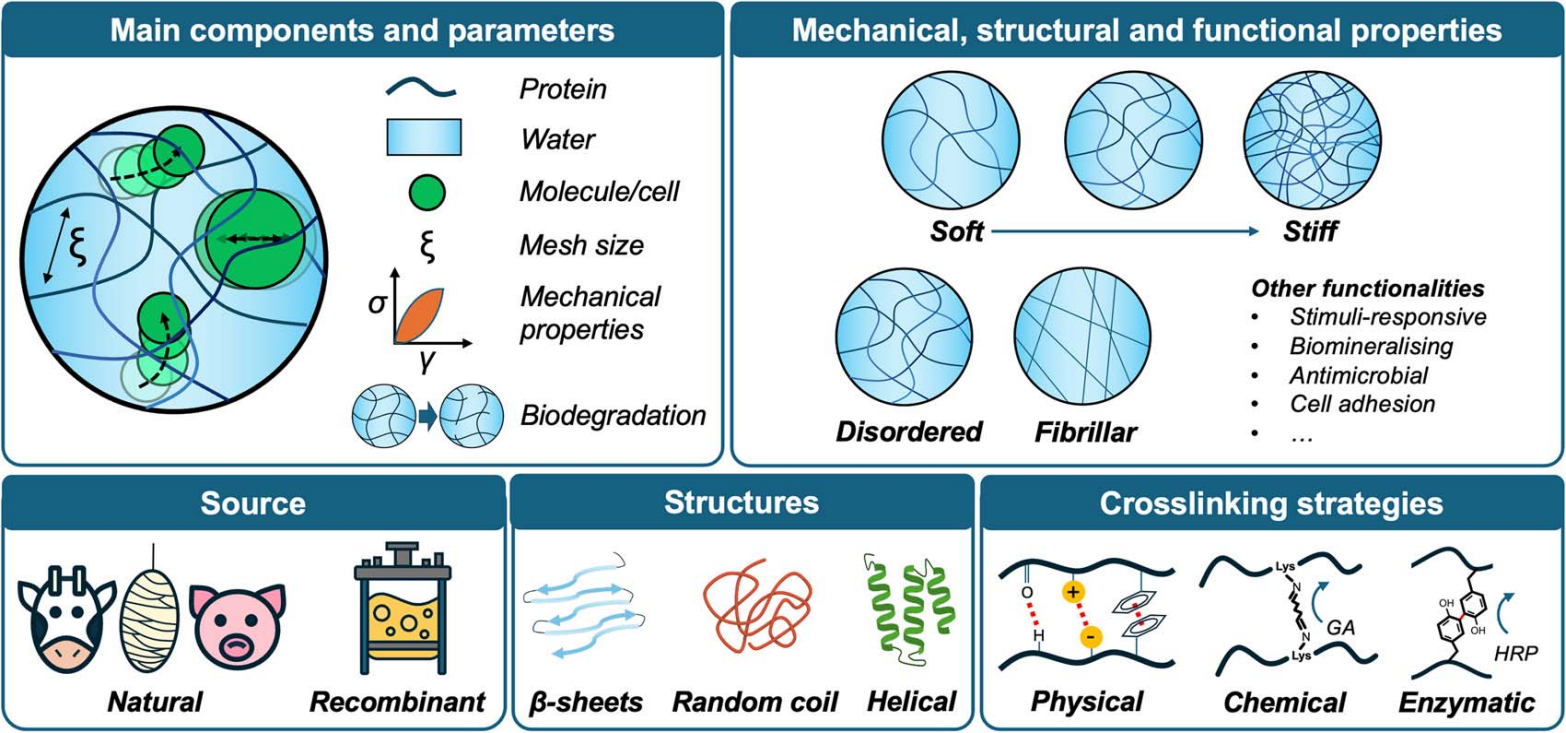
The components, parameters, and properties of protein hydrogels. Proteins can be sourced from natural sources or recombinantly produced, and form specific secondary structures (e.g. β-sheets, random coil, or helical) that affect the mechanical and structural properties of the resulting hydrogels. Other parameters influencing the properties of hydrogels include the crosslinking strategy (physical, chemical, or enzymatic), the protein concentration, or the protein tendency to form disordered or fibrillar structures. Additionally, proteins can display additional non-structural functionalities (e.g. stimuli-responsiveness, biomineralisation, or antimicrobial features) that expand the properties of hydrogels. GA: Glutaraldehyde; HRP: Horseradish peroxidase.

Hydrogels are water-swollen polymer 3D networks (Peppas *et al.* 2006) whose viscoelastic and structural properties can be tuned *via* physical, chemical, or enzymatic crosslinking (Fig. 36). Given the similarities between hydrogels and the extracellular matrix (ECM), natural structural proteins such as collagen, elastin, keratin, and silk fibroin (Zhang *et al.* 2023) have been intensively researched to produce biocompatible and biodegradable ECM-mimetic hydrogels with a range of mechanical and structural properties for tissue engineering or drug delivery applications, to name a few (Morin *et al*. 2006; Ham *et al.* 2016; Tian *et al.* 2022b; Fernández-Serra *et al.* 2024). Other natural non-structural proteins, such as albumin, serum proteins or lysozyme have also been formulated into hydrogels (Baler *et al.* 2014; Seth *et al*. 2023).

Nonetheless, natural proteins face limitations including source-to-source variability, immunogenicity, presence of contaminants, and inefficient extraction/harvesting processes. This can lead to limited control over the resulting hydrogel structure and compromise performance (Chang *et al*. 2021). Fortunately, advances in protein engineering and synthetic biology have propelled the development of new nature-inspired recombinant proteins (Miserez *et al*. 2023). This allows us to precisely design new modular proteins that combine tandem repeats of natural structural proteins—for instance, thermoresponsive VPGXG elastin-like and stiff GAGAGS silk-like blocks (Huang *et al.* 2016a). Besides structural blocks, we can also fuse functional peptides (e.g. cell binding, biomineralisation, antimicrobial) (Shire *et al.* 2024). Overall, this allows us to introduce programmability and create hydrogels that self-assemble into specific structures (e.g. β-sheets, disordered or helical), exhibit stimuli-responsiveness or display multifunctionality.

However, the vast design space for new recombinant protein is challenging to explore *via* experimentation alone. Fortunately, data-driven approaches relying on molecular dynamics simulations or ML (Huang *et al.* 2016a; Ni *et al.* 2023; Mout *et al.* 2024; Lu and Buehler 2025) are significantly accelerating the speed at which new sequences can be tested *in silico* for specific design objectives (e.g. secondary structure content, shape) or material properties (e.g. strength, stimuli-responsiveness). Here, improved and more consistent data reporting and sharing strategies across groups working on protein hydrogels will be crucial to accelerate progress and develop generalisable predictive tools that work across a wide range of proteins. New recombinant proteins can also be used to produce bioinks for the 3D printing of hydrogels with novel structures, compositions, and functionalities (Salinas-Fernández *et al.* 2020). However, further research is needed to improve aspects such as bioink stability, printing resolution or vascularization (critical for tissue engineering applications). Finally, protein hydrogels are playing a critical role in the emerging field of engineered living materials (Liu *et al.* 2022). Such hydrogels are typically either secreted by cells (Wang *et al.* 2021b) or made from natural proteins (Zhao *et al.* 2019). In engineered living materials, cells and hydrogel scaffolds synergistically interact to assemble materials with enhanced dynamic and stimuli-responsive properties, and with applications including healthcare, biomineralization, biosensing or environmental applications (Zhao *et al.* 2019; Tang *et al.* 2021).

### Nanopores (Yujia Qing^*^ and Hagan Bayley^*^)

**Figure 37:**
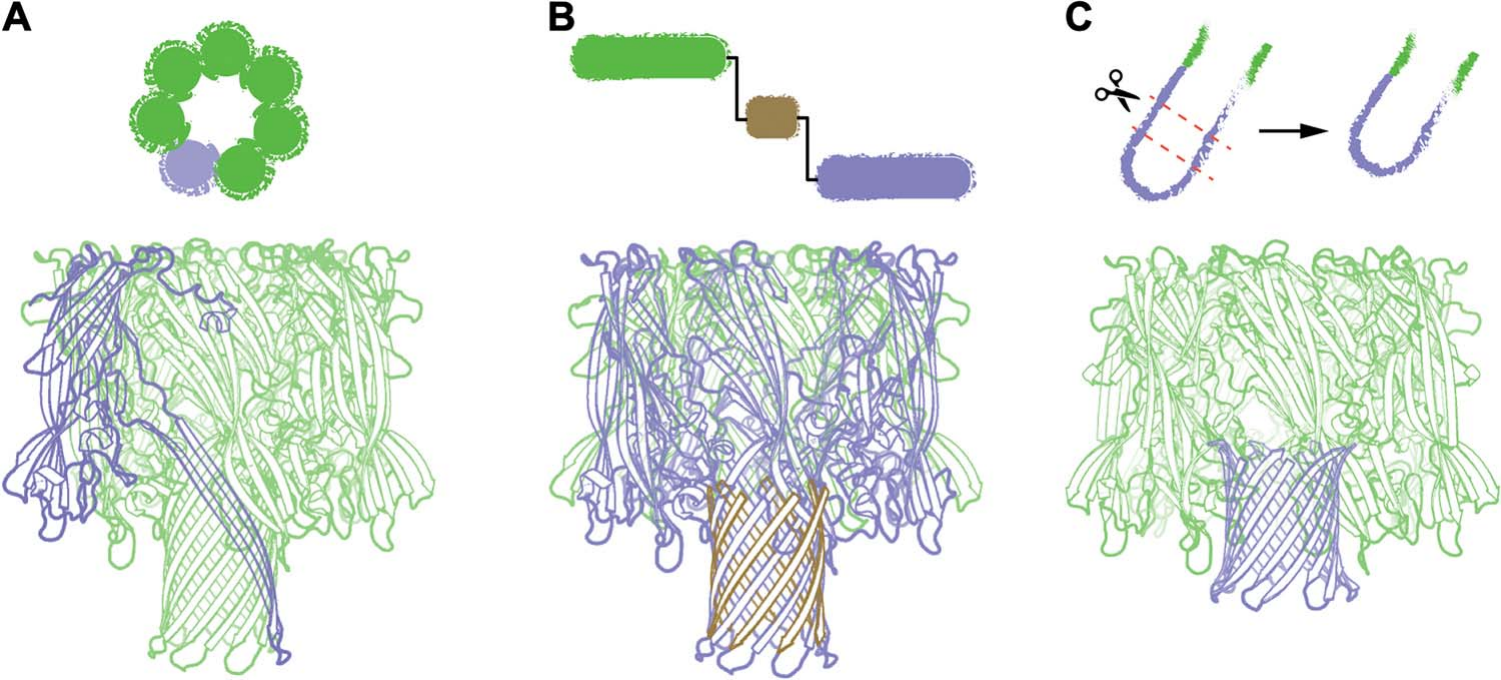
Engineered protein nanopores. Representative engineering strategies applied to the α-hemolysin nanopore (PDB ID: 7AHL) (Song *et al.* 1996). (A) Incorporation of a single variant protomer (purple) within a heptamer. (B) Semisynthesis by native chemical ligation of the expressed C- and N-terminal fragments (green and purple, respectively) with a central synthetic peptide (brown). (C) Barrel truncation by pairwise removal of amino acids from both transmembrane β strands within individual subunits.

Pore-forming proteins, which assemble into nanoscale transmembrane channels to mediate ionic or molecular transport, were proposed as tools for biotechnology in the 1970s. Like enzymes and antibodies, they possess robust 3D structures able to scaffold molecular interactions. Early work focused on the chemical control of pore assembly (Walker and Bayley 1994; Walker *et al.* 1994, 1995). Later, engineering was mostly directed toward single-molecule sensing, where individual analytes translocating through a nanopore under an applied potential modulate transmembrane ionic currents. Most nanopore sensors are multi-subunit bacterial toxins (Fig. 37); the protomers can be engineered to alter the structure and function of the assembled pores. Sensing requires bespoke nanopores featuring interaction sites for target molecules—allowing transient non-covalent binding or covalent attachment (Shin *et al.* 2002)—or reading head(s) positioned to register segments of translocating polymers (Stoddart *et al.* 2010). Such interactions must be brief yet long enough for accurate signal acquisition, optimally in milliseconds. Additional engineering aims to tune the electroosmotic (Gu *et al*. 2003) and gating properties (Wang *et al.* 2025) of pores. Further, engineered nanopores underpin research into single-molecule chemistry (Qing *et al.* 2020), inter-compartment communication (Booth *et al.* 2019), and directed molecular assembly (Koo *et al.* 2019).

In 1996, the crystal structure of *S. aureus* α-hemolysin (αHL) was resolved at 1.9 Å, the first transmembrane β-barrel protein formed by multiple subunits to be visualized (Song *et al.* 1996). Since then, almost all known means of protein engineering have been applied to nanopores. Site-directed cysteine mutagenesis and subsequent chemical modification (Walker and Bayley 1995a; Valeva *et al.* 1996) remains routinely practiced today. Functional pores have been formed by complementing fragments with overlaps, nicks, and gaps (Walker *et al.* 1993), and the cavity within the pore has been filled with polymers or small proteins (Howorka *et al.* 2000; Jung *et al.* 2005). Nanopores have also been covalently functionalized with ligands—tethered by chains or as extensions of the polypeptide chain—for protein recognition (Movileanu *et al.* 2000; Cheley *et al*. 2006; Harrington *et al.* 2013, 2015), oligonucleotides for binding complementary sequences (Howorka *et al.* 2001a, Howorka *et al*. 2001b), aptamers (Rotem *et al.* 2012) and host molecules (Wu *et al.* 2007; Clarke *et al.* 2009) for capturing cognate guests. Approaches originated in the nanopore field include scanning mutagenesis combined with chemical modification (Walker and Bayley 1995b); the incorporation of non-covalent adapters (e.g. cyclodextrins) (Gu *et al.* 1999; Gu *et al*. 2001a, 2001b) or carriers (Braha *et al.* 2005); the making of heteromers containing one or more differing subunits (Braha *et al.* 1997; Banerjee *et al.* 2010; Hammerstein *et al*.y 2011); and the identification of related pores with alternative subunit stoichiometry (Miles *et al*. 2002; Jayasinghe and Bayley 2005). Critical to the efficient screening of engineered variants was the development of cell-free synthesis of nanopores and associated functional assays (Walker *et al.* 1992), which in turn confirmed the heptameric nature of the αHL pore (Gouaux *et al.* 1994; Cheley *et al*. 2002). Years of experimentation revealed that β-barrels are unexpectedly tough (Kang *et al.* 2005) and amenable to drastic modification (Maglia *et al.* 2009), laying the foundation for their use as molecular sensors in portable devices. The recent surge in AI-driven protein design, powered in part by the data generated by decades of nanopore engineering, is transforming what can be created. Yet realizing the full potential of AI will require faster and more scalable screening to turn designs into pores with unprecedented functional properties.

Nanopore engineering is entering a phase defined less by incremental, single-site tinkering but more by architectural exploration and multi-site modification to generate complex functions. Emerging directions include the truncation (Stoddart *et al.* 2014) and elongation of nanopores (unpublished work from the Qing group), as well as the building of tracks for walking or hopping that guide molecular motion with sub-nanometer precision (Qing *et al.* 2018). Advances in nanopore semi-synthesis by native chemical ligation (Lee and Bayley 2015) (see Section Protein ligation (Luis F. Guerra and Manuel M. Müller^*^)) opens the possibility of precise, multisite functionalisation within individual pores, while the integration of nanopores into multi-domain complexes (e.g. with unfoldases or helicases) (Zhang *et al.* 2021) can couple biopolymer translocation with sequence analysis. Further, the principles uncovered here are likely to inform the construction of nanopore arrays for future biomedical and bioelectronic applications.

### Artificial protein motors (Vanessa Tanja Trossmann and Birte Höcker^*^)

**Figure 38:**
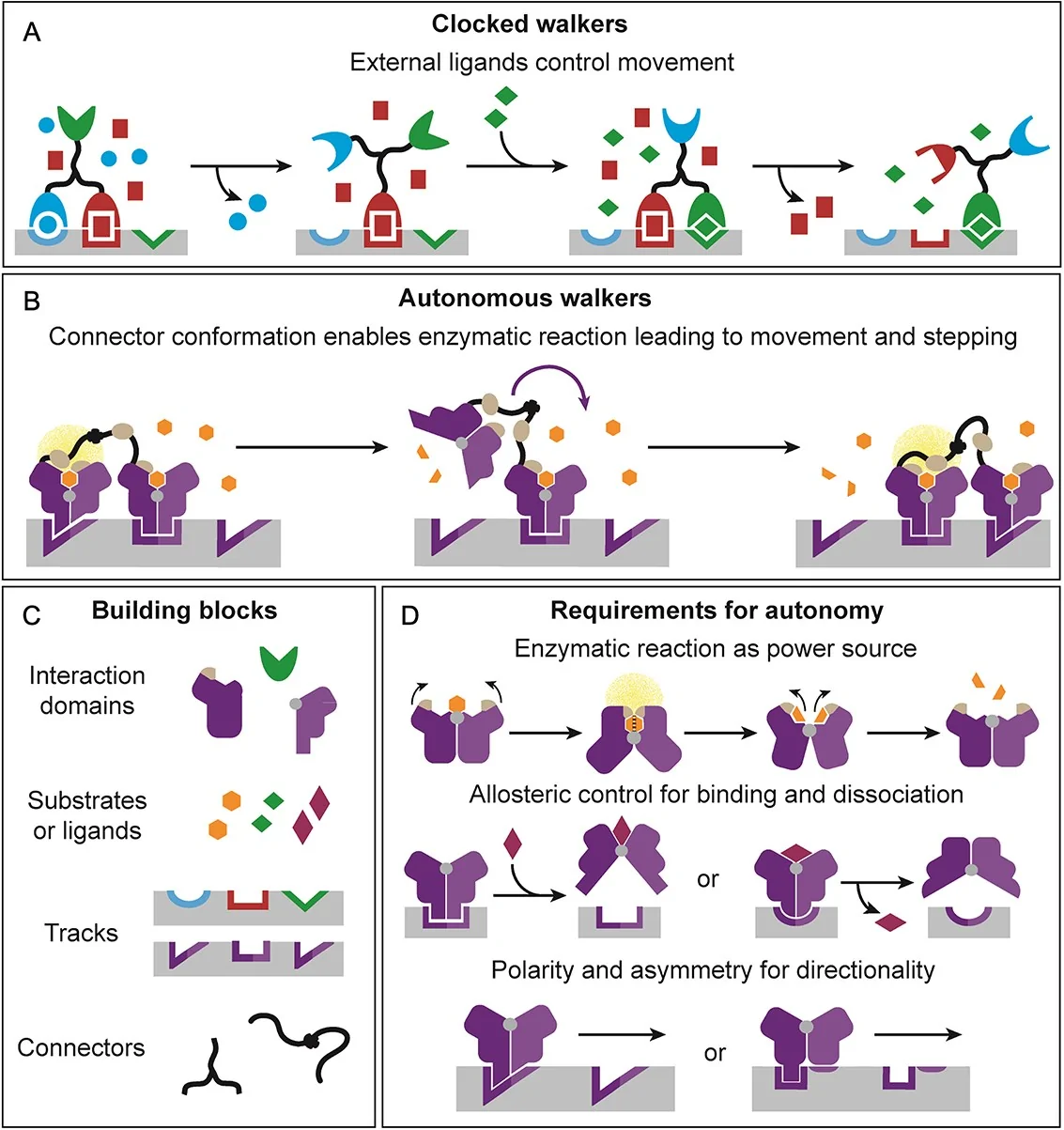
Bottom-up strategies for artificial motor protein design. (A) Clocked walkers rely on external ligand control to coordinate their association, dissociation, and stepping along a track: The design illustrates a clocked walker consisting of three orthogonally connected interaction domains (blue, red, green). Directional movement along an asymmetric track is achieved by external ligand control (blue circles, red squares, and green diamonds) leading to track association and dissociation (Nilsson *et al.* 2025). (B) Autonomous walkers use chemical energy for motion, force generation and stepping without external control: The exemplary design illustrates an autonomous walker consisting of two linked interaction domains (purple) exhibiting an enzymatic reaction (yellow) consuming a substrate (orange) when connectors (black) are in a conformation to complement the active site (beige). Chemical conversion, product release, and conformational changes lead to dissociation from the asymmetric track and directional movement (purple arrow). Binding of a new substrate molecule results in track association by allosteric control and conformational re-arrangement of the connector restores the enzymatic functionality (yellow) finally leading to stepping. (C) Modular building blocks for generating artificial protein walkers comprise specific interaction domains, their appropriate substrates and/or ligands, their corresponding tracks for binding as well as mechanically adjustable connectors. (D) Generation of autonomy in artificial protein walkers contain three design challenges: Implementing an enzymatic cycle as a power source, enabling allosteric communication between distant protein sites to control binding and dissociation, and establishing polarity and asymmetry for directional movement (Nilsson *et al.* 2024).

Movement is essential to life. It is driven by biomolecular motors—complex protein assemblies that convert chemical energy into mechanical work—that perform key cellular functions through linear or rotary motion. Linear motors such as myosin, kinesin, and dynein move efficiently along actin filaments or microtubules. Although key principles of motor function are known, their complexity makes it hard to dissect mechanisms, motivating efforts to build motor functions from bottom up (Fig. 38) (Linke *et al.* 2020). Directional movement typically involves three steps: binding to a track, a conformational change (often triggered by ATP hydrolysis) and dissociation (Furuta *et al.* 2017). The conformational change shifts a structural element producing a step whose direction depends on motor geometry or track asymmetry (Zuckermann *et al.* 2015). Processive motors like kinesin remain attached as they move, while non-processive motors like myosin II detach after each cycle but work in bundles of short, rapid interactions.

Design strategies of artificial protein motors distinguish between clocked and autonomous walkers. Clocked walkers rely on an external ligand to control their association, dissociation, and stepping along the track (Fig. 38A). In contrast, autonomous walkers use chemical energy for efficient motion, force generation, and stepping without external control (Fig. 38B). An example for a synthetic clocked walker is the Tumbleweed, an artificial motor with three coiled-coil legs each containing different DNA-binding domains that interact with an asymmetric DNA-track. Its stepping is controlled by ligand pulses, and it follows a Brownian ratchet mechanism as shown by SPR and single-molecule fluorescence studies (Nilsson *et al.* 2025). Zuckermann *et al*. modeled a synthetic kinesin-inspired protein (SKIP), which has not been experimentally implemented yet, where directional motion along a symmetric track arises from chemical potential and persistence under a temporal symmetric cycle, without requiring spatial, or temporal asymmetry or a power stroke (Zuckermann *et al.* 2015). Furuta *et al*. built autonomous hybrid motors combining a motor part of dynein with actin-binding elements that could slide directionally along actin filaments using ATP hydrolysis (Furuta *et al.* 2017). They later paired dynein with nonpalindromic DNA-binding proteins to create fast motors that glide along DNA nanotubes, with directionality set by spatial arrangement of binding sites (Ibusuki *et al.* 2022). Another synthetic autonomous motor, the Lawnmower, comprises a spherical hub decorated with trypsin proteases that moves by proteolytic cleavage of a peptide “lawn.” This burnt-bridge Brownian ratchet produces biased, directional motion to unvisited substrate with a speed up to 80 nm/s (Korosec *et al.* 2024).

A general roadmap for creating artificial autonomous protein-based molecular motors from modular building blocks (Fig. 38C) has been provided by Nilsson *et al.* (2024). Three main design challenges were identified: implementation of an enzymatic cycle as a power source, enabling allosteric communication between distant protein sites, and establishing polarity for directed movement (Fig. 38D). Modular strategies can leverage both natural non-motor protein components and *de novo* design. Indeed, biomolecular motors have become a goal in *de novo* protein design. For example, Courbet *et al.* used computational design to construct mechanically constrained axle-rotor protein assemblies (Courbet *et al.* 2022). These components, designed with internal dihedral or cyclic symmetry, assemble into complexes that adopt multiple rotational states, which is a step toward rotary nanomachines. More recently, Ulčakar et al. reported the *de novo* design of random protein walkers built from homo-oligomers with heterodimeric feet that diffuse along designed fibrous protein tracks (Ulčakar *et al.* 2025). Although critical challenges such as achieving directionality and controlled movement remain, these studies mark important progress toward bottom-up protein engineering and design of artificial motor proteins for future applications in nanomedicine, material sciences, biocomputing, and synthetic biology (Furuta 2023).

## Synthetically augmented proteins

### Non-canonical amino acids (James A. Van Deventer^*^)

Nature uses templated protein biosynthesis to solve a tremendous range of biomolecular problems. However, nature also routinely employs more than the 20 canonical amino acids (cAAs), decorating proteins with hundreds of PTMs, and even genetically encoding two additional amino acids, selenocysteine and pyrrolysine (Peeler and Weerapana 2019; Kivenson *et al.* 2025). Genetic code manipulation with noncanonical amino acids (ncAAs) takes nature’s concepts a step further to enable precise chemical augmentation of proteins in the laboratory (ncAAs are also referred to as UAAs, nonnatural amino acids, nonstandard amino acids, and nonproteinogenic amino acids). Applications of ncAA-containing proteins are found in fields including structural biology, mechanistic biochemistry, proteomics, drug discovery, biomaterials, and more. Recent publications have exhaustively reviewed ncAAs in various fields (Budisa 2025); this short section cannot possibly do this! Instead, this section summarizes principles of ncAA incorporation, selected application areas, and opportunities for further advancement.

**Figure 39:**
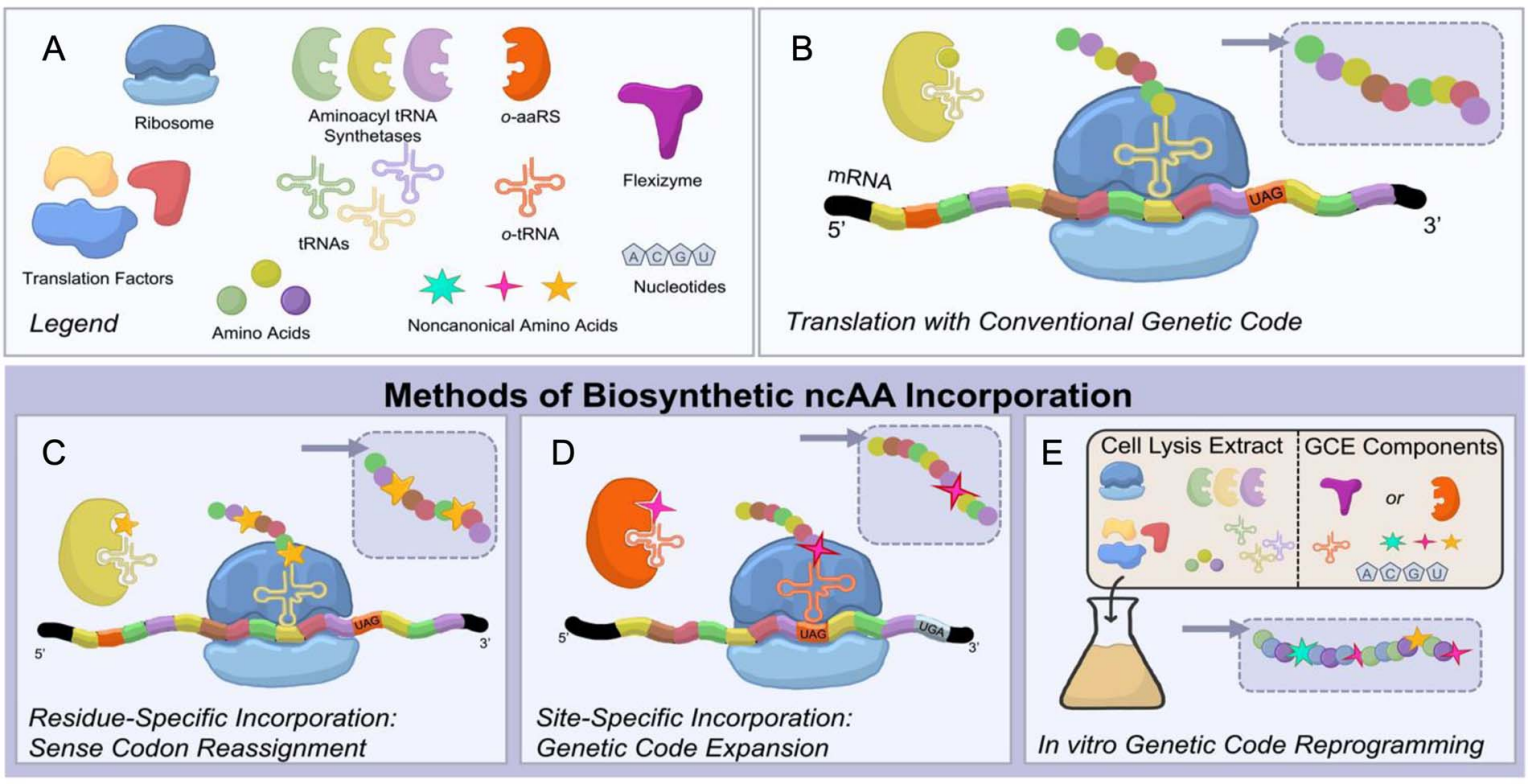
Elements and applications of genetic code manipulation systems. (A) Biomolecules used during translation of canonical amino acids (cAAs) and noncanonical amino acids (ncAAs). O-aaRS and o-tRNA: The orthogonal aaRS and tRNA that comprise an OTS used for genetic code expansion. Flexizyme: A ribozyme capable of acylating tRNAs with ncAAs (for use in *in vitro* genetic code reprogramming). (B) Schematic of conventional protein translation. An aaRS aminoacylates a cognate tRNA with a specific cAA. At the ribosome, the resulting cAA-tRNA is translated based on the binding of the tRNA anticodon to the corresponding codon in mRNA. Sequentially, cAAs are added to the growing polypeptide chain until a stop codon is reached, at which point termination machinery releases the polypeptide from the translation apparatus. (C–E) Major genetic code manipulation systems. (C) Sense codon reassignment (residue-specific ncAA incorporation). Native tRNAs that normally code for a canonical amino acid are instead acylated with ncAAs; this can be achieved by native or engineered aaRSs. Resulting ncAA-tRNAs are translated at the ribosome, resulting in ncAA incorporation in response to the group of sense codons encoding a specific cAA. Notable applications: Structural biology, proteomics, biomaterials, protein stability. (D) Genetic code expansion in response to a “blank” codon (site-specific ncAA incorporation). An OTS is introduced into cells, where the o-aaRS aminoacylates the o-tRNA with an ncAA. At the ribosome, the ncAA-tRNA is decoded based on o-tRNA anticodon identity (usually in response to a stop codon). Notable applications: Dissecting effects of PTMs, characterizing membrane proteins, enzyme engineering, antibody engineering. (E) *In vitro* genetic code reprogramming. The biomolecular components required to define a reworked genetic code are combined with components of the translation apparatus. Cell-free translation reactions result in the production of polypeptides which may contain numerous distinct ncAAs (or other noncanonical monomers). Notable applications: Drug discovery, investigating translational properties of non-α amino acids. Figure adapted with permission from Lino *et al.* (2024). Copyright 2024 American Chemical Society.

There are three major types of ncAA incorporation (Fig. 39) (Lino *et al.* 2024). Acylation of specific tRNAs with ncAAs is essential for all incorporation strategies (Fig. 39A). NcAA-tRNAs then participate in ribosomal protein translation (Fig. 39B) to achieve genetic code manipulation (Fig. 39C–E). Two genetic code manipulation strategies are practiced in cells: sense codon reassignment (Fig. 39C) and genetic code expansion (Fig. 39D). *In vitro* genetic code reprogramming is practiced using cell-free protein synthesis (Fig. 39E). Sense codon reassignment, also called residue-specific ncAA incorporation, results in replacement of a cAA with a ncAA during protein translation (Fig. 39C). This technique can be simple to implement: close structural analogs of cAAs can be incorporated into proteins under standard expression conditions with little to no cell strain engineering. The earliest successful application of this technique is the replacement of methionine with selenomethionine to solve protein structures *via* X-ray crystallography (Hendrickson *et al*. 1990). The heavy metal selenium facilitates multiwavelength anomalous dispersion, addressing the phasing problem of X-ray diffraction. Hundreds of protein structures have been solved using selenomethionine, making this arguably the most impactful application of ncAAs in any field. The recognition that overexpressed or engineered aminoacyl-tRNA synthetases (aaRS) broaden the ncAAs available for residue-specific incorporation has led to further applications. Sense codon reassignment results in large compositional changes in proteins, which has been used to engineer protein-based biomaterials and investigate protein stability (Majekodunmi *et al*. 2024). In addition, residue-specific incorporation results in proteome-wide changes to newly synthesized proteins. Bioorthogonal noncanonical amino acid tagging and related techniques use ncAAs with unique reactive handles to selectively modify newly synthesized proteins, followed by proteomics or imaging workflows to identify what (or where) proteins are being synthesized (Loynd *et al.* 2025). These approaches offer unique capabilities for understanding proteomic responses to diverse biological stimuli. Sense codon reassignment supports powerful, but relatively simple, ncAA incorporation approaches that continue to impact multiple fields.

Genetic code expansion, also referred to as site-specific ncAA incorporation, uses “blank” codons to add an amino acid to the genetic code (Fig. 39D; usually a stop codon). Genetic code expansion relies on orthogonal translation systems (OTSs; also referred to as orthogonal pairs) consisting of an additional aaRS and tRNA. NcAA-tRNAs must be generated at sufficiently high rates, be compatible with the ribosome, and outcompete natural termination of translation. Drawing upon (1) fundamental biochemical characterizations of aaRS-tRNA pairs and (2) biomolecular engineering of OTSs, researchers have reported OTSs that support ncAA incorporation in prokaryotic cells, eukaryotic cells, and even whole organisms. Adding an OTS to a cell can be sufficient to produce protein containing an ncAA, but it does not guarantee efficiency. Researchers have investigated numerous approaches to improve efficiency, ranging from advanced OTS engineering to preparing genomically recoded organisms to use codons solely to encode for ncAAs (Lino *et al.* 2024; Grome *et al.* 2025).

Improved tools for genetic code expansion have led to applications in diverse fields. For example, access to encoded PTMs (including phosphorylated amino acids, acetyllysine, and sulfotyrosine) have led to insights into the molecular consequences of PTMs. This includes (1) how PTMs alter protein–protein interaction networks in cells; and (2) identification of specific modification sites that drive large changes in protein–protein interaction affinities (Allen *et al.* 2024; Wanka *et al.* 2024). NcAAs containing crosslinkable groups, bioorthogonal reactive groups, spectroscopic probes, and more, have revealed important structural and dynamic properties of membrane proteins, which remain difficult to study with standard techniques (De Faveri *et al.* 2024). Enzyme engineering with ncAAs has flourished in recent years: impressive chemical intuition and advances in directed evolution have resulted in ncAA-enzymes that operate *via* mechanisms with no known precedent in nature (Birch-Price *et al.* 2024). These compelling examples are representative of a broader set of applications achieved with genetic code expansion.

*In vitro* genetic code reprogramming (Fig. 39E) enables dramatic reworking of the genetic code. Cell viability does not need to be maintained *in vitro*, and ncAA-tRNAs can be added directly to translation mixtures in virtually limitless combinations. This enables simultaneous reassignment of many codons, although it can be technically demanding. *In vitro* genetic code reprogramming supports powerful approaches to peptide-based drug discovery: in combination with mRNA display (see Section Continuous protein evolution (Chang C. Liu^*^)), it is possible to screen billions to trillions of “druglike” peptide candidates in search of therapeutic leads (Colas *et al*. 2024). This has spurred rapid adoption of the technique, especially in industrial settings. The flexibility of *in vitro* genetic code reprogramming offers unique capabilities and applications that would be virtually impossible to conduct in cells.

What are the most exciting future opportunities for ncAAs? In some cases, important technical challenges remain. For example, not all ncAAs are readily transported across cell membranes, limiting protein yields. Two potential emerging solutions are: (1) engineering cells that biosynthesize their own ncAAs (Allen *et al.* 2024); and (2) engineering amino acid transporters to facilitate more efficient ncAA transport across membranes (Iype *et al.* 2025). A more general challenge is how to work with the growing number of large datasets that may further inform technical advances with ncAAs. These datasets range from results of high throughput laboratory experiments with ncAA-containing proteins to genome sequences of organisms that naturally utilize ncAAs (Kivenson *et al.* 2025). Careful application of tools from bioinformatics, ML, and computational modeling provides opportunities to identify and overcome technical barriers.

What about advancing applications of ncAAs? Creative discovery tools are emerging in multiple areas. Within targeted protein degradation, ncAA-based approaches are enabling target validation methods that do not require any target-specific small molecules (Shade *et al.* 2025; Xiao *et al.* 2025; Zheng *et al.* 2025). Within binding protein and peptide engineering, phage, yeast, and *E. coli* display formats (see Section Cell-based display (Owen T. Porth and K. Dane Wittrup^*^)) compatible with ncAAs are now available; these are complementary to mRNA display. Both systematic studies and high throughput discovery with these platforms advance our understanding of how to expand the properties of antibodies and other binders with well-positioned chemistries (Hampton and Liu 2024; Lino *et al.* 2024). There are a growing number of ncAA-containing proteins that may be commercially relevant, including ADCs, covalent binding proteins, and enzymes (Walsh *et al.* 2021; Alcala-Torano *et al.* 2022; Birch-Price *et al.* 2024; Cao and Wang 2024). Manufacturing platforms suitable for large-scale production of ncAA-proteins exist (Roy *et al.* 2020), but will require further improvements to facilitate more widespread use. Finally, are there roles for designing functional ncAA-containing proteins with rapidly emerging computational tools? This is still unclear, because the massive datasets used to train the current design tools do not exist for ncAA-proteins. Additional data linking ncAA-protein sequence and function will be important for training and validating ncAA-compatible design tools. NcAAs and many other subdisciplines of protein engineering are advancing rapidly, providing opportunities for improved ncAA incorporation systems to synergize with a tremendous range of experimental and computational tools. From pinpointed biochemical dissections to biocatalysis and drug discovery, proteins chemically augmented with ncAAs offer unique and increasingly accessible opportunities to dramatically expand protein functions.

### Chemigenetic strategies (Michelle S. Frei^*^)

**Figure 40:**
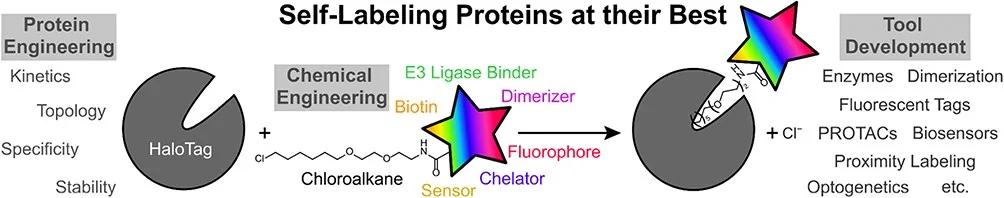
Self-labeling proteins (SLPs) at their best. SLPs are engineered proteins that have been optimized for specific and rapid reaction with a biologically stable and compatible moiety such as a chloroalkane in the case of the HaloTag SLP.

Chemigenetic strategies combine synthetic small molecules with genetically encoded proteins, expanding the repertoire of functional groups available in protein engineering and consequently the achievable protein function. Among the most versatile chemigenetic strategies are self-labeling proteins (SLPs) such as SNAP-tag and HaloTag, which form covalent bonds with benzylguanine (Keppler *et al.* 2003) and chloroalkane ligands (Los *et al.* 2008), respectively (Fig. 40). These ligands can carry diverse functionalities including synthetic fluorophores, sensors, or chelators, turning SLPs into adaptable building blocks for molecular tools such as chemical dimerizers, PROTACs, and optogenetic actuators (Hoelzel and Zhang 2020). Over the years, the reaction kinetics, thermostability, solubility, and substrate specificity of SLPs have been optimized and topological variants such as split and circularly permuted variants have been introduced (Porzberg *et al*. 2025). In the following paragraphs, I highlight some key advances in SLP engineering and their applications in molecular tool design particularly for fluorescence visualization.

The combination of SLPs with bright, photostable fluorophores have long advanced fluorescence microscopy. However, more recent engineering of the fluorophores themselves focusing on properties such as fluorogenicity and cell-membrane permeability (Grimm and Lavis 2022) has opened up new avenues. In addition, new structural insights and kinetic measurements of SLPs with rhodamine fluorophores (Wilhelm *et al.* 2021) have enabled tailored protein engineering. For instance, SNAP-tag2 was introduced offering up to 100× fold faster labeling kinetics and markedly improved brightness with rhodamine fluorophores compared to its predecessor (Kühn *et al.* 2025). HaloTag on the other hand has been engineered to yield distinct fluorescence lifetimes when labeled with the same fluorophore enabling multiplexed imaging of three targets in a single spectral channel *via* fluorescence lifetime imaging microscopy (FLIM) (Frei *et al.* 2022).

Early on SLPs were implemented in FRET-based biosensors (see Section Fluorescent proteins (Sohum Mehta and Jin Zhang^*^)) such as SNIFITs, which use a fluorophore tethered to a protein binding ligand. This allowed for augmentation of not only the biosensor’s spectral properties but also the panel of accessible analytes (Porzberg *et al*. 2025). More recent designs pair SLPs with FPs (ChemoG) (Hellweg *et al.* 2023) or retinal chromophores (Voltron) (Abdelfattah *et al.* 2019) enhancing the spectral versatility, dynamic range, and photostability of FRET-based biosensors. In addition, HaloTag was implemented in single fluorophore biosensors including (W)HaloCaMP (Deo *et al.* 2021; Farrants *et al.* 2024) and HaloGFP-Ca (Zhu *et al.* 2023b) exploiting covalent binding of a fluorophore or metal chelator to generate powerful biosensors with ideal properties such as high multiplexing capacity and far-red spectral properties.

SNAP-tag and HaloTag have long been workhorse tools in fluorescence microscopy and their integration into biosensors is accelerating with powerful chemigenetic biosensors and integrators emerging (Huppertz *et al.* 2024). Further, non-covalent SLP approaches (Kompa *et al.* 2023) push the limits of microscopy and reshape the design strategies of chemigenetic approaches. Tools beyond fluorescence benefit from the achievements and knowledge gained with fluorophores and are rapidly catching up. Finally, both the protein and the chemical ligand can be engineered creating synergies that promise unprecedented functionality.

### Chemically synthesized proteins (Guo-Chao Chu and Lei Liu^*^)

**Figure 41:**
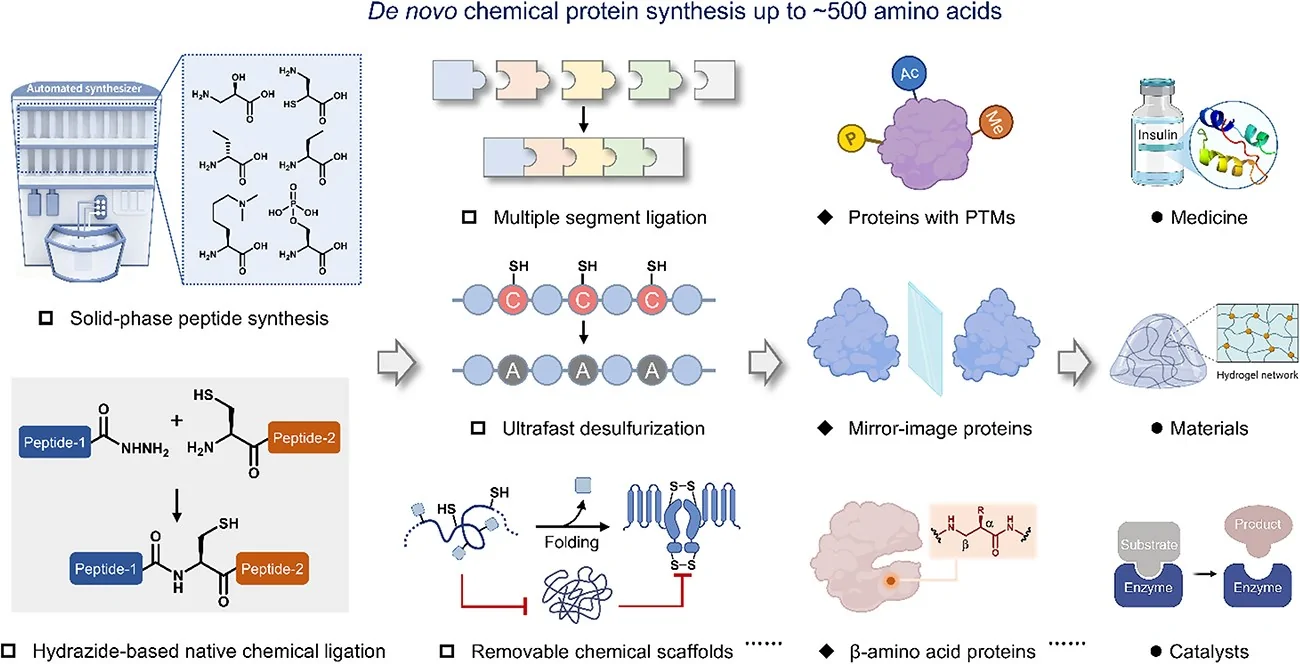
Conceptual diagram of workflows in modern chemical protein synthesis. Peptide segments, obtained from SPPS, are covalently assembled *via* chemoselective ligation and then folded into target proteins. Chemical protein synthesis offers an atomic-resolution approach for accessing proteins beyond natural amino acid limits, thus generating structurally novel peptide/protein drugs, peptide/protein materials, and artificial enzyme catalysts.

Chemical protein synthesis originated with Emil Fischer, who pioneered polypeptide synthesis *via* in-solution amino acid coupling (Kent 2009). Later, Merrifield developed solid-phase peptide synthesis (SPPS) for assembling polypeptides on insoluble resins. This eliminated tedious intermediate purification and greatly accelerated synthesis workflows (Kent 2025). However, SPPS has limitations: it cannot cost-effectively produce proteins over 60–80 amino acids while ensuring consistent purity. This drove the development of segment condensation-based protein synthesis. A key breakthrough was Kent *et al.*’s native chemical ligation (Dawson *et al.* 1994), which enables chemoselective ligation of two unprotected peptide segments: one with a C-terminal thioester and the other with an N-terminal cysteine (see Section Protein ligation (Luis F. Guerra and Manuel M. Müller^*^)). This laid a critical foundation for modern chemical protein synthesis (Fig. 41).

Methodological innovations have expanded the scope and capabilities of chemical protein synthesis. Notably, *in situ* activatable peptide hydrazides have emerged as practical surrogates for unstable peptide thioesters in ligation (Fang *et al.* 2011). This easy-to-use method enables flexible, high-yield, and cost-effective assembly of multiple peptide segments *via* convergent or one-pot strategies (Fang *et al*. 2012; Zheng *et al.* 2013; Tang *et al.* 2015), boosting practicality, and efficiency. Additionally, other ligation methods (e.g. STL: serine/threonine ligation; KAHA: α-keto acid-hydroxylamine ligation; and DSL: diselenide-selenoester ligation) have been explored to advance the field (Agouridas *et al.* 2019).

Leveraging peptide hydrazides’ broad chemical compatibility, various customizable, removable scaffolds (e.g. solubilizing tags, temporary sugar modifications) can be easily integrated into intermediates. These scaffolds modulate solution behavior, facilitating the synthesis of poorly soluble, highly aggregating, or hard-to-fold proteins (Zheng *et al.* 2014; Shi *et al.* 2022). Combined with emerging technologies (e.g. ultrafast desulfurization, flow chemistry-based SPPS, enzymatic transpeptidation), *de novo* synthesis now enables production of proteins with more complex structures and larger molecular weights. To date, the largest fully synthesized single-chain protein *via* this approach is polyubiquitinated α-globin (472 amino acids) (Sun *et al.* 2019). The largest noncovalently assembled fully synthesized protein is mirror-image T7 RNA polymerase (883 amino acids), built from three separately synthesized segments (Xu and Zhu 2022).

Robotics and AI show promise for advancing chemical protein synthesis (see Section Automation and robotics (Nilmani Singh and Huimin Zhao^*^)). Developing new synthetic chemistries and integrating them with automated synthesis robots could boost process efficiency and throughput. AI may also integrate structural and property data from chemically synthesized proteins containing unnatural or mirror-image amino acids—aid in designing peptide/protein drugs, materials, and artificial enzymes with functions exceeding natural amino acid-based counterparts. Overall, chemical protein synthesis offers a route to generate “beyond-biology” proteins across an unlimited chemical space, opening a new chapter for protein applications in human well-being.

## Summary and outlook

When protein engineering began to coalesce as a field in the 1980s, its future trajectory was far from obvious. It would have been entirely reasonable at the time to assume that a full solution to the protein folding problem would be a necessary prerequisite for rational protein design, and equally reasonable to imagine that such a solution might remain perpetually out of reach. At the same time, it would have been entirely reasonable to be uncertain about the limits of protein function. It was not clear how far proteins might be pushed beyond the chemistry explored by natural evolution, as enzymes or otherwise. Indeed, early reviews of the field were understandably focused on the prospects for using protein engineering to determine structure–function relationships in natural proteins, to recombinantly express natural proteins, and to improve existing properties of proteins (e.g. thermal stability, pH dependence, solubility, substrate specificity, and catalytic efficiency), rather than the creation of entirely new functions (Ulmer 1983; Kaiser and Lawrence 1984; Nagai 1985; Leatherbarrow and Fersht 1986; Wetzel 1986).

As the progress described in this status report reveals, after more than 40 years, we now have clear answers to these early uncertainties. We did not need to solve the classically defined protein folding problem, understood as the computation of all physical forces and folding pathways, in order to achieve *de novo* protein design with useful and unprecedented properties (Dill and MacCallum 2012). Instead, the combination of vast sequence and structural databases with advances in ML allowed the field to bypass this challenge in ways that would have been difficult to anticipate. Similarly, we do not cease to be amazed by the breadth of functions that can be undertaken by engineered proteins. Apparent constraints have repeatedly revealed themselves to be technical rather than fundamental, yielding to improved methods, better models, and creative experimental strategies.

A central message that emerges from this status report is the extraordinary pace of recent progress driven by ML and AI-based approaches. In areas such as the design of new protein folds and new-to-nature enzymes, the impact has already been transformative. At the same time, the report conveys a strong sense of anticipation for accelerated future progress in areas in which AI has not yet played a dominant role, such as ASR, IVDs, and biological logic gates, to name a few. Another takeaway message from this status report is that the rapid expansion of AI-enabled approaches has not supplanted many traditional strategies and is unlikely to ever do so. Rational design, evolutionary methods, and bioinformatics-based engineering continue to demonstrate remarkable utility and durability. While these methods will likely become increasingly guided and augmented by ML and AI, they will also continue to advance and mature independently of ML and AI-based approaches. For the foreseeable future, young researchers entering the field will have an incredible breadth of opportunities to explore and grand challenges to tackle.

The catalytic power of enzymes remains one of the great achievements of natural evolution, and the reliable engineering of artificial enzymes with catalytic efficiencies comparable to those of natural enzymes remains a grand challenge for the field. Despite important advances in enzyme design and optimization, in many cases we still do not fully understand why successful designs work, or why closely related variants fail. This gap in understanding likely reflects the importance of difficult-to-characterize properties that extend beyond static structures, including conformational dynamics coupled to the catalytic cycle, carefully evolved electric fields within active sites, and long-range interactions that shape catalytic function. A major challenge for the next decade will therefore be not only to design increasingly capable enzymes, but also to use protein engineering itself as a tool for developing a more fundamental understanding of catalysis. ML and AI-based approaches, together with steadily improving physics-based computational enzymology methods, may play complementary roles in linking predictive design with mechanistic understanding.

Another major challenge will be the discovery, implementation, and validation of design rules for the production of predominantly disordered, signal-integrating proteins, as exemplified by eukaryotic transcription factors. Unlike globular proteins that can often be abstracted in relatively simple terms, these systems are information-dense and governed by indeterminate, “fuzzy” logic arising from multivalent, low-affinity interactions, extensive PTMs, and emergent behaviors such as phase separation. The ability to understand, manipulate, and ultimately mimic such proteins would transform our understanding of biological regulation and open up many new possibilities for engineering and intervention. Achieving this will require integrating advances across many of the methods described in this review, including computational design, experimental methods, and evolutionary approaches. Fundamentally, it also entails learning to incorporate diverse functionalities into single polypeptide chains in a manner that maximizes information density and throughput.

Yet another grand challenge in the area of therapeutic development is immunogenicity, which remains a common obstacle preventing many elegant protein engineering concepts from reaching the clinic. Current strategies largely restrict therapeutic protein components to “self,” i.e. human domains, relying on the thymus to have eliminated T cells that recognize self-peptides, but this represents a major constraint because most of sequence space is “non-self.” The key question is whether it will become possible to develop general strategies for creating fully non-immunogenic proteins while keeping protein function intact. Achieving this goal may require quantitative models for the full process of antigen presentation, including protein uptake, unfolding, peptide generation and transport, MHC binding, and interactions with the T-cell repertoire of an individual. At present, it remains unclear to what extent such comprehensive modeling will ever be achievable, but success in this area would have a profound impact on the development of future medicines.

Looking ahead, we envision a sustained positive feedback cycle in which computational advances accelerate experimental discovery, and experimental insights in turn refine computational models. Purely AI- and ML-driven approaches may eventually encounter fundamental limitations that can only be overcome with deeper integration with physics-based computational approaches. Through this interplay, protein engineering will continue to be a uniquely powerful discipline, delivering research tools, therapies, cleaner industrial processes, and economic prosperity, to the benefit of all humans and the environment.

## Data Availability

No new data were generated or analysed in support of this research.

## Notes

Editor: Evamaria I. Petersen

Correspondence related to a specific section should be addressed to the senior author of the section, as indicated with an asterisk.
